# Decoding the hub gene blueprint for broiler liver development: A synergistic histomorphological and transcriptomic investigation during the neonatal phase

**DOI:** 10.1016/j.psj.2026.106938

**Published:** 2026-04-15

**Authors:** Xiaofeng Li, Abdel-Moneim Eid Abdel-Moneim, Kewei Fan, Bing Yang

**Affiliations:** a College of Animal Science and Technology, Ningxia University, Yinchuan 750021, China; b Longyan University & Fujian Provincial Key Laboratory for Prevention and Control of animal Infectious Diseases and Biotechnology, Longyan University Longyan, China; c Biological Applications Department, Nuclear Research Center, Egyptian Atomic Energy Authority, Abu Zaabal 13759, Egypt

**Keywords:** Hub genes, Liver, Development, Chicken, Neonatal phase

## Abstract

To investigate the morphological transitions and the role of hub genes during the first week of liver development in broilers. At hatch (D0) and 7 days post-hatch (D7), five broiler chickens were humanely euthanized and liver samples were collected to assess liver histomorphology and index. The gene expression data of liver of broilers at D0 and D7 obtained from the GEO database. Differentially expressed genes (DEG) analyses were conducted using GEO2R. Function enrichment for DEGs was conducted using the DAVID and PANTHER databases. Hub genes were identified using the CytoHubba plugin in Cytoscape, employing four different methods: Degree, MCC, ENC, and Closeness. Statistical analyses were conducted using IBM SPSS Statistics. We observed significant developmental changes: liver weight surged by 250.69% (*P* < 0.01), accompanied by a 75.85% rise in the liver index (*P* < 0.01). In addition, the histological examination from D0 to D7 reveals distinct morphological transformations. Transcriptomic profiling revealed 1,762 DEGs, comprising 974 up-regulated and 788 down-regulated candidates. Functional enrichment analysis suggested that DEGs participated in fatty acid synthesis and metabolism, cholesterol metabolic process, mitotic cell cycle, as well as MAPK, PPAR, steroid biosynthesis, and metabolic signaling pathway. Notably, six downregulated hub genes (e.g., *KIF11* and *MCM2*) were identified as central regulators of liver maturation. Functional enrichment analysis revealed their pivotal roles in core biological processes—such as double-strand break repair, DNA replication initiation, and mitotic cell division—as well as critical pathways including cell cycle, DNA replication, and cellular senescence. These findings establish hub genes coordinates liver morphogenesis, providing mechanistic insights for optimizing poultry liver health and nutritional strategies.

## Introduction

The liver functions as the central metabolic hub in vertebrates, orchestrating critical physiological processes including nutrient metabolism, xenobiotic detoxification, protein biosynthesis, and energy homeostasis (Martins et al., 2024; Zhang et al., 2024). Within intensive broiler production systems, optimal hepatic performance is fundamental for achieving superior feed conversion efficiency, sustained growth trajectories, and comprehensive flock health (Wang et al., 2015; Anene et al., 2023). Decades of intensive genetic selection for rapid growth have imposed extraordinary metabolic demands on broiler hepatocytes (Willson et al., 2017), particularly during the critical peri-hatch transition when birds shift metabolic substrates from yolk-derived lipids to exogenous carbohydrate-rich diets (Hicks et al., 2022). Consequently, deciphering the molecular mechanisms governing hepatic maturation during this developmental window is paramount for designing nutritional and management interventions that optimize both productivity and animal welfare.

Contemporary research underscores hub genes—highly interconnected nodes within transcriptional regulatory networks—as pivotal coordinators of hepatic development. While mammalian studies have identified master regulators of hepatogenesis, metabolic zonation, and functional specialization (Li et al., 2025), the hub genes directing the accelerated hepatic development occurring during the first post-hatch week (D0-D7) in broilers remain poorly characterized. Existing evidence implicates evolutionarily conserved genes regulating core cellular machinery: cell cycle progression (e.g., cyclins), DNA replication (e.g., minichromosome maintenance complex components MCM2/5/6) (Liu et al., 2024; Zhao et al., 2022), and mitotic control. Specifically, the MCM helicase complex facilitates DNA origin licensing and replication fork progression (Nasheuer and Meaney, 2024), while *KIF11* (a kinesin motor protein) and *CDC45* (a scaffolding component of the CMG helicase) orchestrate mitotic spindle dynamics and replisome assembly, respectively (Cai et al., 2025; You and Masai, 2024). Dysregulation of these molecular systems correlates with developmental abnormalities across tissues (Li et al., 2024; Mohamed et al., 2025), highlighting their potential as master regulators of hepatic morphogenesis.

This study aims to comprehensively map transcriptomic reprogramming and identify pivotal hub genes governing the profound morphological and functional transformation of broiler liver during the D0-D7 developmental window. We hypothesize that specific hub genes coordinate the metabolic transition and rapid parenchymal expansion characteristic of this phase. To test this, we will integrate histomorphometric analysis of hepatic architecture with comparative transcriptomics of D0 and D7 liver tissues, employing differential gene expression analysis coupled with functional enrichment interrogation of identified DEGs. Elucidating these regulatory networks addresses a critical knowledge gap in avian hepatic biology, with significant implications for poultry science—potentially enabling precision nutrition strategies targeting metabolic efficiency, novel biomarkers for gut-liver axis health assessment, and sustainable intensification of broiler production systems.

## Materials and methods

### Avian subjects and husbandry

Ten male Ross 308 broiler chicks (Bengbu Dacheng Food Co.) were housed in climate-controlled facilities under progressive thermal adaptation: initial temperature 32-34°C (Week 1) decreasing weekly by 2°C to 24°C (Week 3). Environmental conditions were maintained at 60-65% relative humidity with a photoperiod transition from 18L:6D to 23L:1D. Birds received ad libitum access to water and NRC 2014-compliant starter diet (Anhui Baixin Feed Co., Table 1), with routine health monitoring and immunization protocols implemented.

**Table 1:** Dietary components and their nutritional values.

Item	Content/%
Ingredients	
Corn	41.50
Soybean meal	23.60
Rice polishing meal	8.80
Soybean meal	3.20
Wheat	8.00
Rice bran	5.00
Spray-dried corn gluten feed	2.00
Corn starch residue	3.00
Feather meal	1.00
Limestone	1.80
Dicalcium phosphate	0.50
Montmorillonite	0.30
Sodium chloride	0.30
Lysine	0.49
Methionine	0.24
Threonine	0.02
Choline chloride	0.10
VTR Enzyme 818	0.03
Phytase	0.02
Sodium bicarbonate	0.10
Total	100%
Nutrients levels^1^	
Metabolizable energy/Mcal·kg^-^1	2.90
Moisture	9.74
Crude protein	19.22
Crude fat	3.07
Crude fiber	5.38
Crude ash	10.05
Lysine	1.35
Methionine	0.60
Methionine + Cystine	0.80
Calcium	0.88
Available phosphorus	0.40

1 Content of metabolizable energy, methionine, cystine, calcium, available phosphorus in diet was calculated values, while moisture, crude protein, fat, fiber, ash was measured values.

### Hepatic developmental analysis

Liver specimens were collected at two developmental stages: hatch day (D0) and 7 days post-hatch (D7) (*n* = 5/group). Following GB/T 39760-2021 standards (China), birds were euthanized via sodium pentobarbital injection (150 mg/kg, i.p.). Excised livers underwent PBS perfusion, gravimetric assessment (liver index = [liver mass/body mass] × 100), and fixation in 10% neutral buffered formalin prior to paraffin embedding (5 μm sections). Histomorphometric analyses quantified hepatocyte morphology, lobular architecture, and vascular components using digital imaging. Statistical comparisons of liver mass and liver index were conducted using the independent samples t-test in SPSS 25 (IBM, New York). The homogeneity of variances was assessed using Levene’s test, and the t-test results were selected accordingly based on the assumption of equal variances. Significance levels were defined as *P* < 0.05 (*) and *P* < 0.01 (**).

### RNA isolation and microarray quality control procedures

Hepatic RNA was extracted under controlled conditions using TRIzol® reagent (Invitrogen). A pooled design was employed, in which 12–15 biological replicates per developmental stage (D0/D7) were combined to generate three composite samples per time point (each representing 4–5 individual extractions). RNA integrity was assessed using dual methods: an Agilent 2100 Bioanalyzer confirmed RNA Integrity Numbers (RIN) > 8.0, and spectrophotometric analysis verified A260/A280 ratios within the range of 1.8–2.0.

Qualified RNA (2 μg) was reverse-transcribed using T7-oligo(dT)24 primers (Invitrogen). Subsequent enzymatic steps included RNase H treatment, second-strand synthesis with DNA Polymerase I and ligase, and phenol-chloroform purification. Biotinylated cRNA (≥20 μg) was generated via in vitro transcription using the Affymetrix IVT Kit, followed by controlled fragmentation (94°C for 35 min in pH 8.1 buffer). Hybridization mixtures, each containing 15 μg of fragmented cRNA in MES buffer, were supplemented with spike-in controls (BioB, BioC, BioD, and Cre) and processed under standardized conditions (45°C for 16 h at 60 rpm).

Post-hybridization protocols comprised: (i) stringent washing (50°C, 6 × SSPE), (ii) non-stringent washing (25°C), and (iii) streptavidin-phycoerythrin staining. Fluorescence detection employed a GeneChip® Scanner 3000 (570 nm excitation, 3 μm resolution). Data processing via GCOS 1.1 software implemented MAS 5.0 normalization (median target=500). The MIAME-compliant dataset (GSE15413) and platform details (GPL3213) are publicly accessible through NCBI GEO.

### Transcriptomic data acquisition

The current investigation incorporated the publicly available gene expression dataset GSE15413, retrieved from the NCBI GEO repository (https://www.ncbi.nlm.nih.gov/geo/). This curated dataset comprises six avian duodenal specimens, equally distributed between neonatal (D0; GSM386831-833) and 7-day post-hatch (D7; GSM386834-836) developmental stages. Complementary data on histone phosphorylation-associated genes were systematically extracted from the GeneCards human gene database (Version 4.14; https://www.genecards.org/).

### Identification of differentially expressed genes

Gene expression differential analysis was conducted using the GEO2R analytical platform (NCBI), which incorporates the Limma package optimized for microarray data processing. Statistical significance thresholds were established with *P* < 0.05 and absolute log2 fold change ≥1.00. Only probe sets with valid Entrez Gene identifiers were retained for downstream analyses.

### Functional annotation and protein categorization

The functional characterization of the DEGs was conducted through integrated bioinformatics analyses. Gene Ontology (GO) term enrichment, KEGG pathway mapping, and Reactome pathway evaluation were performed using the DAVID platform (v6.8). Complementary functional classification was carried out via the PANTHER system (v16.0), which also facilitated systematic categorization of DEG-encoded proteins according to their molecular functions and biological roles within the protein class ontology framework. All analyses employed standardized statistical thresholds (*P* < 0.05) to ensure biological relevance.

### Protein interaction network analysis and hub gene characterization

Protein-protein interaction networks were reconstructed using the STRING database (Version 11.0; https://string-db.org/) with subsequent visualization and topological analysis conducted in Cytoscape 3.8.0. Network centrality analysis was performed through the CytoHubba plugin, employing four distinct algorithms: MCC, Degree, EPC, and Closeness. For each algorithm, the top 15 genes ranked by score were identified, and the overlapping genes across all four methods were selected as consensus hub genes. These hub genes were then subjected to functional annotation through integrative analysis of published literature, NCBI Gene resources, and GeneCards annotations.

## Results

### Liver histomorphology in the early development

**Fig. 1:**
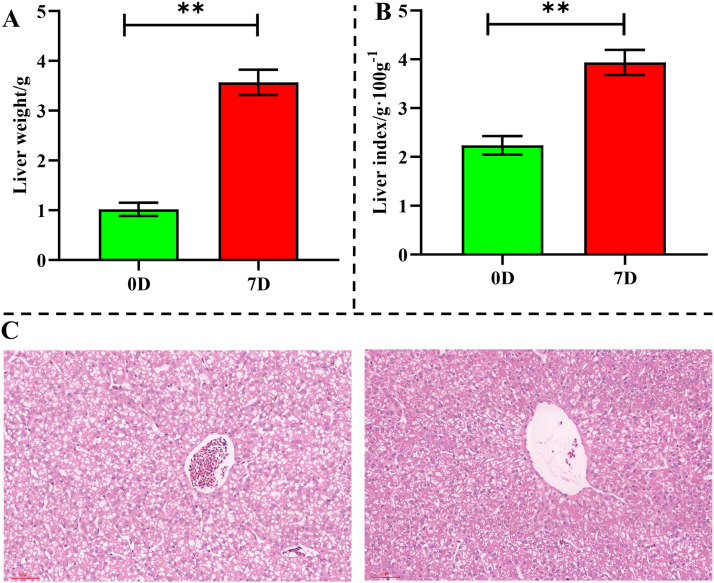
**Histomorphological changes in the liver during early post-hatching development. (A)** and **(B)** show the changes in liver weight and liver index of broiler chickens, respectively. The liver exhibited a 2.5-fold increase in mass (250.69%, *P* < 0.01, **), accompanied by a 75.85% rise in the hepatosomatic index (*P* < 0.01, **), indicating enhanced hepatic development during this critical developmental window. **(C)** illustrates the histomorphology of chicken liver tissue (H&E staining) at D0 and D7, with the left and right panels representing D0 and D7, respectively. At D7, hepatocytes displayed hyperplastic proliferation and hypertrophic enlargement, along with increased cytoplasmic and nuclear staining. Hepatic lobules appeared more distinctly organized, featuring mature central vein–sinusoid networks and progressive interstitial fibrosis.

Liver mass increased significantly by 250.69% (Fig. 1A; *P* < 0.01) while the liver index rose 75.85% (Fig. 1B; *P* < 0.01), indicating accelerated organogenesis. Histological examination (HE staining) revealed progressive morphological transformations from D0 to D7 (Fig. 1C). Hepatocytes showed both hyperplastic and hypertrophic changes, including increased cell numbers, cellular enlargement, and heightened cytoplasmic/nuclear staining intensity. Hepatic architecture became progressively organized: initially indistinct lobular structures developed clear demarcation, with central veins and sinusoids maturing from rudimentary structures to defined vascular networks. Concurrently, connective tissue components increased in the hepatic interstitium. These morphological transitions collectively demonstrate fundamental developmental processes including cellular proliferation, structural specialization, and functional maturation during this critical postnatal phase.

### Overview of genes expression in chicken livers

**Fig. 2:**
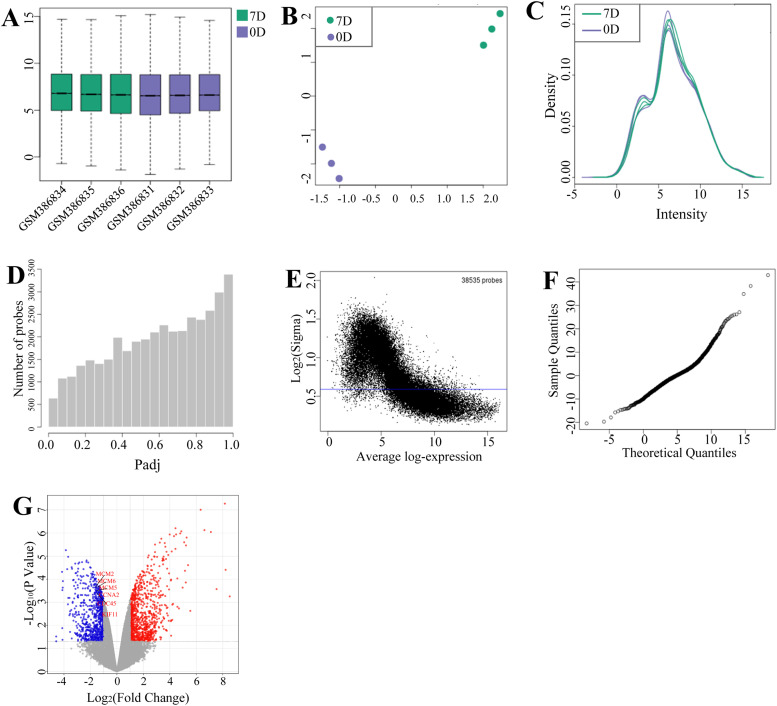
**Transcriptomic profiling of hepatic development. (A)** gene expression box plots; **(B)** UMAP; **(C)** expression density; **(D)** adjusted P-value counts; **(E)** mean-variance trend; **(F)** moderated T statistic. **(G)** volcano plot for the DEGs identified in chicken liver between D7 and D0. Red, blue, and gray dots denote genes significantly upregulated, downregulated, and exhibiting non-significant changes at D7, respectively. Additionally, the labeled genes are potential hub genes related to liver development.

RNA-seq analysis identified 38,535 transcripts and 12,633 protein-coding genes in chicken hepatic tissues at developmental stages D0 and D7. Comparative transcriptomics revealed 1,762 differentially expressed genes (DEGs), with 974 upregulated and 788 downregulated in D7 samples relative to D0 (Supplementary File 1; Table 2). The most prominent DEGs included upregulated metabolic regulators (IGF1, THRSP, SCD, FADS2) and cell cycle inhibitor CDKN2B (Table 3), alongside downregulated metabolic markers (GCG, FABP2) and developmental regulators (LHFPL5, DIO3) (Table 4). Comprehensive visualization included: (i) expression distribution metrics (box plots, UMAP, density plots); (ii) statistical validation (P-value distribution, mean-variance relationship, moderated T-statistics) (Fig. 2A-F); and (iii) a volcano plot highlighting DEG magnitude and significance (Fig. 2G).

**Table 2:** Spatiotemporal dynamics of hepatic transcriptome: DEGs in broiler chickens during neonatal transition (D0 vs. D7).

Total	Up-regulated	down-regulated
1,762	974	788

**Table 3:** Top 30 significantly upregulated hepatic genes during post-hatch transition (D0 vs. D7).

Gene symbol	Log_2_FC	*P* Value	Full name
*FADS2*	8.53	5.58 × 10^-4^	Fatty acid desaturase 2
*SCD*	8.22	3.93 × 10^-5^	Stearoyl-CoA desaturase
*THRSP*	8.16	5.32 × 10^-8^	Thyroid hormone responsive
*CDKN2B*	7.53	2.70 × 10^-4^	Cyclin-dependent kinase inhibitor 2B
*IGF1*	7.10	9.04 × 10^-7^	Insulin like growth factor 1
*FADS1*	6.62	7.49 × 10^-7^	Fatty acid desaturase 1
*SQLE*	6.33	9.79 × 10^-8^	Squalene epoxidase
*PIGR*	5.55	2.36 × 10^-3^	Polymeric immunoglobulin receptor
*AGMO*	5.38	2.47 × 10^-5^	Alkylglycerol monooxygenase
*DHCR24*	5.27	1.57 × 10^-6^	24-dehydrocholesterol reductase
*TBC1D8*	5.22	3.45 × 10^-6^	TBC1 domain family, member 8
*OCX36*	5.20	1.39 × 10^-4^	BPI fold containing family B, member 3
*CDO1*	5.15	4.33 × 10^-5^	Cysteine dioxygenase type 1
*ELOVL2*	5.08	2.52 × 10^-6^	ELOVL fatty acid elongase 2
*JCHAIN*	4.97	2.32 × 10^-4^	Joining chain of multimeric IgA and IgM
*BHLHE41*	4.87	8.44 × 10^-7^	Basic helix-loop-helix family member e41
*LSS*	4.79	1.04 × 10^-6^	Lanosterol synthase
*ACE2*	4.69	1.85 × 10^-3^	Angiotensin I converting enzyme 2
*FGF23*	4.57	1.57 × 10^-3^	Fibroblast growth factor 23
*KIAA1958*	4.57	9.34 × 10^-5^	KIAA1958
*GIF*	4.48	1.15 × 10^-6^	Gastric intrinsic factor
*NSDHL*	4.43	6.21 × 10^-7^	NAD(P) dependent steroid dehydrogenase-like
*GSTA4*	4.43	4.85 × 10^-6^	Glutathione S-transferase alpha 4
*IFI27L2*	4.32	1.37 × 10^-6^	Interferon, alpha-inducible protein 27-like 2
*BF2*	4.25	1.17 × 10^-3^	Major histocompatibility complex class I antigen BF2
*SLC14A2*	4.19	1.27 × 10^-3^	Solute carrier family 14, member 2
*AFP*	4.19	5.81 × 10^-3^	Alpha-fetoprotein
*FDFT1*	4.14	6.30 × 10^-6^	Farnesyl-diphosphate farnesyltransferase 1
*MAOB*	4.10	6.10 × 10^-3^	Monoamine oxidase B
*CP*	4.10	1.04 × 10^-4^	Ceruloplasmin

**Table 4:** Top 30 significantly downregulated hepatic genes during post-hatch transition (D0 vs. D7).

Gene symbol	Log_2_FC	*P* Value	Full name
*GCG*	-4.59	3.06 × 10^-2^	Glucagon
*FABP2*	-4.58	4.93 × 10^-2^	Fatty acid binding protein 2
*LHFPL5*	-4.14	4.82 × 10^-5^	Lipoma HMGIC fusion partner-like 5
*DIO3*	-4.13	1.33 × 10^-2^	Deiodinase, iodothyronine, type III
*ZBTB16*	-4.12	2.98 × 10^-4^	Zinc finger and BTB domain containing 16
*CTRC*	-4.11	4.26 × 10^-2^	Chymotrypsin C (caldecrin)
*VSTM2A*	-4.09	2.33 × 10^-4^	V-set and transmembrane domain containing 2A
*C1QTNF9*	-4.08	1.22 × 10^-4^	C1q and tumor necrosis factor related protein 9
*CHAC1*	-3.85	5.53 × 10^-6^	ChaC glutathione-specific gamma-glutamylcyclotransferase 1
*HOXB6*	-3.75	3.69 × 10^-5^	Homeobox B6
*ALX1*	-3.70	1.23 × 10^-3^	ALX homeobox 1
*SSX2IP*	-3.68	1.07 × 10^-5^	Synovial sarcoma, X breakpoint 2 interacting protein
*CYGB*	-3.67	6.50 × 10^-4^	Cytoglobin
*AvBD4*	-3.59	3.66 × 10^-3^	Avian beta-defensin 4
*FGF12*	-3.58	6.47 × 10^-4^	Fibroblast growth factor 12
*CNNM2*	-3.54	3.17 × 10^-3^	Cyclin M2
*ACACB*	-3.54	2.46 × 10^-3^	Acetyl-CoA carboxylase beta
*GAL3ST2*	-3.54	1.94 × 10^-2^	Galactose-3-O-sulfotransferase 2
*MALL*	-3.44	2.16 × 10^-3^	Mal, T-cell differentiation protein-like
*LINGO1*	-3.42	3.04 × 10^-5^	Leucine rich repeat and Ig domain containing 1
*UAP1L1*	-3.41	3.73 × 10^-2^	UDP-N-acteylglucosamine pyrophosphorylase 1-like 1
*CDON*	-3.39	3.55 × 10^-5^	Cell adhesion associated, oncogene regulated
*MMP9*	-3.35	1.07 × 10^-3^	Matrix metallopeptidase 9
*FST*	-3.34	9.57 × 10^-4^	Follistatin
*FBXO22*	-3.33	3.33 × 10^-3^	F-box protein 22
*LECT2*	-3.31	1.11 × 10^-4^	Leukocyte cell derived chemotaxin 2
*ZNF518B*	-3.31	3.94 × 10^-3^	Zinc finger protein 518B
*CASC5*	-3.28	6.27 × 10^-3^	Cancer susceptibility candidate 5
*TMTC1*	-3.26	1.07 × 10^-4^	Transmembrane and tetratricopeptide repeat containing 1
*AvBD1*	-3.24	1.97 × 10^-4^	Avian beta-defensin 1

### GO enrichment

**Fig. 3:**
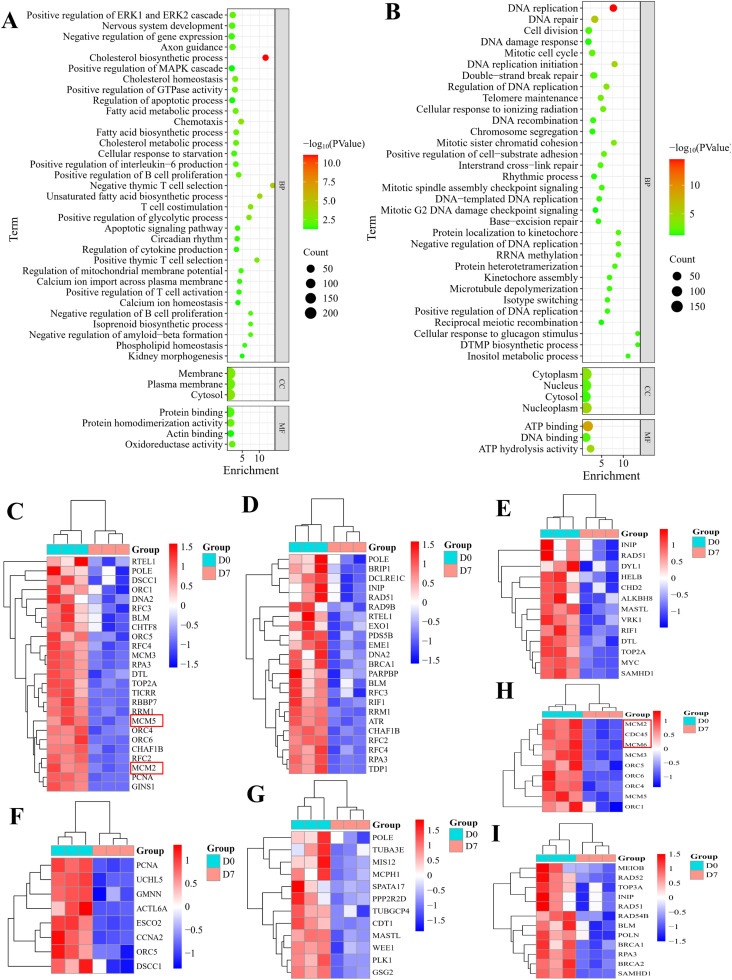
**Functional enrichment of the DEGs. (A)** GO terms enriched for upregulated DEGs in the duodenum at D7, including cholesterol metabolic process, axon guidance, fatty acid biosynthetic process, cholesterol homeostasis, fatty acid metabolic process, cholesterol biosynthetic process, chemotaxis, and unsaturated fatty acid biosynthetic process. **(B)** Enriched GO terms for downregulated DEGs, predominantly involving DNA replication, cell division, mitotic cell cycle, telomere maintenance, DNA recombination, chromosome segregation, mitotic sister chromatid cohesion, and inositol metabolic process. **(C-I)** illustrate the DEGs in the biological processes of DNA replication, DNA repair, DNA damage response, DNA replication regulation, mitotic cell cycle, DNA replication initiation, and double-strand break repair via homologous recombination, respectively. The genes highlighted with red borders were potential key genes for liver development.

To understand the biological processes associated with DEGs, GO enrichment analysis was conducted using the DAVID database. Upregulated DEGs (Fig. 3A; Supplementary File 2) were significantly enriched in 61 biological processes, predominantly involving cholesterol metabolic process, fatty acid biosynthetic process, cholesterol homeostasis, fatty acid metabolic process, chemotaxis, cholesterol biosynthetic process, and unsaturated fatty acid biosynthetic process. These genes were additionally associated with 21 cellular components and 31 molecular functions. Conversely, downregulated DEGs (Fig. 3B; Supplementary File 3) demonstrated enrichment in 47 biological processes, with prominent representation in DNA replication, DNA repair, cell division, mitotic cell cycle, DNA replication initiation, DNA replication regulation, telomere maintenance, DNA recombination, chromosome segregation, and inositol metabolic process. These alterations corresponded to 25 cellular components and 27 molecular functions. Additionally, Fig. 3C-H illustrated the DEGs in the biological processes of DNA replication, DNA repair, DNA damage response, DNA replication regulation, mitotic cell cycle, DNA replication initiation, and double-strand break repair via homologous recombination, respectively.

### KEGG enrichment

**Fig. 4:**
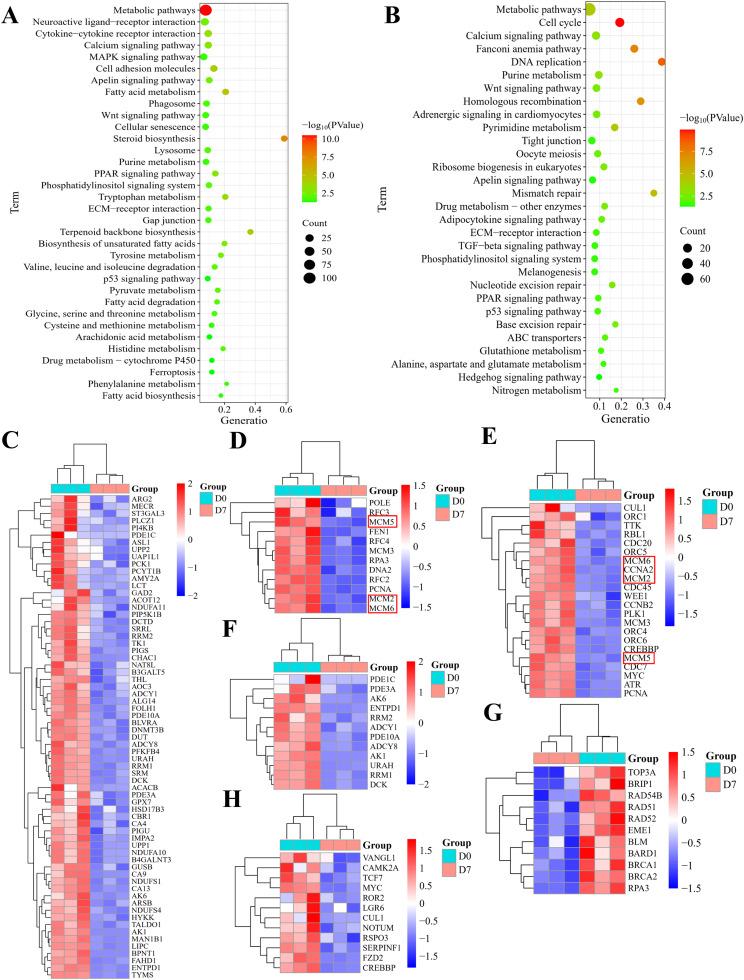
**Pathway enrichment analysis of the DEGs. (A)** KEGG pathways enriched for upregulated DEGs in the duodenum at D7, highlighting metabolic pathways, PPAR, Wnt, calcium, MAPK, phagosome, lysosome, unsaturated fatty acid biosynthesis, fatty acid degradation, cell adhesion molecules, fatty acid metabolism, ECM-receptor interaction, and fatty acid biosynthesis. **(B)** Pathways associated with downregulated DEGs, predominantly involving metabolic pathways, cell cycle, calcium, PPAR, Wnt, DNA replication, adipocytokine, purine metabolism, homologous recombination, tight junction, ribosome biogenesis, TGF-beta, and nucleotide excision repair. **(C-G)** present the DEGs within the signaling pathways of: metabolic processes, DNA replication, cell cycle, DNA replication, homologous recombination, and Wnt signaling. The genes marked with red borders represented potential key regulators of liver development.

To understand the signaling pathways associated with DEGs, KEGG enrichment analysis was conducted using the kobas database. Upregulated DEGs (Fig. 4A and Supplementary file 4) enrichment in 38 pathways, particularly involving metabolic pathways, PPAR, Wnt, calcium, MAPK, phagosome, lysosome, tryptophan metabolism, gap junction, terpenoid backbone biosynthesis, unsaturated fatty acid biosynthesis, fatty acid degradation, cell adhesion molecules, fatty acid metabolism, ECM-receptor interaction, and fatty acid biosynthesis. Furthermore, downregulated DEGs (Fig. 4B; Supplementary File 5) demonstrated involvement in 31 pathways, prominently featuring metabolic pathways, cell cycle, calcium, PPAR, Wnt, DNA replication, apelin, adipocytokine, homologous recombination, pyrimidine metabolism, tight junction, ribosome biogenesis, TGF-beta, and nucleotide excision repair. Additionally, Fig. 4C-J illustrated the DEGs in metabolic processes, DNA replication, cell cycle, DNA replication, homologous recombination, and Wnt signaling, respectively.

### Reactome pathway analysis

**Fig. 5:**
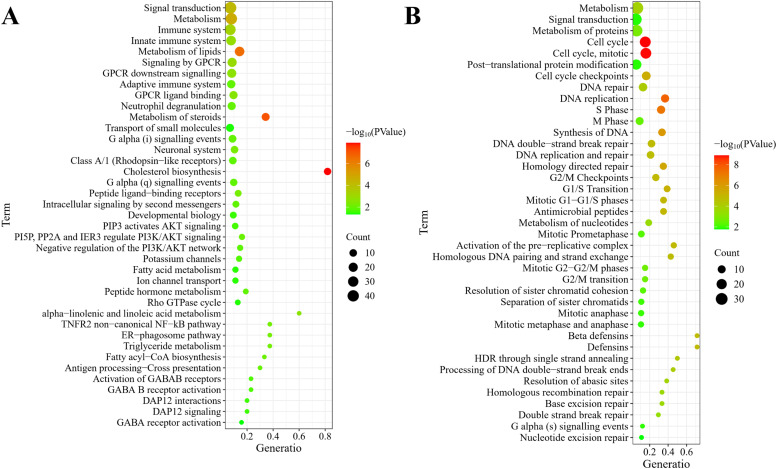
**Reactome pathway enrichment of the DEGs. (A)** Upregulated DEGs in D7 duodenum were enriched in signal transduction, metabolism, immune system, innate immune system, lipid metabolism, neutrophil degranulation, steroid metabolism, and small molecule transport. **(B)** Downregulated DEGs primarily involved metabolism, signal transduction, protein metabolism, cell cycle, cell cycle checkpoints, DNA repair, DNA replication, M phase, DNA synthesis, homology directed repair, G2/M checkpoints, and DNA replication and repair.

Reactome pathway analysis demonstrated extensive signaling network remodeling during liver development, where upregulated DEGs (Fig. 5A) were significantly enriched in 61 pathways, predominantly encompassing signal transduction, metabolism, immune system, innate immune system, lipid metabolism, signaling by GPCR, GPCR downstream signaling, adaptive immune system, GPCR ligand binding, neutrophil degranulation, steroid metabolism, and small molecule transport. Furthermore, downregulated DEGs (Fig. 5B) demonstrated involvement in 114 pathways, prominently featuring metabolism, signal transduction, protein metabolism, cell cycle, post-translational protein modification, cell cycle checkpoints, DNA repair, S Phase, DNA replication, M phase, DNA synthesis, homology directed repair, G2/M checkpoints, and DNA replication and repair.

### Protein classification

**Fig. 6:**
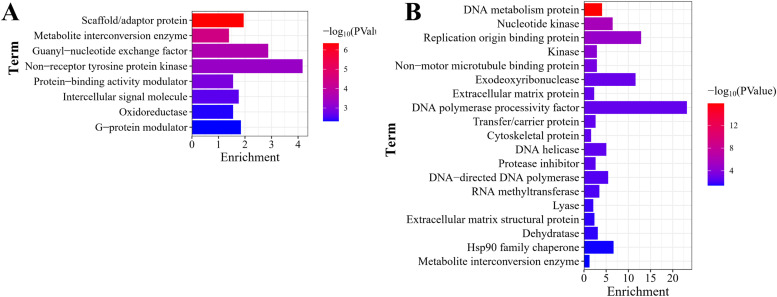
**Protein classification for the DEGs. (A)** Upregulated DEGs at D7 encoded proteins critical for metabolite interconversion enzyme, protein-binding activity modulator, oxidoreductase, G-protein modulator, guanyl-nucleotide exchange factor, non-receptor tyrosine protein kinase, et al. **(B)** Downregulated DEGs functionally centered on metabolite interconversion enzyme, cytoskeletal protein, lyase, protease inhibitor, kinase, dehydratase, RNA methyltransferase, DNA helicase, replication origin binding protein, and DNA polymerase processivity factor, et al.

To understand the proteins associated with DEGs, Protein classification analysis was conducted using the PANTHER database. The upregulated DEGs at D7 were associated with metabolite interconversion enzyme, protein-binding activity modulator, oxidoreductase, intercellular signal molecule, G-protein modulator, scaffold/adaptor protein, guanyl-nucleotide exchange factor, non-receptor tyrosine protein kinase, et al (Fig. 6A). Additionally, the downregulated DEGs in chicken liver at D7 were linked to metabolite interconversion enzyme, cytoskeletal protein, lyase, extracellular matrix protein, extracellular matrix structural protein, transfer/carrier protein, protease inhibitor, kinase, non-motor microtubule binding protein, dehydratase, RNA methyltransferase, DNA metabolism protein, DNA helicase, DNA-directed DNA polymerase, nucleotide kinase, Hsp90 family chaperone, exodeoxyribonuclease, and DNA polymerase processivity factor, et al (Fig. 6B).

### PPI network, hub genes and their functions

**Fig. 7:**
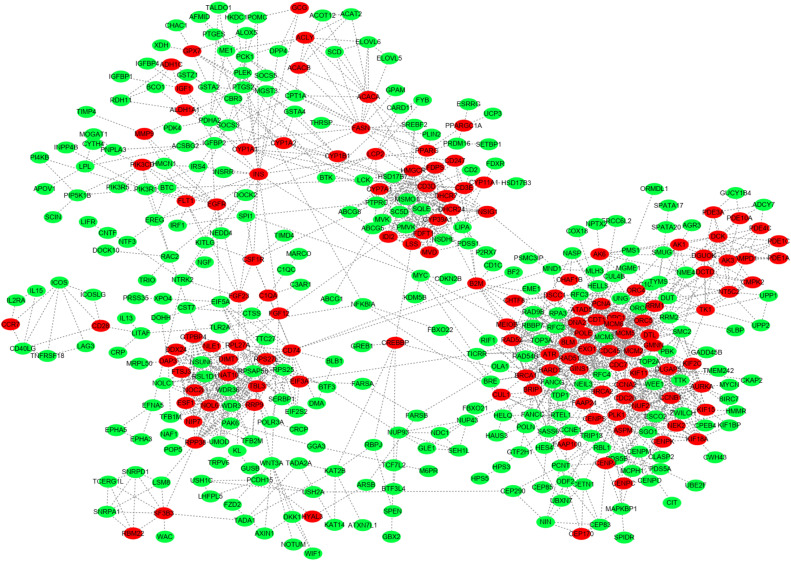
**Protein interaction networks for the DEGs.** PPI network analysis identified *CCNA2, CDC45, CYP39A1, CYP1A2, PPARG, KIF11, MCM2, MCM6*, and *MCM5*, in liver development.

To further identify key genes, we performed a protein-protein interaction analysis of the DEGs using STRING. As depicted in Fig. 7, among the DEGs, many genes*,* such as *CCNA2, CDC45, CYP39A1, CYP1A2, PPARG, KIF11, MCM2, MCM6, MCM5*, were implicated in the early development of the broiler livers.

**Fig. 8:**
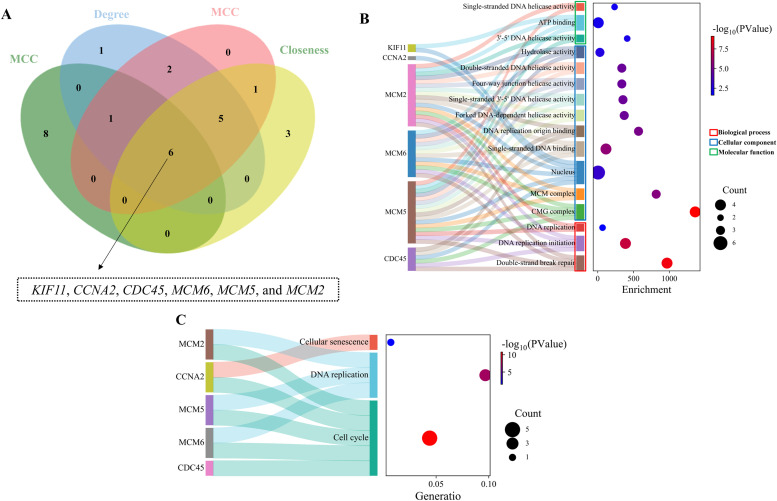
**Hub genes regulating liver development. (A) Hub genes for chicken liver development**. Six hub genes, including *KIF11, CCNA2, CDC45, MCM6, MCM5*, and *MCM2*, were associated with the early development of the broiler liver. **(B)** and **(C)** present GO and KEGG enrichment analyses of hub genes, respectively. Functional enrichment analyses identified three major biological process clusters among hub genes, with predominant involvement in Double-strand break repair, DNA replication initiation, and DNA replication. Concurrently, these genes participated in 3 core cellular components and 10 molecular functions. KEGG pathway mapping further demonstrated significant associations with cell cycle regulation, DNA replication, and cellular senescence.

To further identify hub genes, we analyzed the PPI network using the CytoHubba plugin in Cytoscape, employing four algorithms. The common genes identified by these four algorithms were designated as hub genes. As illustrated in Fig. 8A, among the DEGs, *KIF11, CCNA2, CDC45, MCM6, MCM5*, and *MCM2* were associated with the early development of the broiler liver. GO enrichment analysis revealed that these hub genes were significantly enriched in 3 biological processes, predominantly involving double-strand break repair, DNA replication initiation, and DNA replication (Fig. 8B). These alterations corresponded to 3 cellular components and 10 molecular functions (Fig. 8B). KEGG enrichment analysis revealed that these hub genes linked to cell cycle, DNA replication, and cellular senescence signaling pathways (Fig. 8C). In addition, hub gene functions were shown in Table 5. For instance, the protein encoded by *CCNA2* belongs to the ‌highly conserved cyclin family‌, whose members function as ‌essential regulators of cell cycle progression‌. ‌This cyclin‌ binds and activates cyclin-dependent kinase 2 (CDK2), ‌thereby driving progression through the G1/S and G2/M phase transitions (Table 5)‌. *KIF11* is a motor protein that ‌converts‌ chemical energy (typically through ATP hydrolysis) into mechanical force. ‌This protein‌ acts upon microtubules to transport vesicles and organelles from the cell center to the periphery and drive the beating of flagella and cilia. In non-mitotic cells, *KIF11* ‌is required‌ for transporting secretory proteins from the Golgi complex to the cell surface (Table 5).

**Table 5:** Hub genes orchestrating hepatic development in neonatal chickens (D0-D7): functional characterization.

Gene symbol	Full name	Functions
*KIF11*	Kinesin family member 11	*KIF11* is a motor protein that ‌converts‌ chemical energy (typically through ATP hydrolysis) into mechanical force. ‌This protein‌ acts upon microtubules to transport vesicles and organelles from the cell center to the periphery and drive the beating of flagella and cilia. In non-mitotic cells, *KIF11* ‌is required‌ for transporting secretory proteins from the Golgi complex to the cell surface.
*CCNA2*	Cyclin A2	The protein encoded by *CCNA2* belongs to the ‌highly conserved cyclin family‌, whose members function as ‌essential regulators of cell cycle progression‌. ‌This cyclin‌ binds and activates cyclin-dependent kinase 2 (CDK2), ‌thereby driving progression through the G1/S and G2/M phase transitions‌.
*CDC45*	Cell division cycle 45	The *CDC45*-encoded protein is an essential DNA replication initiation factor. It belongs to a conserved complex containing Cdc6/Cdc18, minichromosome maintenance factors (MCMs), and DNA polymerase, crucial for eukaryotic replication initiation. CDC45 interacts with MCM7 and DNA polymerase α, facilitating Polα chromatin loading—a role pivotal in DNA replication.
*MCM6*	Minichromosome Maintenance complex component 6	The *MCM6*-encoded protein, a highly conserved minichromosome maintenance factor essential for eukaryotic DNA replication initiation, forms the hexameric MCM complex—a core pre-replication complex component. This complex enables replication fork formation and recruits replication machinery. The MCM2/4/6/7 subcomplex exhibits DNA helicase activity. CDC2 kinase-mediated phosphorylation reduces helicase function, indicating replication regulation.
*MCM5*	Minichromosome maintenance complex component 5	*MCM5* encodes a protein structurally homologous to S. cerevisiae CDC46, a DNA replication initiation factor. This chromatin-binding MCM family member interacts with ≥2 other members. Upregulated during G0-G1/S transition, it likely regulates cell cycle progression.
*MCM2*	Minichromosome maintenance complex component 2	*MCM2* encodes a highly conserved protein essential for eukaryotic DNA replication initiation. Its hexameric complex is a core pre-replication component that enables replication fork formation and recruits replication machinery. MCM2 forms a regulatory complex with MCM4/6/7 that controls helicase activity. This protein is regulated via phosphorylation by CDC2 and CDC7 kinases.

## Discussion

The first week post-hatch represents a critical phase in broiler liver development (Gao et al., 2018), characterized by dramatic morphological and functional maturation. Our findings demonstrate that between D0 and D7, the liver undergoes substantial growth—evidenced by a 250.69% surge (Fig. 1A) in absolute weight and a 75.85%increase (Fig. 1B) in liver index (*P* < 0.01). Histomorphological examinations further confirm transformative changes in tissue architecture (Fig. 1C). Concurrently, transcriptomic profiling identified 1,762 DEGs, with six downregulated hub genes (*KIF11, CCNA2, CDC45, MCM6, MCM5*, and *MCM2*) emerging as central orchestrators of hepatic maturation. Their coordinated suppression and associated pathways directly underpin the observed phenotypic transitions.

### Hub genes as master orchestrators of liver maturation

The six hub genes (*KIF11, CCNA2, CDC45*, and *MCM2/5/6*) constitute an integrated regulatory network governing both proliferative cessation and functional specialization in postnatal hepatocytes. The *MCM2/5/6* complex acts as the licensing machinery for DNA replication origins (Rankin and Rankin, 2024; Jia and Yu, 2024), with its downregulation at D7 effectively terminating new replication initiation events—a rate-limiting step for exiting hyperplastic growth. *CDC45*'s synergistic decline further destabilizes the CMG (CDC45-MCM-GINS) helicase complex, stalling replication fork progression (Dhingra et al., 2015). Concurrently, *CCNA2* suppression prevents cyclin-dependent kinase activation, arresting cells at G1/S checkpoint (Yang et al., 2023; Atsaves et al., 2014). *KIF11* downregulation disrupts mitotic spindle dynamics, ensuring chromosomal stability during this transitional phase (Cai et al., 2025; Fesquet et al., 2024). This coordinated transcriptional silencing creates a "proliferation brake" that is physiologically indispensable: by day 7, hepatocytes shift from producing daughter cells to expanding cytoplasmic volume (hypertrophy), accommodating increased metabolic organelles like mitochondria and smooth ER.

### Functional coupling between genetic regulation and phenotypic outcomes

Downregulation of *KIF11, CCNA2, CDC45, MCM6, MCM5*, and *MCM2* accompanies both quantitative and qualitative changes in hepatocytes (Li et al., 2024; Bai et al., 2019; Jiang et al., 2023; Blanc et al., 2021; Li and Yang, 2025). These include a marked increase in cell number during an early proliferative phase (prior to D7), pronounced cellular enlargement, and stronger cytoplasmic and nuclear staining—features that suggest metabolic specialization and functional maturation rather than unrestrained growth. Take the downregulation of *CCNA2* and *KIF11* as an example. These two genes converge on key pathways linked to liver and hepatocyte development, such as core metabolic processes, bile secretion, and steroid hormone biosynthesis (Li and Yang, 2025). As replication-related gene expression declines, the hepatic architecture becomes progressively organized. Lobular structures, initially indistinct, take on clear delineation, while central veins and sinusoids mature into well‑developed vascular networks. This shift likely reflects a reallocation of energy from proliferation toward structural refinement. A gradual rise in connective tissue components within the interstitium supports tissue integrity and vascular maturation during the postnatal period, mirroring the broader transition. Together, downregulation of these genes acts as a molecular switch that terminates the hyper‑proliferative state and steers the liver toward structural specialization and functional efficiency by D7. The sequential morphological events—cellular proliferation, architectural organization, and interstitial reinforcement—collectively underpin hepatic development.

### Pathway-level coordination of liver maturation

Functional enrichment analysis delineates a sophisticated regulatory network wherein hub genes synchronize hepatic maturation through three interconnected pathways. First, the suppression of *CCNA2* and *KIF11* orchestrates cell cycle exit by attenuating mitotic activity, thereby enabling hepatocytes to transition from proliferative hyperplasia to cellular hypertrophy—a pivotal driver of the documented 75.85% liver mass expansion (Ozawa et al., 2011; Zeng et al., 2019). Concurrently, the downregulation of MCM replicative helicase complex induces genomic stabilization through termination of DNA synthesis initiation, which not only safeguards genetic integrity but also promotes tissue consolidation by arresting hyperplastic growth (Przanowska et al., 2025; Maher et al., 2011). Notably, pathway interrogation reveals that *CCNA2* depletion concurrently activates cellular senescence programs, serving dual purposes: (1) eliminating transient progenitor populations to optimize lobular microarchitecture, and (2) reallocating metabolic resources towards functional maturation (Min et al., 2020; Zhou et al., 2025). This triphasic regulation—spanning cell cycle control, replication fidelity, and progenitor pool management—collectively enables hepatocytes to redirect biosynthetic capacity towards organelle development and metabolic enzyme production. The resultant phenotypic adaptation, characterized by enhanced lipid processing efficiency and glycogen storage density, ultimately fulfills the stringent energetic demands of post-hatch development.

From a production perspective, the pronounced enrichment of fatty acid metabolism pathways among DEGs offers actionable targets for nutritional intervention. Given that hepatic maturation involves substantial shifts in lipid processing capacity, tailored nutritional strategies—such as optimizing the ratio of unsaturated to saturated fatty acids or supplementing with lipid‑modulating functional additives (e.g., bile acids, lipotropic factors) during the early post‑hatch period (Liu et al., 2024; Wang et al., 2024; Rezaeipour et al., 2019)—may synergistically support the transcriptomic reprogramming identified here. Such approaches could further enhance liver functional reserve and metabolic efficiency, ultimately improving growth performance and flock uniformity in broiler production systems.

Despite the integrated histomorphological and transcriptomic insights provided by this study, several limitations should be acknowledged. First, the investigation focused exclusively on two discrete developmental time points (D0 and D7), which, while capturing the initiation and culmination of the early postnatal phase, do not fully delineate the continuous and dynamic nature of hepatic maturation. The transitional events—such as the precise timing of cell cycle exit, the onset of hypertrophy, and the coordination of metabolic reprogramming—likely involve intermediate stages that remain uncharacterized. Future studies incorporating additional temporal nodes (e.g., D3, D5, D14, and D21) would enable a more detailed resolution of the regulatory trajectories governing liver development. Second, the transcriptomic analysis was performed on pooled samples, which, although effective for reducing biological noise and obtaining robust expression profiles, precludes the assessment of inter-individual variability. Complementary approaches, such as single-cell RNA sequencing or spatially resolved transcriptomics, could further elucidate the cellular heterogeneity and zonal specialization underlying the observed morphological and functional transitions.

## Conclusion and implications

This study establishes that coordinated downregulation of *KIF11, CCNA2, CDC45*, and *MCM2/5/6* orchestrates the neonatal liver's shift from hyperplastic growth to hypertrophic differentiation. Their roles in cell cycle arrest, DNA replication termination, and senescence induction provide a unified mechanistic framework for understanding the organ's morphological maturation (mass gain, index elevation, histological refinement) and metabolic activation. Targeting these hub genes or their upstream regulators could offer novel strategies for optimizing hepatic development—enhancing nutrient utilization, disease resilience, and overall productivity in poultry systems. Future work should validate their temporal expression patterns and explore epigenetic modifiers controlling this developmental switch.

## Author contribution

B Yang and K Fan conceived and designed the study, provided overall research supervision, and were responsible for extensive data analysis and validation. XF Li and AME Abdel-Moneim systematically organized and formatted all tabular data, and led the manuscript drafting with substantial intellectual input. All co‑authors contributed to intellectual discussions, participated in revising the manuscript, and approved the final version for publication.

## Funding

The authors acknowledge their respective universities and institutes for their constant support and help. This study was supported by The Open Project of National Key Laboratory for Tea Plant Germplasm Innovation and Resource Utilization (No. SKLTOF20230121), The Open Project of State Key Laboratory of Animal Biotech Breeding (No. 2024SKLAB6-10), and The Open Project of Longyan University & Fujian Provincial Key Laboratory for Prevention and Control of Animal Infectious Diseases and Biotechnology (No. ZDSYS2023003; No. ZDSYS2025003).

## Data availability

The data presented in this study are available at the present form of the manuscript and the supplementary materials.

## Clinical trial number

Not applicable.

## Ethical compliance

All animal experiments strictly complied with the NIH Guide for the Care and Use of Laboratory Animals and received formal approval from Anhui Science and Technology University's Institutional Animal Care and Use Committee (Protocol EASTU-202410).

## Declaration of AI usage

This research was conducted without utilizing any artificial intelligence-assisted tools for data processing, visual representation creation (including figures and tables), or literature citation management. All analytical procedures and scholarly attributions were performed manually by the human researchers involved in this project. The manuscript represents the authors' authentic intellectual contribution, with all referenced sources appropriately acknowledged in accordance with academic integrity standards.

## CRediT authorship contribution statement

**Xiaofeng Li:** Writing – review & editing, Software. **Abdel-Moneim Eid Abdel-Moneim:** Investigation, Formal analysis, Data curation. **Kewei Fan:** Data curation, Conceptualization. **Bing Yang:** Writing – review & editing, Writing – original draft, Visualization, Validation, Supervision, Software, Project administration, Funding acquisition, Data curation, Conceptualization.

## Disclosures

None.

## Appendix Supplementary materials

Image, application 1

## Footnotes

Supplementary material associated with this article can be found, in the online version, at doi:10.1016/j.psj.2026.106938.
