# Supplementary material for: Decoding the hub gene blueprint for broiler liver development: A synergistic histomorphological and transcriptomic investigation during the neonatal phase
Source: Poult Sci. 2026 Apr 15;105(7):106938. doi: 10.1016/j.psj.2026.106938 (PMC13141760; doi:10.1016/j.psj.2026.106938)
Supplement: Supplementary file 1 [file mmc1.pdf]

**Supplementary file 1.** All DEGs found in the liver of broilers			
**Gene symbol**	**logFC**	**P Value**	**Gene Title**
FADS2	8.53	5.58E-04	Fatty acid desaturase 2
SCD	8.22	3.93E-05	Stearoyl-CoA desaturase (delta-9-desaturase)
THRSP	8.16	5.32E-08	Thyroid hormone responsive
CDKN2B	7.53	2.70E-04	Cyclin-dependent kinase inhibitor 2B (p15, inhibits CDK4)
IGF1	7.10	9.04E-07	Insulin like growth factor 1
FADS1	6.62	7.49E-07	Fatty acid desaturase 1
SQLE	6.33	9.79E-08	Squalene epoxidase
PIGR	5.55	2.36E-03	Polymeric immunoglobulin receptor
AGMO	5.38	2.47E-05	Alkylglycerol monooxygenase
DHCR24	5.27	1.57E-06	24-dehydrocholesterol reductase
TBC1D8	5.22	3.45E-06	TBC1 domain family, member 8 (with GRAM domain)
OCX36	5.20	1.39E-04	BPI fold containing family B, member 3
CDO1	5.15	4.33E-05	Cysteine dioxygenase type 1
ELOVL2	5.08	2.52E-06	ELOVL fatty acid elongase 2
JCHAIN	4.97	2.32E-04	Joining chain of multimeric IgA and IgM
BHLHE41	4.87	8.44E-07	Basic helix-loop-helix family member e41
LSS	4.79	1.04E-06	Lanosterol synthase (2,3-oxidosqualene-lanosterol cyclase)
ACE2	4.69	1.85E-03	Angiotensin I converting enzyme 2
FGF23	4.57	1.57E-03	Fibroblast growth factor 23
KIAA1958	4.57	9.34E-05	KIAA1958
GIF	4.48	1.15E-06	Gastric intrinsic factor
NSDHL	4.43	6.21E-07	NAD(P) dependent steroid dehydrogenase-like
GSTA4	4.43	4.85E-06	Glutathione S-transferase alpha 4
IFI27L2	4.32	1.37E-06	Interferon, alpha-inducible protein 27-like 2
BF2	4.25	1.17E-03	Major histocompatibility complex class I antigen BF2
SLC14A2	4.19	1.27E-03	Solute carrier family 14 (urea transporter), member 2
AFP	4.19	5.81E-03	Alpha-fetoprotein
FDFT1	4.14	6.30E-06	Farnesyl-diphosphate farnesyltransferase 1
MAOB	4.10	6.10E-03	Monoamine oxidase B
CP	4.10	1.04E-04	Ceruloplasmin (ferroxidase)
MMP7	4.10	2.83E-02	Matrix metallopeptidase 7 (matrilysin, uterine)
TBC1D32	4.08	6.63E-03	TBC1 domain family member 32
KCNT2	4.08	1.14E-04	Potassium channel, subfamily T, member 2
HSD17B7	3.99	1.16E-06	Hydroxysteroid (17-beta) dehydrogenase 7
WFDC2	3.95	5.62E-05	WAP four-disulfide core domain 2
CST7	3.94	2.80E-04	Cystatin F
S100Z	3.92	6.36E-03	S100 calcium binding protein Z
MLLT10	3.88	8.94E-06	Myeloid/lymphoid or mixed-lineage leukemia; translocated to, 10
SPATA1	3.85	3.77E-03	Spermatogenesis associated 1
KCNC2	3.83	1.69E-03	Potassium voltage-gated channel, Shaw-related subfamily, member 2
CD3E	3.80	3.50E-05	CD3e molecule
FAM173A	3.80	2.01E-04	Family with sequence similarity 173 member A
ADAMTS18	3.79	3.03E-03	ADAM metallopeptidase with thrombospondin type 1 motif, 18
MUC	3.78	1.72E-02	Mucin protein
MYOZ3	3.77	1.79E-03	Myozenin 3
FDPS	3.75	6.48E-06	Farnesyl diphosphate synthase
BFSP1	3.72	1.62E-02	Beaded filament structural protein 1
PAX5	3.72	1.42E-03	Paired box 5
ENPEP	3.71	1.34E-05	Glutamyl aminopeptidase
PPEF2	3.71	1.57E-02	Protein phosphatase, EF-hand calcium binding domain 2
DHCR7	3.66	3.93E-06	7-dehydrocholesterol reductase
PLIN2	3.65	5.36E-04	Perilipin 2
ENDOUL	3.63	1.30E-03	Endonuclease, polyU-specific-like

MSMO1	3.62	2.72E-06	Methylsterol monooxygenase 1
STARD13	3.58	5.14E-03	StAR-related lipid transfer (START) domain containing 13
PLCH1	3.56	5.10E-03	Phospholipase C, eta 1
DMBT1L	3.55	1.45E-02	Deleted in malignant brain tumors 1 protein-like
PNPLA3	3.55	1.51E-05	Patatin-like phospholipase domain containing 3
NTSR1	3.53	1.05E-05	Neurotensin receptor 1 (high affinity)
IGLL1	3.52	4.44E-03	Immunoglobulin lambda-like polypeptide 1
INSIG1	3.51	1.35E-05	Insulin induced gene 1
TRPM1	3.49	4.64E-04	Transient receptor potential cation channel, subfamily M, member 1
ATCAY	3.49	2.27E-05	Ataxia, cerebellar, Cayman type
SEMA3E	3.47	1.12E-02	Sema domain, immunoglobulin domain (Ig), short basic domain, secreted
ACACA	3.46	1.26E-05	Acetyl-CoA carboxylase alpha
FAT2	3.46	1.52E-05	FAT atypical cadherin 2
ME1	3.44	1.58E-05	Malic enzyme 1, NADP(+)-dependent, cytosolic
GABBR2	3.44	6.22E-03	Gamma-aminobutyric acid type B receptor subunit 2
CHIA	3.44	4.98E-04	Chitinase, acidic
C5H14ORF79	3.39	1.34E-03	Chromosome 5 open reading frame, human C14orf79
CLCN6	3.36	7.29E-03	Chloride voltage-gated channel 6
IDI1	3.36	1.76E-06	Isopentenyl-diphosphate delta isomerase 1
WDFY4	3.34	3.39E-02	WDFY family member 4
ANKS1A	3.34	4.93E-04	Ankyrin repeat and sterile alpha motif domain containing 1A
ACTBL2	3.34	7.76E-03	Actin, beta-like 2
RCN2	3.33	2.19E-03	Reticulocalbin 2
LPXN	3.32	3.26E-04	Leupaxin
ARAP2	3.31	4.07E-02	ArfGAP with RhoGAP domain, ankyrin repeat and PH domain 2
ATG7	3.29	6.04E-04	Autophagy related 7
TNPO1	3.28	3.23E-03	Transportin 1
TNFSF13B	3.27	9.24E-05	Tumor necrosis factor (ligand) superfamily, member 13b
CSGALNACT1	3.27	3.28E-02	Chondroitin sulfate N-acetylgalactosaminyltransferase 1
AGR3	3.25	2.60E-03	Anterior gradient 3
NUDT9	3.25	5.16E-04	Nudix hydrolase 9
ELOVL6	3.25	2.59E-06	ELOVL fatty acid elongase 6
NRCAM	3.24	1.38E-03	Neuronal cell adhesion molecule
FAM3D	3.23	2.15E-03	Family with sequence similarity 3, member D
KIF5C	3.22	1.91E-03	Kinesin family member 5C
CHRNA8	3.22	2.73E-05	Neuronal acetylcholine receptor subunit alpha-7-like
SYNGR3	3.19	5.06E-03	Synaptogyrin 3
MAP1B	3.18	1.27E-02	Microtubule-associated protein 1B
TANC2	3.18	3.84E-04	Tetratricopeptide repeat, ankyrin repeat and coiled-coil containing 2
NFAT5	3.18	6.48E-04	Nuclear factor of activated T-cells 5, tonicity-responsive
C16ORF59	3.17	1.17E-02	Chromosome 14 open reading frame, human C16orf59
RAB19	3.16	1.18E-04	RAB19, member RAS oncogene family
FHL5	3.15	2.07E-04	Four and a half LIM domains 5
ALPL	3.12	2.97E-03	Alkaline phosphatase, liver/bone/kidney
UCHL1	3.12	5.34E-05	Ubiquitin carboxyl-terminal esterase L1 (ubiquitin thiolesterase)
MACF1	3.12	4.62E-04	Microtubule-actin crosslinking factor 1
GDPD4	3.09	1.68E-04	Glycerophosphodiester phosphodiesterase domain containing 4
KCNH7	3.09	1.06E-03	Potassium voltage-gated channel, subfamily H (eag-related), member 7
TRIM3	3.06	1.39E-04	Tripartite motif containing 3
CRHBP	3.04	7.11E-03	Corticotropin releasing hormone binding protein
BLB1	3.04	1.08E-05	Major histocompatibility complex class II beta chain BLB1
SLC22A7	3.04	5.49E-03	Solute carrier family 22 (organic anion transporter), member 7
ZEB2	3.01	1.52E-03	Zinc finger E-box binding homeobox 2
ETFA	3.01	1.83E-03	Electron-transfer-flavoprotein, alpha polypeptide
TSPO2	3.01	4.20E-03	Translocator protein 2
ANKRD9	2.99	1.27E-04	Ankyrin repeat domain 9

EFCAB1	2.99	1.82E-02	EF-hand calcium binding domain 1
USP10	2.98	5.60E-04	Ubiquitin specific peptidase 10
INSRR	2.97	4.01E-02	Insulin receptor-related receptor
SLC7A11	2.97	7.98E-03	Solute carrier family 7, member 11
PPP1R3C	2.96	2.55E-03	Protein phosphatase 1, regulatory subunit 3C
NTNG1	2.96	4.30E-02	Netrin G1
DOCK8	2.95	1.78E-04	Dedicator of cytokinesis 8
STK32B	2.94	3.67E-03	Serine/threonine kinase 32B
SLC24A4	2.93	9.47E-03	Solute carrier family 24 (sodium/potassium/calcium exchanger), member [illegible]
EVL	2.93	1.80E-02	Enah/Vasp-like
SCN8A	2.92	2.20E-02	Sodium channel, voltage gated, type VIII, alpha subunit
DSCAML1	2.92	8.76E-05	Down syndrome cell adhesion molecule like 1
CD2	2.91	4.24E-02	CD2 molecule
STARD5	2.91	1.70E-02	StAR related lipid transfer domain containing 5
G0S2	2.90	2.42E-04	G0/G1 switch 2
EPM2A	2.89	4.89E-03	Epilepsy, progressive myoclonus type 2A, Lafora disease (laforin)
STARD4	2.89	6.71E-06	StAR-related lipid transfer (START) domain containing 4
TMEM260	2.89	7.10E-03	Transmembrane protein 260
GAB3	2.88	4.67E-04	GRB2 associated binding protein 3
HMGCR	2.88	3.17E-06	3-hydroxy-3-methylglutaryl-CoA reductase
C1H21ORF62	2.88	1.67E-03	Chromosome 1 open reading frame, human C21orf62
BAI3	2.88	1.27E-02	Brain-specific angiogenesis inhibitor 3
HCRTR2	2.86	9.87E-03	Hypocretin (orexin) receptor 2
KBTBD3	2.86	1.99E-03	Kelch repeat and BTB domain containing 3
SLC46A3	2.86	1.19E-02	Solute carrier family 46 member 3
RUNDC3A	2.86	1.08E-02	RUN domain containing 3A
GSTA	2.85	1.06E-05	Glutathione S-transferase class-alpha
NAV3	2.85	1.13E-04	Neuron navigator 3
TMEM174	2.85	1.40E-03	Transmembrane protein 174
TEX12	2.85	1.89E-02	Testis expressed 12
PLXNA4B	2.84	1.29E-03	Plexin A4, B
ROS1	2.84	8.15E-03	ROS proto-oncogene 1 , receptor tyrosine kinase
UNC119	2.84	3.62E-03	Unc-119 homolog (C. Elegans)
MND1	2.84	9.84E-04	Meiotic nuclear divisions 1
CRP	2.84	6.55E-03	C-reactive protein, pentraxin-related
ART4	2.82	2.70E-02	ADP-ribosyltransferase 4 (Dombrock blood group)
CYP2J6L3	2.82	4.91E-02	Cytochrome P450 2J6-like 3
DLGAP1	2.82	4.38E-03	Discs, large (Drosophila) homolog-associated protein 1
CD74	2.81	1.03E-05	CD74 molecule
USF3	2.81	1.05E-02	Upstream transcription factor family member 3
AACS	2.81	2.30E-05	Acetoacetyl-CoA synthetase
SATB1	2.81	3.64E-03	SATB homeobox 1
PAN3	2.80	1.22E-02	PAN3 poly(A) specific ribonuclease subunit
BAIAP2L2	2.80	8.69E-05	BAI1 associated protein 2 like 2
XDH	2.79	3.34E-03	Xanthine dehydrogenase
RIMS2	2.78	1.82E-02	Regulating synaptic membrane exocytosis 2
DIDO1	2.78	3.05E-03	Death inducer-obliterator 1
CTC1	2.77	4.84E-04	CTS telomere maintenance complex component 1
IL2RA	2.77	3.15E-02	Interleukin 2 receptor, alpha
CD83	2.77	2.10E-02	CD83 molecule
LDLR	2.77	6.95E-05	Low density lipoprotein receptor
BPIFB4	2.76	3.46E-03	BPI fold containing family B, member 4
DPP4	2.75	1.02E-04	Dipeptidyl-peptidase 4
NOX1	2.75	3.98E-02	NADPH oxidase 1
CCR7	2.74	1.24E-03	C-C motif chemokine receptor 7
C1H22ORF23	2.74	4.63E-03	Chromosome 1 open reading frame, human C22orf23

ACTR8	2.73	2.25E-02	ARP8 actin-related protein 8 homolog (yeast)
SREBF2	2.73	2.46E-04	Sterol regulatory element binding transcription factor 2
RGS9	2.73	9.10E-04	Regulator of G-protein signaling 9
PRSS35	2.73	7.26E-03	Protease, serine 35
RBKS	2.71	4.44E-03	Ribokinase
SBF2	2.70	3.53E-02	SET binding factor 2
TRPV4	2.70	1.46E-02	Transient receptor potential cation channel, subfamily V, member 4
MICAL3	2.70	4.11E-02	Microtubule associated monooxygenase, calponin and LIM domain contai
FZD5	2.69	8.65E-03	Frizzled class receptor 5
COL4A3	2.68	1.76E-03	Collagen, type IV, alpha 3 (Goodpasture antigen)
CD3D	2.68	7.28E-05	CD3d molecule, delta (CD3-TCR complex)
INPP4B	2.67	5.00E-03	Inositol polyphosphate-4-phosphatase, type II, 105kDa
OVSTL	2.66	3.28E-04	Ovostatin-like
SEBOX	2.65	4.08E-05	SEBOX homeobox
WAC	2.65	3.67E-03	WW domain containing adaptor with coiled-coil
LAMA1	2.65	1.59E-02	Laminin, alpha 1
DCLK3	2.65	1.98E-02	Doublecortin like kinase 3
FAM167B	2.65	8.14E-03	Family with sequence similarity 167, member B
SLC9A4	2.64	5.68E-03	Solute carrier family 9, subfamily A member 4
FLVCR2	2.64	2.25E-02	Feline leukemia virus subgroup C cellular receptor family, member 2
PCSK1	2.64	4.70E-04	Proprotein convertase subtilisin/kexin type 1
RAPSN	2.64	1.99E-02	Receptor associated protein of the synapse
INPP5D	2.63	1.41E-05	Inositol polyphosphate-5-phosphatase, 145kDa
TSGA10	2.63	1.79E-02	Testis specific, 10
UGT1A6	2.62	3.15E-02	UDP glucuronosyltransferase 1 family, polypeptide A6
SDSL	2.62	2.63E-02	Serine dehydratase-like
CAP1	2.62	2.43E-02	CAP, adenylate cyclase-associated protein 1 (yeast)
FASN	2.62	1.55E-04	Fatty acid synthase
USH1C	2.61	2.15E-02	USH1 protein network component harmonin
DUSP15	2.61	2.21E-02	Dual specificity phosphatase 15
CNTN3	2.61	3.20E-03	Contactin 3 (plasmacytoma associated)
STK32A	2.61	7.30E-03	Serine/threonine kinase 32A
C1QTNF8	2.60	3.99E-02	C1q and tumor necrosis factor related protein 8
IL22	2.59	5.49E-04	Interleukin 22
IFT80	2.59	2.66E-05	Intraflagellar transport 80 homolog (Chlamydomonas)
AvBD8	2.59	1.04E-04	Avian beta-defensin 8
TRIO	2.58	3.14E-03	Trio Rho guanine nucleotide exchange factor
VPS13B	2.58	3.21E-02	Vacuolar protein sorting 13 homolog B (yeast)
RNF34	2.58	4.03E-05	Ring finger protein 34
HES6	2.58	1.63E-02	Hairy and enhancer of split 6 (Drosophila)
MGME1	2.57	8.26E-04	Mitochondrial genome maintenance exonuclease 1
K123	2.57	4.49E-04	K123 protein
LRRC40	2.57	2.01E-02	Leucine rich repeat containing 40
TPPP	2.56	3.21E-04	Tubulin polymerization promoting protein
HID1	2.56	2.52E-02	HID1 domain containing
GRID1	2.55	1.59E-02	Glutamate receptor, ionotropic, delta 1
ZNF319	2.55	1.74E-02	Zinc finger protein 319
GJD3	2.55	9.38E-03	Gap junction protein, delta 3, 31.9kDa
MPP3	2.55	2.09E-02	Membrane protein, palmitoylated 3 (MAGUK p55 subfamily member 3)
SIRT6	2.55	1.54E-03	Sirtuin 6
GMEB1	2.55	3.06E-02	Glucocorticoid modulatory element binding protein 1
ACER1	2.55	4.35E-02	Alkaline ceramidase 1
TXNRD1	2.55	3.48E-02	Thioredoxin reductase 1
HOXA11	2.54	2.00E-02	Homeobox A11
CPXM2	2.54	7.65E-04	Carboxypeptidase X (M14 family), member 2
CD1C	2.53	2.49E-02	CD1c molecule

KCNG4	2.53	2.66E-02	Potassium voltage-gated channel, subfamily G, member 4
CCKAR	2.53	7.42E-03	Cholecystokinin A receptor
GRIP2	2.52	2.77E-02	Glutamate receptor interacting protein 2
SMOC1	2.52	1.97E-02	SPARC related modular calcium binding 1
CCDC170	2.51	2.80E-03	Coiled-coil domain containing 170
TNRC6A	2.51	2.43E-03	Trinucleotide repeat containing 6A
BCO2	2.50	2.40E-03	Beta-carotene oxygenase 2
LMO7	2.50	9.31E-03	LIM domain 7
WBP11	2.49	4.75E-02	WW domain binding protein 11
TNNC1	2.49	1.62E-04	Troponin C type 1 (slow)
ASB3	2.49	4.36E-02	Ankyrin repeat and SOCS box-containing 3
SLC6A7	2.49	1.40E-04	Solute carrier family 6 (neurotransmitter transporter), member 7
MAFG	2.49	2.54E-03	V-maf avian musculoaponeurotic fibrosarcoma oncogene homolog G
ELOVL5	2.48	5.62E-05	ELOVL fatty acid elongase 5
DBH	2.47	1.16E-02	Dopamine beta-hydroxylase
LRRC66	2.47	2.74E-02	Leucine rich repeat containing 66
CPN2	2.47	2.29E-02	Carboxypeptidase N, polypeptide 2
CUL4B	2.47	3.83E-02	Cullin 4B
TTN	2.46	4.17E-02	Titin
LMOD3	2.46	6.17E-03	Leiomodin 3 (fetal)
BANK1	2.45	3.02E-03	B-cell scaffold protein with ankyrin repeats 1
TRMT10C	2.45	4.18E-03	TRNA methyltransferase 10C, mitochondrial RNase P subunit
WNT4	2.45	2.83E-03	Wingless-type MMTV integration site family member 4
LYG2	2.44	2.90E-03	Lysozyme G-like 2
SP2	2.44	3.52E-02	Sp2 transcription factor
SH3TC2	2.43	1.82E-02	SH3 domain and tetratricopeptide repeats 2
VGLL3	2.42	2.65E-02	Vestigial like family member 3
SLC22A13L	2.42	1.06E-02	Solute carrier family 22 member 13-like
ANXA13	2.41	4.39E-04	Annexin A13
MTUS2	2.41	1.29E-02	Microtubule associated tumor suppressor candidate 2
USP49	2.41	3.29E-02	Ubiquitin specific peptidase 49
NAAA	2.41	5.10E-05	N-acylethanolamine acid amidase
AK3	2.40	9.31E-06	Adenylate kinase 3
CMTM6	2.40	1.97E-02	CKLF like MARVEL transmembrane domain containing 6
PPIL6	2.39	2.45E-03	Peptidylprolyl isomerase like 6
CMPK2	2.39	4.67E-02	Cytidine/uridine monophosphate kinase 2
CUEDC1	2.39	2.75E-02	CUE domain containing 1
TACSTD2	2.39	4.27E-02	Tumor-associated calcium signal transducer 2
SLC41A2	2.38	4.20E-04	Solute carrier family 41, member 2
FCHSD2	2.38	4.63E-02	FCH and double SH3 domains 2
AMY1AP	2.37	2.40E-02	Hepatic amylase pseudogene
SOCS5	2.37	4.28E-02	Suppressor of cytokine signaling 5
VPS51	2.37	1.88E-02	VPS51, GARP complex subunit
C12H3ORF67	2.36	2.09E-02	Chromosome 12 open reading frame, human C3orf67
GREB1	2.36	4.21E-03	Growth regulation by estrogen in breast cancer 1
IGSF3	2.35	3.24E-02	Immunoglobulin superfamily, member 3
RDH12	2.35	1.14E-05	Retinol dehydrogenase 12 (all-trans/9-cis/11-cis)
CDKN2A	2.35	5.90E-04	Cyclin-dependent kinase inhibitor 2A (melanoma, p16, inhibits CDK4)
DKK1	2.34	2.95E-02	Dickkopf WNT signaling pathway inhibitor 1
EPT1	2.34	2.75E-05	Ethanolaminephosphotransferase 1 (CDP-ethanolamine-specific)
COR6	2.34	8.28E-03	Chick olfactory receptor 6
ALCAM	2.34	3.47E-02	Activated leukocyte cell adhesion molecule
GUCY1B4	2.33	4.90E-02	Guanylate cyclase 1, soluble, beta 4
ST6GALNAC1	2.33	1.14E-02	ST6 (alpha-N-acetyl-neuraminyl-2,3-beta-galactosyl-1,3)-N-acetylgalactos
AXIN1	2.33	2.35E-02	Axin 1
NFAM1	2.32	2.75E-02	NFAT activating protein with ITAM motif 1

ISX	2.32	7.07E-04	Intestine-specific homeobox
CBX7	2.32	3.08E-04	Chromobox homolog 7
LUZP2	2.32	9.12E-03	Leucine zipper protein 2
CLCN1	2.32	3.67E-02	Chloride channel, voltage-sensitive 1
ENPP2	2.32	3.61E-05	Ectonucleotide pyrophosphatase/phosphodiesterase 2
STMN4	2.32	2.37E-02	Stathmin-like 4
C2CD3	2.31	1.14E-03	C2 calcium-dependent domain containing 3
ACSBG2	2.31	2.58E-05	Acyl-CoA synthetase bubblegum family member 2
WIPF3	2.31	2.61E-02	WAS/WASL interacting protein family member 3
NT5C2	2.30	1.17E-02	5'-nucleotidase, cytosolic II
ANGPTL4	2.30	1.31E-04	Angiopoietin like 4
CCDC73	2.30	3.87E-02	Coiled-coil domain containing 73
LYRM4	2.30	1.22E-02	LYR motif containing 4
SLC27A6	2.29	9.46E-03	Solute carrier family 27 (fatty acid transporter), member 6
LRCH2	2.29	3.00E-02	Leucine-rich repeats and calponin homology (CH) domain containing 2
SC5D	2.29	1.34E-04	Sterol-C5-desaturase
ABCG1	2.28	3.40E-02	ATP-binding cassette, sub-family G (WHITE), member 1
DDX60	2.28	1.51E-02	DEAD (Asp-Glu-Ala-Asp) box polypeptide 60
GUCA2B	2.27	1.94E-04	Guanylate cyclase activator 2B (uroguanylin)
ANGPTL3	2.27	1.55E-03	Angiopoietin like 3
HIST1H2AH	2.27	3.44E-02	Histone cluster 1, H2ah
GTF2IRD1	2.27	1.87E-02	GTF2I repeat domain containing 1
RBM15B	2.26	9.34E-03	RNA binding motif protein 15B
KCNK17	2.26	5.11E-03	Potassium channel, subfamily K, member 17
HPS3	2.26	6.28E-04	HPS3, biogenesis of lysosomal organelles complex 2 subunit 1
GHRH	2.26	3.94E-02	Growth hormone releasing hormone
PANK3	2.26	4.82E-05	Pantothenate kinase 3
FBXO21	2.25	1.40E-02	F-box protein 21
C28H19ORF35	2.25	4.10E-02	Chromosome 28 open reading frame, human C19orf35
ARHGEF1	2.25	7.11E-03	Rho guanine nucleotide exchange factor (GEF) 1
ATG14	2.25	1.21E-02	Autophagy related 14
LRRC72	2.24	2.93E-02	Leucine rich repeat containing 72
BAK1	2.23	1.59E-03	BCL2-antagonist/killer 1
RADIL	2.23	1.18E-02	Ras associating with DIL domains
TMEM179	2.23	4.21E-03	Transmembrane protein 179
PLPP7	2.23	1.48E-02	Phospholipid phosphatase 7 (inactive)
MVD	2.22	5.79E-04	Mevalonate diphosphate decarboxylase
PCDH17	2.22	2.33E-02	Protocadherin 17
PLCB2	2.22	5.49E-04	Phospholipase C, beta 2
ENKUR	2.21	1.41E-02	Enkurin, TRPC channel interacting protein
ERCC6L2	2.21	1.04E-02	Excision repair cross-complementation group 6 like 2
CARD11	2.21	2.61E-02	Caspase recruitment domain family, member 11
TMEM243	2.20	6.67E-04	Transmembrane protein 243
IRG1	2.20	4.76E-04	Immunoresponsive 1 homolog (mouse)
HSF5	2.20	1.02E-02	Heat shock transcription factor family member 5
DNASE1	2.19	3.32E-02	Deoxyribonuclease I
DRAM1	2.19	8.15E-03	DNA damage regulated autophagy modulator 1
LRRC10	2.19	4.80E-03	Leucine rich repeat containing 10
UCP3	2.19	3.93E-02	Uncoupling protein 3 (mitochondrial, proton carrier)
EFCAB3	2.19	3.69E-02	EF-hand calcium binding domain 3
TFF2	2.18	3.32E-03	Trefoil factor 2
FBXL2	2.18	4.22E-03	F-box and leucine-rich repeat protein 2
TLE3	2.17	1.59E-02	Transducin-like enhancer of split 3 (E(sp1) homolog, Drosophila)
TUBB2A	2.17	2.17E-02	Tubulin, beta 2A class IIa
BEGAIN	2.17	5.74E-03	Brain enriched guanylate kinase associated
EFNA5	2.16	2.03E-02	Ephrin-A5

GNLY	2.16	6.87E-05	Granulysin
KL	2.16	3.52E-02	Klotho
ICOS	2.15	4.15E-04	Inducible T-cell co-stimulator
TLR21	2.15	2.16E-02	Toll-like receptor 21
GFM2	2.15	6.74E-03	G elongation factor, mitochondrial 2
CYP1A2	2.15	2.09E-02	Cytochrome P450, family 1, subfamily A, polypeptide 2
TRPV6	2.15	1.17E-02	Transient receptor potential cation channel, subfamily V, member 6
VH1	2.14	3.71E-02	Uncharacterized LOC431191
PRRG4	2.14	8.76E-03	Proline rich Gla (G-carboxyglutamic acid) 4 (transmembrane)
AIM1L	2.13	1.65E-02	Absent in melanoma 1-like
DNAH7	2.13	2.50E-04	Dynein, axonemal, heavy chain 7
FAM109B	2.13	1.50E-02	Family with sequence similarity 109, member B
LRRC3B	2.13	3.01E-03	Leucine rich repeat containing 3B
RNF213	2.13	2.71E-03	Ring finger protein 213
HYAL3	2.11	4.10E-02	Hyaluronoglucosaminidase 3
MTMR9LP	2.11	2.80E-02	Myotubularin related protein 9-like, pseudogene
SOGA1	2.11	4.56E-03	Suppressor of glucose, autophagy associated 1
ZBTB25	2.11	1.58E-02	Zinc finger and BTB domain containing 25
SV2C	2.11	8.65E-03	Synaptic vesicle glycoprotein 2C
ACHE	2.11	1.27E-02	Acetylcholinesterase (Yt blood group)
PDE4C	2.10	6.60E-03	Phosphodiesterase 4C
SLC9A2	2.10	2.06E-02	Solute carrier family 9, subfamily A (NHE2, cation proton antiporter 2), m
ATP2A1	2.10	1.25E-02	ATPase, Ca++ transporting, cardiac muscle, fast twitch 1
CCR9	2.09	4.70E-02	Chemokine (C-C motif) receptor 9
CDH20	2.09	4.29E-02	Cadherin 20, type 2
C3AR1	2.09	4.10E-04	Complement component 3a receptor 1
DOCK2	2.09	2.44E-04	Dedicator of cytokinesis 2
TTPA	2.09	5.19E-05	Tocopherol (alpha) transfer protein
P2RX7	2.09	4.46E-03	Purinergic receptor P2X 7
MAPRE2	2.09	4.61E-03	Microtubule-associated protein, RP/EB family, member 2
ARHGAP1	2.08	1.33E-02	Rho GTPase activating protein 1
SLC22A2	2.08	4.14E-02	Solute carrier family 22 (organic cation transporter), member 2
GZMA	2.08	1.18E-03	Granzyme A (granzyme 1, cytotoxic T-lymphocyte-associated serine ester
VIT	2.07	3.45E-02	Vitrin
RGS5	2.06	1.61E-04	Regulator of G-protein signaling 5
SMIM3	2.06	1.58E-04	Small integral membrane protein 3
HOXA7	2.05	9.72E-03	Homeobox A7
RGS10	2.05	3.52E-02	Regulator of G-protein signaling 10
VPS54	2.04	1.56E-02	VPS54, GARP complex subunit
EIF4G3	2.04	4.84E-02	Eukaryotic translation initiation factor 4 gamma, 3
ADH1C	2.03	6.83E-05	Alcohol dehydrogenase 1C (class I), gamma polypeptide
NHLH2	2.02	2.76E-02	Nescient helix loop helix 2
S100A10	2.02	1.31E-04	S100 calcium binding protein A10
CRISP2	2.02	7.82E-03	Cysteine-rich secretory protein 2
FGL2	2.02	9.35E-03	Fibrinogen like 2
SUFU	2.02	3.26E-02	SUFU negative regulator of hedgehog signaling
CRD_FZ5	2.01	2.88E-03	Unnamed protein product
KCNMA1	2.00	4.97E-05	Potassium large conductance calcium-activated channel, subfamily M, alp
SIM1	2.00	9.96E-04	Single-minded family bHLH transcription factor 1
GPX3	2.00	4.68E-05	Glutathione peroxidase 3
BRE	2.00	7.00E-03	Brain and reproductive organ-expressed (TNFRSF1A modulator)
TACR2	1.99	4.62E-02	Tachykinin receptor 2
DEF6	1.99	3.26E-02	Differentially expressed in FDCP 6 homolog (mouse)
SLCO1A2	1.99	7.10E-05	Solute carrier organic anion transporter family, member 1A2
IL4I1	1.98	7.07E-04	Interleukin 4 induced 1
LCK	1.98	2.87E-02	Lymphocyte-specific protein tyrosine kinase

GBX2	1.98	2.36E-02	Gastrulation brain homeobox 2
CCDC42B	1.97	1.39E-03	Coiled-coil domain containing 42B
CCNE1	1.97	3.68E-03	Cyclin E1
SMUG1	1.96	1.44E-02	Single-strand-selective monofunctional uracil-DNA glycosylase 1
CLIP2	1.96	4.45E-02	CAP-GLY domain containing linker protein 2
SPIRE2	1.96	5.26E-03	Spire-type actin nucleation factor 2
VILL	1.96	5.23E-03	Villin-like
DNLZ	1.95	1.87E-02	DNL-type zinc finger
MAP7D3	1.95	1.91E-02	MAP7 domain containing 3
BCMO1	1.95	1.09E-04	Beta-carotene 15,15'-monooxygenase 1
ENO3	1.95	2.16E-02	Enolase 3 (beta, muscle)
ASS1	1.95	4.80E-04	Argininosuccinate synthase 1
ART1	1.95	2.93E-02	ADP-ribosyltransferase 1
MYL1	1.95	3.20E-02	Myosin, light chain 1, alkali; skeletal, fast
DHRS12	1.94	2.64E-02	Dehydrogenase/reductase (SDR family) member 12
LRRC59	1.94	2.13E-02	Leucine rich repeat containing 59
MFAP2	1.94	1.93E-02	Microfibrillar associated protein 2
NUMA1	1.94	4.45E-02	Nuclear mitotic apparatus protein 1
TARP	1.94	2.98E-02	TCR gamma alternate reading frame protein
WNT11B	1.94	1.46E-02	Wingless-type MMTV integration site family, member 11b
AVD	1.93	3.21E-02	Avidin
NOL6	1.93	2.56E-02	Nucleolar protein 6
TMEFF1	1.93	2.16E-02	Transmembrane protein with EGF-like and two follistatin-like domains 1
NEUROD4	1.93	3.67E-02	Neuronal differentiation 4
TRIM55	1.93	2.47E-02	Tripartite motif containing 55
SLCO1C1	1.93	4.35E-02	Solute carrier organic anion transporter family, member 1C1
FAM192A	1.92	3.19E-02	Family with sequence similarity 192 member A
MAP3K7CL	1.92	3.50E-03	MAP3K7 C-terminal like
SPAG4	1.91	4.44E-02	Sperm associated antigen 4
TRANK1	1.91	2.25E-04	Tetratricopeptide repeat and ankyrin repeat containing 1
TAP2	1.91	4.45E-05	Transporter 2, ATP-binding cassette, sub-family B (MDR/TAP)
SERPINA12	1.91	6.07E-03	Serpin peptidase inhibitor, clade A (alpha-1 antiproteinase, antitrypsin), m
ARAP3	1.91	6.27E-03	ArfGAP with RhoGAP domain, ankyrin repeat and PH domain 3
C9H2ORF72	1.91	2.89E-03	Chromosome 9 open reading frame, human C2orf72
GRM1	1.90	9.08E-04	Glutamate receptor, metabotropic 1
RAB11FIP1	1.90	3.68E-02	RAB11 family interacting protein 1 (class I)
SOX11	1.89	1.01E-02	SRY-box 11
SCN4A	1.89	1.40E-02	Sodium channel, voltage-gated, type IV, alpha subunit
XCL1	1.89	2.82E-04	Lymphotactin
OvoDA1	1.89	3.65E-02	Ovodefensin A1
CYP11A1	1.89	2.12E-04	Cytochrome P450 family 11 subfamily A member 1
FER1L6	1.88	1.81E-03	Fer-1-like 6 (C. Elegans)
MED27	1.88	4.13E-03	Mediator complex subunit 27
NPY2R	1.88	4.41E-03	Neuropeptide Y receptor Y2
ST8SIA6	1.88	3.10E-02	ST8 alpha-N-acetyl-neuraminide alpha-2,8-sialyltransferase 6
PTPRC	1.87	7.85E-05	Protein tyrosine phosphatase, receptor type C
EAF2	1.87	1.42E-04	ELL associated factor 2
FGD4	1.86	9.45E-03	FYVE, RhoGEF and PH domain containing 4
HNMT	1.86	8.45E-05	Histamine N-methyltransferase
TENM3	1.86	2.05E-02	Teneurin transmembrane protein 3
TBX20	1.86	4.03E-02	T-box 20
CUTA	1.85	9.50E-04	CutA divalent cation tolerance homolog (E. Coli)
ZIC4	1.85	3.44E-02	Zic family member 4
UHRF1BP1	1.84	3.25E-02	UHRF1 binding protein 1
KIF21B	1.84	4.18E-02	Kinesin family member 21B
DOK7	1.84	4.52E-03	Docking protein 7

VCPKMT	1.84	2.74E-03	Valosin containing protein lysine methyltransferase
CYP2W2	1.83	1.95E-04	Cytochrome P450, family 2, subfamily W, polypeptide 2
SAMD9L	1.83	3.03E-02	Sterile alpha motif domain containing 9-like
PXDC1	1.83	2.29E-04	PX domain containing 1
SERPINA3	1.82	7.35E-03	Serpin peptidase inhibitor, clade A (alpha-1 antiproteinase, antitrypsin), m
C14H16ORF89	1.82	1.28E-02	Chromosome 14 open reading frame, human C16orf89
HHLA2	1.82	1.23E-03	HERV-H LTR-associating 2
B2M	1.82	4.58E-04	Beta-2-microglobulin
WIF1	1.82	3.95E-02	WNT inhibitory factor 1
SYPL1	1.81	7.84E-04	Synaptophysin-like 1
AGXT2	1.81	9.99E-05	Alanine--glyoxylate aminotransferase 2
GPAM	1.81	1.43E-04	Glycerol-3-phosphate acyltransferase, mitochondrial
SNX22	1.81	1.51E-04	Sorting nexin 22
GPRC5A	1.80	2.93E-04	G protein-coupled receptor, family C, group 5, member A
DIXDC1	1.78	4.35E-02	DIX domain containing 1
HBAD	1.78	1.13E-04	Alpha-D-globin
IL16	1.78	4.87E-03	Interleukin 16
PCDH15	1.78	3.66E-02	Protocadherin-related 15
OSGIN1	1.77	2.88E-03	Oxidative stress induced growth inhibitor 1
CWH43	1.77	2.11E-02	Cell wall biogenesis 43 C-terminal homolog
HKDC1	1.77	6.32E-05	Hexokinase domain containing 1
SNX8	1.76	1.31E-02	Sorting nexin 8
TM6SF1	1.76	6.24E-05	Transmembrane 6 superfamily member 1
CHD6	1.76	2.51E-02	Chromodomain helicase DNA binding protein 6
PRR9	1.75	8.62E-03	Proline rich 9
UMOD	1.75	1.13E-02	Uromodulin
EDIL3	1.75	3.49E-02	EGF-like repeats and discoidin I-like domains 3
CEP170	1.74	1.21E-02	Centrosomal protein 170kDa
CCDC17	1.74	4.95E-02	Coiled-coil domain containing 17
KRT6A	1.74	4.33E-02	Keratin 6A
ZCCHC11	1.74	6.31E-04	Zinc finger, CCHC domain containing 11
A4GALT	1.73	1.38E-02	Alpha 1,4-galactosyltransferase
VPS25	1.72	5.82E-03	Vacuolar protein sorting 25 homolog (S. Cerevisiae)
DMA	1.72	7.94E-04	Major histocompatibility complex, class II, DM alpha
SCNN1G	1.72	1.42E-02	Sodium channel epithelial 1 gamma subunit
CYP4B1L	1.72	7.47E-05	Cytochrome P450 4B1-like
ADCY7	1.71	7.56E-03	Adenylate cyclase 7
LEAP2	1.71	1.72E-04	Liver expressed antimicrobial peptide 2
AFMID	1.71	1.36E-04	Arylformamidase
RASAL2	1.71	2.46E-02	RAS protein activator like 2
RASSF5	1.71	5.32E-03	Ras association domain family member 5
ETNK2	1.71	4.80E-02	Ethanolamine kinase 2
MAPK1IP1L	1.70	3.86E-02	Mitogen-activated protein kinase 1 interacting protein 1-like
ACLY	1.70	3.03E-04	ATP citrate lyase
ATP8B3	1.69	4.29E-02	ATPase, aminophospholipid transporter, class I, type 8B, member 3
SCAPER	1.69	2.70E-02	S-phase cyclin A-associated protein in the ER
HCLS1	1.69	7.06E-04	Hematopoietic cell-specific Lyn substrate 1
GPATCH4	1.69	1.25E-02	G-patch domain containing 4
FEZ1	1.68	4.20E-02	Fasciculation and elongation protein zeta 1
PTGES	1.68	4.23E-02	Prostaglandin E synthase
FAAP100	1.68	4.89E-02	Fanconi anemia core complex associated protein 100
SHROOM1	1.68	4.33E-04	Shroom family member 1
LOXL4	1.68	4.72E-02	Lysyl oxidase-like 4
GLIPR1L	1.67	2.43E-04	GLI pathogenesis-related 1-like
KDM5B	1.67	2.97E-02	Lysine (K)-specific demethylase 5B
CUX1	1.67	4.79E-02	Cut-like homeobox 1

TEKT2	1.67	4.07E-02	Tektin 2
ALDH9A1	1.67	2.52E-04	Aldehyde dehydrogenase 9 family member A1
NRXN3	1.67	3.30E-02	Neurexin 3
CD40LG	1.66	1.95E-02	CD40 ligand
GLE1	1.66	1.43E-02	GLE1, RNA export mediator
PTGS2	1.66	9.18E-05	Prostaglandin-endoperoxide synthase 2 (prostaglandin G/H synthase and c
GPD1	1.66	3.57E-02	Glycerol-3-phosphate dehydrogenase 1 (soluble)
CACNA1G	1.66	1.33E-02	Calcium channel, voltage-dependent, T type, alpha 1G subunit
CD180	1.66	1.15E-03	CD180 molecule
PLA2G10	1.66	1.47E-02	Phospholipase A2, group X
LYN	1.65	1.95E-04	V-yes-1 Yamaguchi sarcoma viral related oncogene homolog
CA6	1.65	4.32E-02	Carbonic anhydrase VI
GPR126	1.65	6.77E-03	G protein-coupled receptor 126
CEP290	1.64	2.31E-02	Centrosomal protein 290kDa
GJA1	1.64	1.98E-04	Gap junction protein, alpha 1, 43kDa
DUSP14	1.64	7.00E-04	Dual specificity phosphatase 14
SARM1	1.63	1.24E-03	Sterile alpha and TIR motif containing 1
GM2A	1.63	7.60E-04	GM2 ganglioside activator
MAP7D2	1.63	4.87E-02	MAP7 domain containing 2
ACMSD	1.62	3.65E-04	Aminocarboxymuconate semialdehyde decarboxylase
C15ORF48	1.62	2.17E-04	Chromosome 10 open reading frame, human C15orf48
ADGB	1.62	4.01E-02	Androglobin
NFYA	1.62	4.41E-03	Nuclear transcription factor Y subunit alpha
FRK	1.61	5.53E-03	Fyn related Src family tyrosine kinase
SLC8A3	1.61	2.24E-02	Solute carrier family 8 (sodium/calcium exchanger), member 3
PLEKHB1	1.61	1.58E-03	Pleckstrin homology domain containing, family B (evectins) member 1
FNBP1	1.60	3.31E-02	Formin binding protein 1
CLASP2	1.60	1.00E-02	Cytoplasmic linker associated protein 2
ZC3H12A	1.60	1.41E-03	Zinc finger CCCH-type containing 12A
MPP7	1.60	4.33E-02	Membrane protein, palmitoylated 7 (MAGUK p55 subfamily member 7)
BARX2	1.60	2.62E-03	BARX homeobox 2
KAT2B	1.59	3.00E-04	K(lysine) acetyltransferase 2B
A2ML4	1.59	4.66E-04	Alpha-2-macroglobulin-like 4
PRTG	1.59	2.86E-02	Protogenin
KIAA1841	1.59	5.82E-03	KIAA1841
TMPRSS9	1.59	4.26E-03	Transmembrane protease, serine 9
MTTPL	1.59	1.33E-03	Microsomal triglyceride transfer protein-like
TRIM14	1.58	7.74E-05	Tripartite motif containing 14
MEF2C	1.58	5.29E-04	Myocyte enhancer factor 2C
BCL2L14	1.58	7.07E-03	BCL2 like 14
KITLG	1.58	4.94E-03	KIT ligand
ELF3	1.57	2.40E-03	E74 like ETS transcription factor 3
CCDC84	1.57	3.76E-02	Coiled-coil domain containing 84
CDH22	1.57	1.45E-02	Cadherin 22, type 2
ST6GALNAC2	1.56	1.08E-02	ST6-N-acetylgalactosaminide alpha-2,6-sialyltransferase 2
CCNG2	1.56	6.73E-04	Cyclin G2
CERS4	1.56	1.91E-02	Ceramide synthase 4
C3H8ORF80	1.55	2.17E-03	Chromosome 3 open reading frame, human C8orf80
RASSF7	1.55	4.94E-02	Ras association (RalGDS/AF-6) domain family (N-terminal) member 7
HTATIP2	1.55	2.54E-03	HIV-1 Tat interactive protein 2
ROBO1	1.55	9.64E-04	Roundabout, axon guidance receptor, homolog 1 (Drosophila)
SYT15	1.55	2.75E-02	Synaptotagmin XV
P2RY6	1.55	5.74E-03	Pyrimidinergic receptor P2Y6
MYL4	1.54	4.56E-04	Myosin, light chain 4, alkali; atrial, embryonic
ITGB2	1.54	9.47E-04	Integrin, beta 2
CALCRL	1.54	1.10E-02	Calcitonin receptor-like

FCER1G	1.54	1.22E-03	Fc fragment of IgE receptor Ig
ISCA1	1.54	2.55E-02	Iron-sulfur cluster assembly 1 homolog
DOHH	1.54	7.70E-03	Deoxyhypusine hydroxylase/monooxygenase
PLAC8L1	1.54	1.54E-02	Placenta-specific 8-like 1
HTR7	1.53	9.52E-03	5-hydroxytryptamine (serotonin) receptor 7, adenylate cyclase-coupled
SERINC3	1.53	5.86E-03	Serine incorporator 3
KCNRG	1.53	3.04E-02	Potassium channel regulator
SH2D1B	1.53	4.43E-02	SH2 domain containing 1B
IGFALS	1.53	1.48E-04	Insulin-like growth factor binding protein, acid labile subunit
GLIPR2	1.53	5.39E-03	GLI pathogenesis-related 2
TIMD4	1.52	1.18E-04	T-cell immunoglobulin and mucin domain containing 4
ODF2	1.52	3.53E-02	Outer dense fiber of sperm tails 2
DPF3	1.51	3.16E-02	D4, zinc and double PHD fingers, family 3
ACAT2	1.51	2.39E-03	Acetyl-CoA acetyltransferase 2
SPTSSB	1.51	2.02E-02	Serine palmitoyltransferase, small subunit B
IRF1	1.51	9.87E-04	Interferon regulatory factor 1
NTF3	1.51	1.12E-02	Neurotrophin 3
MFF	1.51	2.06E-02	Mitochondrial fission factor
ADGRG3	1.50	9.00E-03	Adhesion G protein-coupled receptor G3
ABLIM2	1.50	2.09E-02	Actin binding LIM protein family, member 2
MAL	1.50	2.83E-02	Mal, T-cell differentiation protein
CYP4B1	1.50	5.87E-03	Cytochrome P450, family 4, subfamily B, polypeptide 1
LXN	1.50	1.72E-02	Latexin
HMCN1	1.49	3.36E-02	Hemicentin 1
CDK14	1.49	2.69E-02	Cyclin-dependent kinase 14
CDH17	1.48	1.18E-02	Cadherin 17, LI cadherin (liver-intestine)
FSCN1	1.48	4.28E-02	Fascin actin-bundling protein 1
MGST3	1.48	2.37E-04	Microsomal glutathione S-transferase 3
FREM2	1.48	1.55E-02	FRAS1 related extracellular matrix protein 2
CYP39A1	1.48	1.12E-04	Cytochrome P450 family 39 subfamily A member 1
NRROS	1.48	2.12E-02	Negative regulator of reactive oxygen species
KIF9	1.48	4.94E-02	Kinesin family member 9
ARRDC3	1.47	1.92E-03	Arrestin domain containing 3
CRMP1	1.47	2.00E-03	Collapsin response mediator protein 1
YF5	1.47	2.94E-03	MHC class I antigen YF5
GBE1	1.47	4.99E-04	Glucan (1,4-alpha-), branching enzyme 1
DOCK11	1.47	1.21E-02	Dedicator of cytokinesis 11
COL4A4	1.47	1.38E-02	Collagen, type IV, alpha 4
RUFY2	1.47	1.46E-02	RUN and FYVE domain containing 2
METTL24	1.47	3.84E-03	Methyltransferase like 24
C11ORF34	1.46	1.98E-02	Chromosome 24 open reading frame, human C11orf34
PMS1	1.46	1.41E-02	PMS1 homolog 1, mismatch repair system component
C14ORF1	1.46	6.80E-04	Chromosome 5 open reading frame, human C14orf1
ASB10	1.45	4.77E-03	Ankyrin repeat and SOCS box containing 10
NADK2	1.45	7.12E-04	NAD kinase 2, mitochondrial
CD244	1.45	1.01E-02	CD244 molecule, natural killer cell receptor 2B4
TSC22D1	1.45	4.07E-04	TSC22 domain family, member 1
TRAPPC10	1.44	4.95E-03	Trafficking protein particle complex 10
MEIKIN	1.43	3.24E-02	Meiotic kinetochore factor
MRVLDC3	1.43	3.25E-03	MARVEL domain-containing protein 3
IGFBP4	1.43	2.72E-04	Insulin like growth factor binding protein 4
AVPR1B	1.43	6.54E-03	Arginine vasopressin receptor 1B
LYPD6	1.43	2.99E-02	LY6/PLAUR domain containing 6
NTN3	1.43	4.00E-03	Netrin 1
L3MBTL4	1.42	1.76E-03	L(3)mbt-like 4 (Drosophila)
SERPINA4	1.42	1.57E-04	Serpin peptidase inhibitor, clade A (alpha-1 antiproteinase, antitrypsin), m

CLASP1	1.42	4.85E-02	Cytoplasmic linker associated protein 1
BRS3	1.42	1.53E-03	Bombesin-like receptor 3
SPATA20	1.42	1.20E-02	Spermatogenesis associated 20
C1QA	1.41	2.16E-04	Complement component 1, q subcomponent, A chain
TMSB15B	1.41	2.43E-04	Thymosin beta 15B
CACFD1	1.41	6.50E-04	Calcium channel flower domain containing 1
MPZL2	1.41	1.24E-03	Myelin protein zero like 2
PDE1A	1.40	1.96E-02	Phosphodiesterase 1A, calmodulin-dependent
SERPINA5	1.39	2.41E-03	Serpin peptidase inhibitor, clade A (alpha-1 antiproteinase, antitrypsin), m
TNFAIP2	1.39	5.41E-04	TNF alpha induced protein 2
IL15	1.39	5.08E-04	Interleukin 15
DGKZ	1.39	2.80E-04	Diacylglycerol kinase, zeta
PLEKHM3	1.39	4.95E-02	Pleckstrin homology domain containing, family M, member 3
GNB3	1.39	7.39E-03	Guanine nucleotide binding protein (G protein), beta polypeptide 3
RALGPS1	1.39	3.43E-04	Ral GEF with PH domain and SH3 binding motif 1
LITAF	1.39	1.43E-02	Lipopolysaccharide induced TNF factor
BASP1	1.39	2.81E-04	Brain abundant membrane attached signal protein 1
CYTH4	1.39	3.47E-03	Cytohesin 4
ZCRB1	1.39	1.81E-04	Zinc finger CCHC-type and RNA binding motif containing 1
CYP2J2L6	1.38	4.40E-02	Cytochrome P450 2J2-like 6
CSPG5	1.38	3.53E-02	Chondroitin sulfate proteoglycan 5 (neuroglycan C)
ACSF2	1.38	1.96E-04	Acyl-CoA synthetase family member 2
NEDD4	1.38	4.36E-04	Neural precursor cell expressed, developmentally down-regulated 4, E3 ub
PGP	1.38	1.82E-04	Phosphoglycolate phosphatase
HABP2	1.38	1.41E-03	Hyaluronan binding protein 2
MYO1F	1.38	7.24E-04	Myosin IF
SLC31A1	1.38	3.62E-04	Solute carrier family 31 (copper transporter), member 1
PPP1R17	1.37	4.83E-04	Protein phosphatase 1 regulatory subunit 17
BACH1	1.37	2.13E-04	BTB domain and CNC homolog 1
RAC2	1.37	7.89E-04	Ras-related C3 botulinum toxin substrate 2 (rho family, small GTP bindin
TMCC2	1.37	5.92E-04	Transmembrane and coiled-coil domain family 2
ACADSB	1.37	7.07E-04	Acyl-CoA dehydrogenase, short/branched chain
SIDT1	1.37	1.73E-02	SID1 transmembrane family, member 1
HES4	1.37	1.97E-03	Hairy and enhancer of split 4 (Drosophila)
SPI1	1.37	2.60E-04	Spi-1 proto-oncogene
CLEC3B	1.36	2.92E-04	C-type lectin domain family 3 member B
MARVELD3	1.36	1.82E-02	MARVEL domain containing 3
CNNM4	1.36	4.84E-02	Cyclin M4
SCUBE2	1.36	1.07E-03	Signal peptide, CUB domain, EGF-like 2
SNAP91	1.36	3.69E-03	Synaptosomal-associated protein, 91kDa
RBPJ	1.36	3.49E-03	Recombination signal binding protein for immunoglobulin kappa J region
TMA7	1.36	2.95E-02	Translation machinery associated 7 homolog (S. Cerevisiae)
FLT1	1.36	2.19E-02	Fms-related tyrosine kinase 1
TADA2A	1.35	2.38E-03	Transcriptional adaptor 2A
ALDH1A1	1.35	3.36E-03	Aldehyde dehydrogenase 1 family, member A1
INADL	1.35	4.90E-03	InaD-like (Drosophila)
ITPR3	1.35	9.82E-04	Inositol 1,4,5-trisphosphate receptor, type 3
PKHD1	1.35	9.22E-03	Polycystic kidney and hepatic disease 1 (autosomal recessive)
CYP7A1	1.35	1.17E-02	Cytochrome P450 family 7 subfamily A member 1
PRKCE	1.34	5.21E-03	Protein kinase C, epsilon
CD28	1.34	2.56E-04	CD28 molecule
CYP2B7P1	1.34	3.77E-03	Cytochrome P450, family 2, subfamily B, polypeptide 7 pseudogene 1
RAB36	1.34	3.64E-03	RAB36, member RAS oncogene family
BST1	1.33	1.55E-03	Bone marrow stromal cell antigen 1
SMPDL3A	1.33	1.81E-03	Sphingomyelin phosphodiesterase acid like 3A
GTPBP10	1.33	4.23E-02	GTP-binding protein 10 (putative)

LYRM2	1.33	3.93E-02	LYR motif containing 2
RPS27L	1.33	8.23E-04	Ribosomal protein S27 like
GGA3	1.33	1.32E-02	Golgi-associated, gamma adaptin ear containing, ARF binding protein 3
CASKIN2	1.33	5.73E-04	CASK interacting protein 2
HPS5	1.32	1.36E-03	Hermansky-Pudlak syndrome 5
SLC39A5	1.32	1.24E-02	Solute carrier family 39 (zinc transporter), member 5
PDHA1	1.32	1.68E-02	Pyruvate dehydrogenase (lipoamide) alpha 1
TNFRSF18	1.32	1.55E-02	Tumor necrosis factor receptor superfamily, member 18
FBXO25	1.31	5.69E-04	F-box protein 25
RNF212B	1.31	2.88E-02	Ring finger protein 212B
VGLL2	1.31	4.62E-02	Vestigial like family member 2
ARFGEF3	1.31	4.04E-02	ARFGEF family member 3
CD247	1.30	6.43E-04	CD247 molecule
CYTIP	1.30	4.47E-02	Cytohesin 1 interacting protein
UBXN7	1.30	2.98E-02	UBX domain protein 7
GUCD1	1.30	3.00E-04	Guanylyl cyclase domain containing 1
PIK3CD	1.30	1.21E-03	Phosphatidylinositol-4,5-bisphosphate 3-kinase, catalytic subunit delta
PARD6B	1.30	2.05E-03	Par-6 family cell polarity regulator beta
TIAM2	1.30	5.81E-03	T-cell lymphoma invasion and metastasis 2
ARFIP2	1.30	3.78E-03	ADP-ribosylation factor interacting protein 2
DGKK	1.29	4.17E-02	Diacylglycerol kinase, kappa
GCNT1	1.29	1.41E-02	Glucosaminyl (N-acetyl) transferase 1, core 2
C1H7ORF60	1.29	3.46E-03	Chromosome 1 open reading frame, human C7orf60
FGD3	1.29	4.19E-03	FYVE, RhoGEF and PH domain containing 3
C1QC	1.29	3.51E-04	Complement component 1, q subcomponent, C chain
SUCO	1.29	4.12E-03	SUN domain containing ossification factor
SLCO4A1	1.29	4.49E-04	Solute carrier organic anion transporter family member 4A1
ASMTL	1.29	5.15E-04	Acetylserotonin O-methyltransferase-like
PTPN7	1.28	4.34E-02	Protein tyrosine phosphatase, non-receptor type 7
EWSR1	1.28	1.81E-03	EWS RNA binding protein 1
PTPN5	1.28	3.33E-02	Protein tyrosine phosphatase, non-receptor type 5 (striatum-enriched)
CTSS	1.28	2.94E-04	Cathepsin S
LRRC32	1.28	2.36E-02	Leucine rich repeat containing 32
MAP3K9	1.28	3.16E-02	Mitogen-activated protein kinase kinase kinase 9
CA12	1.27	4.86E-02	Carbonic anhydrase XII
PPL	1.27	3.23E-02	Periplakin
DOCK7	1.27	2.33E-02	Dedicator of cytokinesis 7
PDS5A	1.27	4.27E-03	PDS5, regulator of cohesion maintenance, homolog A (S. Cerevisiae)
TOB2	1.27	8.09E-04	Transducer of ERBB2, 2
MICALL2	1.27	7.06E-03	MICAL like 2
ARL4C	1.27	3.12E-02	ADP-ribosylation factor-like 4C
EREG	1.27	3.90E-03	Epiregulin
DCDC2	1.26	1.78E-03	Doublecortin domain containing 2
PRSS55	1.26	3.53E-03	Protease, serine, 55
ITPRIP	1.26	7.70E-03	Inositol 1,4,5-trisphosphate receptor interacting protein
RHBDF1	1.26	2.26E-02	Rhomboid 5 homolog 1 (Drosophila)
C2orf88	1.26	3.86E-02	Chromosome 2 open reading frame 88
AMPD1	1.26	2.24E-02	Adenosine monophosphate deaminase 1
TMEM238	1.26	7.08E-04	Transmembrane protein 238
ADCY10	1.26	2.60E-02	Adenylate cyclase 10 (soluble)
SESN3	1.26	9.13E-03	Sestrin 3
STX11	1.26	2.86E-04	Syntaxin 11
C20H20ORF11	1.26	1.17E-02	Chromosome 20 open reading frame, human C20orf112
GPR183	1.25	9.52E-03	G protein-coupled receptor 183
LDB3	1.25	1.63E-02	LIM domain binding 3
GADD45B	1.25	2.42E-03	Growth arrest and DNA-damage-inducible, beta

C3orf33	1.25	2.31E-02	Chromosome 3 open reading frame 33
BRWD1	1.25	1.01E-02	Bromodomain and WD repeat domain containing 1
PLEKHD1	1.24	4.61E-02	Pleckstrin homology and coiled-coil domain containing D1
KRT19	1.24	2.10E-03	Keratin 19
BTK	1.24	4.95E-04	Bruton agammaglobulinemia tyrosine kinase
TIMP4	1.24	1.62E-02	TIMP metallopeptidase inhibitor 4
TRAF5	1.24	1.96E-02	TNF receptor associated factor 5
GMIP	1.24	1.15E-02	GEM interacting protein
TSPAN5	1.23	1.06E-02	Tetraspanin 5
PMVK	1.23	6.19E-03	Phosphomevalonate kinase
ADAP1L	1.23	3.03E-02	Arf-GAP with dual PH domains 1-like
BNC2	1.23	4.08E-02	Basonuclin 2
ANKS3	1.23	8.91E-03	Ankyrin repeat and sterile alpha motif domain containing 3
HBAA	1.23	2.86E-04	Hemoglobin, alpha 1
ICOSLG	1.23	5.41E-03	Inducible T-cell co-stimulator ligand
ARSJ	1.23	2.88E-02	Arylsulfatase family, member J
GCLC	1.22	2.91E-04	Glutamate-cysteine ligase catalytic subunit
HPX	1.22	4.87E-04	Hemopexin
LCP2	1.22	1.59E-03	Lymphocyte cytosolic protein 2 (SH2 domain containing leukocyte protei
MAOA	1.22	2.01E-03	Monoamine oxidase A
ZBTB20	1.22	7.86E-04	Zinc finger and BTB domain containing 20
CZH5ORF63	1.22	2.86E-02	Chromosome Z open reading frame, human C5orf63
PARK2	1.22	2.40E-03	Parkin RBR E3 ubiquitin protein ligase
TM4SF4	1.21	4.41E-04	Transmembrane 4 L six family member 4
KCNK5	1.21	1.36E-02	Potassium two pore domain channel subfamily K member 5
LONRF1	1.21	1.43E-03	LON peptidase N-terminal domain and ring finger 1
RNF146	1.21	9.27E-03	Ring finger protein 146
ATG2B	1.21	5.81E-04	Autophagy related 2B
SOBP	1.21	5.11E-03	Sine oculis binding protein homolog
NAPEPLD	1.21	3.98E-04	N-acyl phosphatidylethanolamine phospholipase D
FSTL4	1.21	1.33E-02	Follistatin-like 4
DCBLD1	1.21	5.18E-04	Discoidin, CUB and LCCL domain containing 1
FES	1.20	2.66E-02	Feline sarcoma oncogene
GNG10	1.20	6.99E-04	Guanine nucleotide binding protein (G protein), gamma 10
GPR34	1.20	1.63E-03	G protein-coupled receptor 34
TNFSF15	1.20	2.89E-03	Tumor necrosis factor superfamily member 15
AFF4	1.20	4.66E-02	AF4/FMR2 family, member 4
CCDC89	1.20	1.49E-02	Coiled-coil domain containing 89
GTF2H1	1.20	4.67E-02	General transcription factor IIH subunit 1
PECAM1	1.20	6.64E-04	Platelet/endothelial cell adhesion molecule 1
PPP1R3B	1.20	2.86E-03	Protein phosphatase 1 regulatory subunit 3B
SAMSN1	1.20	1.41E-03	SAM domain, SH3 domain and nuclear localization signals 1
TPBG	1.19	4.95E-02	Trophoblast glycoprotein
SEC31B	1.19	1.26E-03	SEC31 homolog B (S. Cerevisiae)
TMEM52B	1.19	1.08E-02	Transmembrane protein 52B
ZDHHC21	1.19	5.92E-03	Zinc finger DHHC-type containing 21
LIPA	1.19	4.27E-03	Lipase A, lysosomal acid, cholesterol esterase (Wolman disease)
RASL10B	1.19	3.99E-02	RAS-like, family 10, member B
LIMK1	1.19	3.26E-03	LIM domain kinase 1
COCH	1.18	3.71E-03	Cochlin
MKRN3	1.18	8.72E-03	Makorin ring finger protein 3
SLC5A11	1.18	7.69E-03	Solute carrier family 5 (sodium/inositol cotransporter), member 11
SLC2A3	1.18	9.52E-04	Solute carrier family 2 (facilitated glucose transporter), member 3
FYCO1	1.18	2.95E-02	FYVE and coiled-coil domain containing 1
HIST1H2B5L	1.18	2.57E-02	Histone cluster 1, H2B-V-like (similar to human histone cluster 1, class H
C1orf167	1.18	4.95E-02	Chromosome 1 open reading frame 167

ZC3HAV1	1.18	7.89E-04	Zinc finger CCCH-type, antiviral 1
KDM4A	1.18	1.54E-03	Lysine (K)-specific demethylase 4A
SPINK4	1.17	3.10E-02	Serine peptidase inhibitor, Kazal type 4
ZNFX1	1.17	2.19E-03	Zinc finger, NFX1-type containing 1
C2CD5	1.17	3.45E-02	C2 calcium-dependent domain containing 5
ZMYM2	1.17	2.53E-03	Zinc finger, MYM-type 2
LRP4	1.17	1.26E-03	Low density lipoprotein receptor-related protein 4
CREM	1.17	2.52E-02	CAMP responsive element modulator
LIF	1.17	6.35E-03	Leukemia inhibitory factor
ARHGEF16	1.17	3.45E-02	Rho guanine nucleotide exchange factor 16
CSF1R	1.17	4.03E-04	Colony stimulating factor 1 receptor
TRPM8	1.17	1.88E-03	Transient receptor potential cation channel, subfamily M, member 8
LDLRAD3	1.17	1.25E-02	Low density lipoprotein receptor class A domain containing 3
RIDA	1.16	3.09E-03	Reactive intermediate imine deaminase A homolog
C20H20ORF85	1.16	2.31E-02	Chromosome 20 open reading frame, human C20orf85
TMEM161B	1.16	1.49E-03	Transmembrane protein 161B
OPRK1	1.16	2.48E-02	Opioid receptor, kappa 1
CMYA5	1.16	3.45E-02	Cardiomyopathy associated 5
CFC1B	1.16	8.01E-03	Cripto, FRL-1, cryptic family 1B
MFSD2A	1.16	2.61E-03	Major facilitator superfamily domain containing 2A
EPB42	1.16	1.58E-03	Erythrocyte membrane protein band 4.2
M6PR	1.16	1.95E-02	Mannose-6-phosphate receptor, cation dependent
MARK1	1.16	1.41E-02	MAP/microtubule affinity-regulating kinase 1
SEMA5A	1.15	1.58E-02	Sema domain, seven thrombospondin repeats (type 1 and type 1-like), tran
DAPP1	1.15	3.04E-02	Dual adaptor of phosphotyrosine and 3-phosphoinositides
PEX1	1.15	3.81E-03	Peroxisomal biogenesis factor 1
HTR1D	1.15	4.70E-03	5-hydroxytryptamine (serotonin) receptor 1D, G protein-coupled
GRIP1	1.15	2.86E-02	Glutamate receptor interacting protein 1
TRAF3IP3	1.15	2.81E-03	TRAF3 interacting protein 3
LAPTM4B	1.15	3.56E-03	Lysosomal protein transmembrane 4 beta
AGRN	1.15	1.82E-03	Agrin
COMTD1	1.15	1.74E-02	Catechol-O-methyltransferase domain containing 1
CCL1	1.15	1.38E-02	Chemokine (C-C motif) ligand 1
SPEN	1.15	8.44E-03	Spen family transcriptional repressor
SARDH	1.15	1.24E-02	Sarcosine dehydrogenase
PPARG	1.14	2.47E-03	Peroxisome proliferator-activated receptor gamma
PIK3R1	1.14	6.30E-03	Phosphoinositide-3-kinase, regulatory subunit 1 (alpha)
TNFRSF21	1.14	6.29E-04	Tumor necrosis factor receptor superfamily member 21
BRINP1	1.14	3.36E-03	Bone morphogenetic protein/retinoic acid inducible neural-specific 1
LVRN	1.14	1.82E-02	Laeverin
INF2	1.14	2.22E-03	Inverted formin, FH2 and WH2 domain containing
CMTM4	1.14	9.50E-03	CKLF like MARVEL transmembrane domain containing 4
TLR2A	1.13	2.71E-03	Toll-like receptor 2 family member A
MARCO	1.13	4.66E-04	Macrophage receptor with collagenous structure
NCALD	1.13	4.60E-02	Neurocalcin delta
PROK2	1.13	3.74E-02	Prokineticin 2
SORL1	1.13	2.66E-03	Sortilin-related receptor, L(DLR class) A repeats containing
PARP12	1.13	1.07E-03	Poly (ADP-ribose) polymerase family, member 12
ZNF609	1.13	8.03E-03	Zinc finger protein 609
PIGC	1.13	7.78E-03	Phosphatidylinositol glycan anchor biosynthesis class C
GRP	1.13	1.82E-03	Gastrin releasing peptide
UNC45A	1.12	2.69E-03	Unc-45 homolog A (C. Elegans)
C10orf128	1.12	1.03E-02	Chromosome 10 open reading frame 128
MYLK	1.12	1.12E-02	Myosin light chain kinase
ATMIN	1.12	3.63E-02	ATM interactor
THAP7	1.12	3.10E-03	THAP domain containing 7

PARP14	1.12	6.07E-04	Poly (ADP-ribose) polymerase family, member 14
PSD	1.12	3.42E-02	Pleckstrin and Sec7 domain containing
APBB1IP	1.12	1.39E-03	Amyloid beta (A4) precursor protein-binding, family B, member 1 interac
ALOX5	1.12	1.90E-02	Arachidonate 5-lipoxygenase
SALL1	1.12	1.89E-02	Sal-like 1 (Drosophila)
EPSTI1	1.12	4.77E-02	Epithelial stromal interaction 1 (breast)
RAB43	1.12	1.33E-03	RAB43, member RAS oncogene family
IRF4	1.12	1.47E-02	Interferon regulatory factor 4
RXFP3	1.12	2.52E-02	Relaxin/insulin-like family peptide receptor 3
VNN1	1.11	5.49E-03	Vanin 1
CEP104	1.11	3.15E-02	Centrosomal protein 104kDa
DENND6B	1.11	2.98E-02	DENN/MADD domain containing 6B
SLC51A	1.11	1.64E-03	Solute carrier family 51, alpha subunit
TRAFD1	1.11	5.76E-03	TRAF-type zinc finger domain containing 1
KLHL25	1.11	1.74E-03	Kelch-like family member 25
INO80	1.11	4.05E-02	INO80 complex subunit
SCCPDH	1.11	5.31E-04	Saccharopine dehydrogenase (putative)
NME4	1.11	2.88E-02	NME/NM23 nucleoside diphosphate kinase 4
TNFRSF11A	1.10	2.71E-03	Tumor necrosis factor receptor superfamily member 11a
KBTBD12	1.10	1.52E-02	Kelch repeat and BTB domain containing 12
ITPK1	1.10	4.61E-03	Inositol-tetrakisphosphate 1-kinase
SFSWAP	1.10	9.10E-03	Splicing factor, suppressor of white-apricot homolog
ATP6V0C	1.10	4.09E-03	ATPase, H+ transporting, lysosomal 16kDa, V0 subunit c
B4GALT6	1.10	2.80E-03	UDP-Gal:betaGlcNAc beta 1,4- galactosyltransferase, polypeptide 6
MYL3	1.10	2.18E-03	Myosin, light chain 3, alkali; ventricular, skeletal, slow
RASSF2	1.10	1.83E-03	Ras association (RalGDS/AF-6) domain family member 2
ABL2	1.10	1.11E-03	C-abl oncogene 2, non-receptor tyrosine kinase
CRYM	1.10	7.08E-03	Crystallin mu
FAS	1.10	9.97E-03	Fas cell surface death receptor
HDAC9	1.10	2.71E-02	Histone deacetylase 9
TIMP2	1.10	4.51E-03	TIMP metallopeptidase inhibitor 2
CEP85	1.09	3.22E-02	Centrosomal protein 85kDa
NDST4	1.09	3.08E-03	N-deacetylase/N-sulfotransferase (heparan glucosaminyl) 4
NR3C2	1.09	2.72E-02	Nuclear receptor subfamily 3 group C member 2
NPY1R	1.09	2.14E-02	Neuropeptide Y receptor Y1
ZNF318	1.09	2.25E-02	Zinc finger protein 318
TBC1D2B	1.09	3.32E-03	TBC1 domain family member 2B
ZNF608	1.09	7.30E-04	Zinc finger protein 608
HBG1	1.09	1.79E-02	Hemoglobin, gamma A
LRRC8D	1.09	4.61E-03	Leucine rich repeat containing 8 family, member D
PARP4	1.09	6.80E-04	Poly (ADP-ribose) polymerase family, member 4
NAV2	1.09	8.06E-03	Neuron navigator 2
ESR2	1.09	5.03E-03	Estrogen receptor 2 (ER beta)
CRY2	1.09	7.29E-04	Cryptochrome circadian clock 2
SETBP1	1.09	2.12E-02	SET binding protein 1
C2H8ORF84	1.08	1.45E-02	Chromosome 2 open reading frame, human C8orf84
MVK	1.08	1.98E-03	Mevalonate kinase
GALR1L	1.08	8.73E-03	Galanin receptor type 1-like
KLF10	1.08	1.18E-03	Kruppel-like factor 10
AP3M2	1.08	9.71E-03	Adaptor-related protein complex 3, mu 2 subunit
GSTZ1	1.08	1.78E-03	Glutathione S-transferase zeta 1
NGF	1.08	1.89E-02	Nerve growth factor
HERC2	1.08	4.36E-02	HECT and RLD domain containing E3 ubiquitin protein ligase 2
TRAPPC9	1.08	2.45E-02	Trafficking protein particle complex 9
TMEM63A	1.08	1.35E-02	Transmembrane protein 63A
RAP1GAP	1.08	6.66E-04	RAP1 GTPase activating protein

ADAM23	1.07	3.04E-03	ADAM metallopeptidase domain 23
FAM46A	1.07	6.74E-03	Family with sequence similarity 46, member A
WIPI1	1.07	4.36E-03	WD repeat domain, phosphoinositide interacting 1
SCIN	1.07	2.11E-02	Scinderin
ANG	1.07	8.17E-03	Angiogenin, ribonuclease, RNase A family, 5
CAPSL	1.07	2.28E-03	Calcyphosine-like
SLC45A1	1.07	5.66E-03	Solute carrier family 45, member 1
MFSD13A	1.07	4.45E-03	Major facilitator superfamily domain containing 13A
DOCK10	1.07	1.14E-02	Dedicator of cytokinesis 10
CYB5R4	1.07	1.36E-02	Cytochrome b5 reductase 4
TLR3	1.07	4.99E-03	Toll-like receptor 3
LSP1	1.06	7.49E-04	Lymphocyte-specific protein 1
PQLC1	1.06	2.14E-03	PQ loop repeat containing 1
VPRBP	1.06	6.02E-03	Vpr (HIV-1) binding protein
MLL3	1.06	3.46E-02	Myeloid/lymphoid or mixed-lineage leukemia 3
ERRFI1	1.06	2.28E-03	ERBB receptor feedback inhibitor 1
SLC16A12	1.06	9.10E-04	Solute carrier family 16 member 12
XPO4	1.06	1.08E-02	Exportin 4
PINK1	1.05	3.55E-03	PTEN induced putative kinase 1
TBR1	1.05	1.46E-02	T-box, brain, 1
LHX8	1.05	3.01E-02	LIM homeobox 8
GLCCI1	1.05	6.53E-03	Glucocorticoid induced transcript 1
CARD10	1.05	1.67E-03	Caspase recruitment domain family, member 10
IL13	1.05	8.99E-03	Interleukin 13
TP53INP1	1.04	1.94E-03	Tumor protein p53 inducible nuclear protein 1
RTN4	1.04	3.54E-03	Reticulon 4
AOAH	1.04	2.70E-03	Acyloxyacyl hydrolase
ANKS6	1.04	6.57E-03	Ankyrin repeat and sterile alpha motif domain containing 6
CECR5L	1.04	1.17E-03	Cat eye syndrome chromosome region, candidate 5-like
ADPRH	1.04	2.69E-02	ADP-ribosylarginine hydrolase
ORMDL1	1.04	1.12E-03	ORM1-like 1 (S. Cerevisiae)
SLC4A1	1.04	6.39E-03	Solute carrier family 4, anion exchanger, member 1
SKIL	1.04	1.46E-02	SKI-like proto-oncogene
WDR41	1.04	1.28E-02	WD repeat domain 41
FYB	1.03	1.18E-03	FYN binding protein
SPINK7	1.03	1.28E-03	Serine peptidase inhibitor, Kazal type 7 (putative)
TCF7L2	1.03	3.38E-03	Transcription factor 7-like 2 (T-cell specific, HMG-box)
KCTD21	1.03	1.53E-02	Potassium channel tetramerization domain containing 21
PIK3R5	1.03	9.62E-03	Phosphoinositide-3-kinase, regulatory subunit 5
MAP2K5	1.03	3.13E-02	Mitogen-activated protein kinase kinase 5
LNX2	1.03	2.49E-02	Ligand of numb-protein X 2
WWC2	1.03	8.11E-03	WW and C2 domain containing 2
ATP2A3	1.03	2.41E-02	ATPase, Ca++ transporting, ubiquitous
WNT3A	1.03	4.71E-02	Wingless-type MMTV integration site family, member 3A
ZAK	1.03	1.06E-02	Sterile alpha motif and leucine zipper containing kinase AZK
IFT57	1.03	7.40E-03	Intraflagellar transport 57
INHBB	1.03	2.05E-02	Inhibin, beta B
COLGALT2	1.03	1.98E-02	Collagen beta(1-O)galactosyltransferase 2
F2RL1	1.03	9.91E-03	F2R like trypsin receptor 1
ENOX1	1.03	3.87E-03	Ecto-NOX disulfide-thiol exchanger 1
OVCH2	1.02	9.34E-03	Ovochymase 2
SORBS2	1.02	1.61E-03	Sorbin and SH3 domain containing 2
GRAMD1B	1.02	5.66E-03	GRAM domain containing 1B
SAMD10	1.02	3.40E-02	Sterile alpha motif domain containing 10
MCU	1.02	4.70E-02	Mitochondrial calcium uniporter
CPEB4	1.02	3.63E-03	Cytoplasmic polyadenylation element binding protein 4

TLCD1	1.02	4.71E-02	TLC domain containing 1
PARP9	1.02	2.69E-03	Poly(ADP-ribose) polymerase family member 9
TMEM181	1.01	2.91E-03	Transmembrane protein 181
SPOPL	1.01	9.69E-03	Speckle-type POZ protein-like
SLC7A14	1.01	4.69E-03	Solute carrier family 7, member 14
LAPTM5	1.01	1.15E-03	Lysosomal protein transmembrane 5
ASPM	1.01	3.73E-02	Abnormal spindle microtubule assembly
AvBD10	1.01	4.16E-03	Avian beta-defensin 10
FMR1	1.01	1.23E-02	Fragile X mental retardation 1
STARD3NL	1.01	3.78E-03	STARD3 N-terminal like
GCHFR	1.01	3.91E-03	GTP cyclohydrolase I feedback regulator
SREK1	1.01	1.60E-03	Splicing regulatory glutamic acid/lysine-rich protein 1
PAX2	1.01	1.00E-02	Paired box 2
KCNMB1	1.01	2.20E-02	Potassium calcium-activated channel subfamily M regulatory beta subunit
STEAP4	1.01	1.30E-02	STEAP4 metalloreductase
MEGF9	1.01	2.88E-03	Multiple EGF-like-domains 9
PDCD1LG2	1.00	4.58E-02	Programmed cell death 1 ligand 2
SPP1	1.00	2.99E-02	Secreted phosphoprotein 1
STAM	1.00	3.91E-02	Signal transducing adaptor molecule
COL9A3	1.00	3.11E-02	Collagen, type IX, alpha 3
MTTP	1.00	4.25E-03	Microsomal triglyceride transfer protein
TSPAN8	1.00	5.32E-03	Tetraspanin 8
SPON2	1.00	8.27E-03	Spondin 2, extracellular matrix protein
GPR114	1.00	5.55E-03	G protein-coupled receptor 114
KIAA0895L	1.00	2.44E-02	KIAA0895-like
SLC37A4	-1.00	4.70E-02	Solute carrier family 37 member 4
GSG2	-1.00	1.72E-03	Germ cell associated 2 (haspin)
SLC7A3	-1.00	3.21E-02	Solute carrier family 7 (cationic amino acid transporter, y+ system), memb
COMMD3	-1.00	4.34E-03	COMM domain containing 3
PIGU	-1.00	1.18E-02	Phosphatidylinositol glycan anchor biosynthesis class U
TSPAN13	-1.00	4.44E-03	Tetraspanin 13
SYNCRIP	-1.00	2.06E-02	Synaptotagmin binding, cytoplasmic RNA interacting protein
DELE	-1.01	3.26E-03	Death ligand signal enhancer
PDS5B	-1.01	1.13E-02	PDS5, regulator of cohesion maintenance, homolog B (S. Cerevisiae)
ADAMTS15	-1.01	2.81E-02	ADAM metallopeptidase with thrombospondin type 1 motif, 15
NDUFA11	-1.01	1.74E-02	NADH:ubiquinone oxidoreductase subunit A11
MCPH1	-1.01	1.16E-02	Microcephalin 1
PIANP	-1.01	1.88E-02	PILR alpha associated neural protein
TIMP3	-1.01	1.20E-03	TIMP metallopeptidase inhibitor 3
NCKAP5	-1.01	4.36E-03	NCK associated protein 5
PTGFRN	-1.01	4.23E-03	Prostaglandin F2 receptor inhibitor
PDCD4	-1.01	7.64E-03	Programmed cell death 4 (neoplastic transformation inhibitor)
FAM195A	-1.01	1.01E-02	Family with sequence similarity 195, member A
CHMP4C	-1.01	1.24E-02	Charged multivesicular body protein 4C
FTSJ3	-1.01	3.78E-03	FtsJ homolog 3 (E. Coli)
LMNB1	-1.01	1.33E-02	Lamin B1
COX18	-1.01	1.36E-03	COX18 cytochrome c oxidase assembly factor
LANCL2	-1.01	2.12E-02	LanC lantibiotic synthetase component C-like 2 (bacterial)
RCN1	-1.01	9.26E-04	Reticulocalbin 1, EF-hand calcium binding domain
PARPBP	-1.02	2.34E-02	PARP1 binding protein
TTC4	-1.02	1.57E-02	Tetratricopeptide repeat domain 4
MIPEP	-1.02	1.58E-03	Mitochondrial intermediate peptidase
ACTL6A	-1.02	2.04E-03	Actin-like 6A
PCNA	-1.02	7.14E-04	Proliferating cell nuclear antigen
VRK1	-1.02	4.52E-03	Vaccinia related kinase 1
SEH1L	-1.02	2.98E-03	SEH1 like nucleoporin

C9H21ORF2	-1.03	2.05E-03	Chromosome 9 open reading frame, human C21orf2
DSEL	-1.03	5.02E-03	Dermatan sulfate epimerase-like
DLGAP5	-1.03	9.42E-03	Discs large homolog associated protein 5
PIGS	-1.03	1.51E-03	Phosphatidylinositol glycan anchor biosynthesis class S
AURKA	-1.03	1.29E-02	Aurora kinase A
EMP1	-1.03	1.95E-02	Epithelial membrane protein 1
FAM162A	-1.03	1.52E-03	Family with sequence similarity 162, member A
CROT	-1.03	1.02E-03	Carnitine O-octanoyltransferase
CPSF4	-1.03	9.88E-04	Cleavage and polyadenylation specific factor 4, 30kDa
HMMR	-1.03	1.44E-02	Hyaluronan-mediated motility receptor (RHAMM)
SMC2	-1.04	4.67E-03	Structural maintenance of chromosomes 2
RFC2	-1.04	6.75E-04	Replication factor C (activator 1) 2, 40kDa
PPP2R3A	-1.04	2.15E-03	Protein phosphatase 2, regulatory subunit B", alpha
MFN1	-1.04	2.05E-03	Mitofusin 1
CUL1	-1.04	2.45E-02	Cullin 1
ABCA8	-1.04	3.64E-03	ATP-binding cassette, sub-family A (ABC1), member 8
WEE1	-1.04	1.18E-02	WEE1 G2 checkpoint kinase
ELL2	-1.04	6.41E-03	Elongation factor, RNA polymerase II, 2
BLM	-1.04	2.18E-02	Bloom syndrome, RecQ helicase-like
PLK1	-1.04	2.03E-03	Polo like kinase 1
EMC2	-1.04	1.18E-03	ER membrane protein complex subunit 2
LEO1	-1.04	1.01E-03	LEO1 homolog, Paf1/RNA polymerase II complex component
OGFOD3	-1.04	1.57E-03	2-oxoglutarate and iron dependent oxygenase domain containing 3
PPIP5K1	-1.05	3.83E-02	Diphosphoinositol pentakisphosphate kinase 1
KIAA1107	-1.05	3.24E-03	KIAA1107
SLC36A4	-1.05	3.23E-02	Solute carrier family 36 (proton/amino acid symporter), member 4
NID1	-1.05	8.69E-04	Nidogen 1
SPSB4	-1.05	7.63E-03	SplA/ryanodine receptor domain and SOCS box containing 4
SGOL1	-1.05	1.98E-02	Shugoshin-like 1 (S. Pombe)
NUP43	-1.05	8.53E-03	Nucleoporin 43kDa
MTFR1	-1.05	1.06E-02	Mitochondrial fission regulator 1
ETV5	-1.05	4.14E-03	Ets variant 5
BEND4	-1.06	2.74E-03	BEN domain containing 4
AK6	-1.06	2.07E-03	Adenylate kinase 6
GCNT7	-1.06	3.38E-03	Glucosaminyl (N-acetyl) transferase family member 7
NOP16	-1.06	8.53E-04	NOP16 nucleolar protein
ESRRG	-1.06	9.81E-03	Estrogen-related receptor gamma
NFYC	-1.06	5.40E-04	Nuclear transcription factor Y subunit gamma
EIF2S2	-1.06	2.79E-03	Eukaryotic translation initiation factor 2 subunit beta
DLG1	-1.06	3.49E-03	Discs, large homolog 1 (Drosophila)
GTPBP4	-1.06	6.44E-04	GTP binding protein 4
TTC27	-1.06	1.36E-03	Tetratricopeptide repeat domain 27
RSL1D1	-1.07	6.39E-04	Ribosomal L1 domain containing 1
DSCC1	-1.07	1.88E-02	DNA replication and sister chromatid cohesion 1
FARSA	-1.07	3.73E-03	Phenylalanyl-tRNA synthetase alpha subunit
LRRK2	-1.07	2.95E-02	Leucine-rich repeat kinase 2
RPL27A	-1.07	4.15E-02	Ribosomal protein L27a
DIMT1	-1.07	1.62E-03	DIM1 dimethyladenosine transferase 1 homolog (S. Cerevisiae)
NUDT8	-1.07	3.17E-03	Nudix hydrolase 8
PHB	-1.07	6.19E-04	Prohibitin
SHF	-1.07	5.57E-03	Src homology 2 domain containing F
FBXL22	-1.07	3.79E-02	F-box and leucine-rich repeat protein 22
WDR77	-1.07	4.62E-03	WD repeat domain 77
SETD9	-1.07	8.89E-04	SET domain containing 9
AOC3	-1.08	4.93E-03	Amine oxidase, copper containing 3
LPHN3	-1.08	4.18E-02	Latrophilin 3

PLS1	-1.08	4.23E-03	Plastin 1
GUSB	-1.08	4.09E-03	Glucuronidase beta
PYCR2	-1.08	2.85E-03	Pyrroline-5-carboxylate reductase family, member 2
METTL15	-1.08	4.62E-03	Methyltransferase like 15
FAHD1	-1.08	7.10E-04	Fumarylacetoacetate hydrolase domain containing 1
EGFR	-1.08	8.77E-03	Epidermal growth factor receptor
FOXI1	-1.08	3.10E-02	Forkhead box I1
RSPO3	-1.08	1.81E-02	R-spondin 3
MECR	-1.08	2.16E-02	Mitochondrial trans-2-enoyl-CoA reductase
RITA1	-1.08	1.32E-02	RBPJ interacting and tubulin associated 1
PDSS1	-1.08	6.88E-04	Prenyl (decaprenyl) diphosphate synthase, subunit 1
SNRPD1	-1.08	1.01E-02	Small nuclear ribonucleoprotein D1 polypeptide
KIF7	-1.08	2.02E-02	Kinesin family member 7
STC2	-1.09	4.68E-02	Stanniocalcin 2
HADHB	-1.09	1.43E-03	Hydroxyacyl-CoA dehydrogenase/3-ketoacyl-CoA thiolase/enoyl-CoA hy
ASCL1	-1.09	5.51E-03	Achaete-scute complex homolog 1 (Drosophila)
CENPC	-1.09	3.32E-02	Centromere protein C
CYP1B1	-1.09	2.12E-03	Cytochrome P450 1B1
DCTD	-1.09	1.33E-03	DCMP deaminase
DRD1	-1.09	1.13E-03	Dopamine receptor D1
WDR36	-1.09	1.32E-03	WD repeat domain 36
FAM124A	-1.10	1.22E-02	Family with sequence similarity 124A
CENPO	-1.10	2.64E-02	Centromere protein O
ARMC9	-1.10	3.13E-03	Armadillo repeat containing 9
LCMT1	-1.10	2.10E-03	Leucine carboxyl methyltransferase 1
BLVRA	-1.10	1.51E-03	Biliverdin reductase A
DLC1	-1.10	1.05E-03	Deleted in liver cancer 1
DAP3	-1.11	6.27E-04	Death associated protein 3
FCGBP	-1.11	1.82E-02	Fc fragment of IgG binding protein
BTF3L4	-1.11	8.40E-04	Basic transcription factor 3-like 4
CYR61	-1.11	9.31E-04	Cysteine rich angiogenic inducer 61
SRM	-1.11	1.11E-03	Spermidine synthase
ALKBH8	-1.11	1.62E-02	AlkB homolog 8, tRNA methyltransferase
ADAM12	-1.11	4.63E-02	ADAM metallopeptidase domain 12
RPA3	-1.11	6.96E-04	Replication protein A3
GMNN	-1.11	5.52E-03	Geminin, DNA replication inhibitor
RFESD	-1.12	1.59E-03	Rieske Fe-S domain containing
SFTPA	-1.12	5.41E-03	Surfactant, pulmonary-associated protein A
CRYBA4	-1.12	6.80E-03	Crystallin, beta A4
CSTF2	-1.12	1.22E-03	Cleavage stimulation factor, 3' pre-RNA, subunit 2, 64kDa
TMEM82	-1.12	1.85E-03	Transmembrane protein 82
VIPR1	-1.12	2.08E-03	Vasoactive intestinal peptide receptor 1
ORC4	-1.12	1.14E-03	Origin recognition complex subunit 4
LGALSL	-1.12	2.27E-03	Lectin, galactoside-binding-like
CDC20	-1.12	2.01E-03	Cell division cycle 20
USP7	-1.13	2.27E-02	Ubiquitin specific peptidase 7 (herpes virus-associated)
BAG3	-1.13	2.34E-02	BCL2 associated athanogene 3
TFB2M	-1.13	5.69E-04	Transcription factor B2, mitochondrial
NOLC1	-1.13	5.74E-04	Nucleolar and coiled-body phosphoprotein 1
PDGFRL	-1.13	8.83E-04	Platelet-derived growth factor receptor-like
TMCO4	-1.13	5.91E-03	Transmembrane and coiled-coil domains 4
MAP1A	-1.13	1.94E-03	Microtubule-associated protein 1A
NEK2	-1.13	2.46E-03	NIMA related kinase 2
LPAR6	-1.13	3.86E-03	Lysophosphatidic acid receptor 6
SLC22A5	-1.13	1.61E-03	Solute carrier family 22 (organic cation transporter), member 5
EPB41L3	-1.13	1.65E-03	Erythrocyte membrane protein band 4.1-like 3

CDCA7L	-1.13	2.71E-03	Cell division cycle associated 7 like
IMPA2	-1.14	1.88E-03	Inositol(myo)-1(or 4)-monophosphatase 2
RTEL1	-1.14	1.38E-02	Regulator of telomere elongation helicase 1
POLE	-1.14	4.40E-02	Polymerase (DNA) epsilon, catalytic subunit
PCK1	-1.14	1.47E-02	Phosphoenolpyruvate carboxykinase 1 (soluble)
SLC35G2	-1.14	1.18E-02	Solute carrier family 35 member G2
TMEM9	-1.14	1.73E-02	Transmembrane protein 9
UHRF1	-1.15	3.56E-04	Ubiquitin-like with PHD and ring finger domains 1
NEFH	-1.15	3.96E-03	Neurofilament, heavy polypeptide
CNTF	-1.15	1.84E-02	Ciliary neurotrophic factor
APOA5	-1.15	3.52E-03	Apolipoprotein A-V
CA9	-1.15	8.38E-04	Carbonic anhydrase IX
AGBL4	-1.15	3.33E-02	ATP/GTP binding protein-like 4
RNF207	-1.15	2.28E-02	Ring finger protein 207
GAD2	-1.15	2.04E-02	Glutamate decarboxylase 2 (pancreatic islets and brain, 65kDa)
MIS12	-1.15	5.71E-03	MIS12 kinetochore complex component
NFKBIA	-1.15	2.60E-03	Nuclear factor of kappa light polypeptide gene enhancer in B-cells inhibit
CIRBP	-1.16	1.92E-03	Cold inducible RNA binding protein
PRELID1	-1.16	3.88E-04	PRELI domain containing 1
TMEM74	-1.16	2.44E-02	Transmembrane protein 74
TERF1	-1.16	3.12E-03	Telomeric repeat binding factor (NIMA-interacting) 1
PCBP3	-1.16	3.08E-02	Poly(rC) binding protein 3
MKNK1	-1.16	3.64E-02	MAP kinase interacting serine/threonine kinase 1
ABCG8	-1.16	3.54E-03	ATP binding cassette subfamily G member 8
LAMB1	-1.16	8.73E-04	Laminin, beta 1
MEIOB	-1.17	4.09E-02	Meiosis specific with OB domains
ABCG5	-1.17	2.63E-03	ATP binding cassette subfamily G member 5
ZGRF1	-1.17	1.12E-03	Zinc finger GRF-type containing 1
NIP7	-1.17	7.40E-04	NIP7, nucleolar pre-rRNA processing protein
RBL1	-1.17	1.72E-03	Retinoblastoma-like 1 (p107)
TYW1	-1.17	2.25E-02	TRNA-yW synthesizing protein 1 homolog (S. Cerevisiae)
AGPAT6	-1.17	3.80E-04	1-acylglycerol-3-phosphate O-acyltransferase 6 (lysophosphatidic acid ac
BPNT1	-1.17	4.78E-04	3'(2'), 5'-bisphosphate nucleotidase 1
MGARP	-1.18	9.93E-04	Mitochondria localized glutamic acid rich protein
TMTC2	-1.18	3.64E-04	Transmembrane and tetratricopeptide repeat containing 2
CRCP	-1.18	1.81E-02	CGRP receptor component
NDC1	-1.18	9.04E-04	NDC1 transmembrane nucleoporin
KIF11	-1.18	4.62E-03	Kinesin family member 11
DUT	-1.18	7.73E-04	Deoxyuridine triphosphatase
SASS6	-1.18	6.23E-04	Spindle assembly 6 homolog (C. Elegans)
CTGF	-1.18	3.89E-04	Connective tissue growth factor
EXO1	-1.18	1.18E-02	Exonuclease 1
RRM2	-1.18	3.71E-03	Ribonucleotide reductase M2
COL4A5	-1.18	3.71E-02	Collagen, type IV, alpha 5
RWDD1	-1.18	3.42E-04	RWD domain containing 1
KLHDC8B	-1.19	2.83E-02	Kelch domain containing 8B
KIAA1524	-1.19	1.76E-03	KIAA1524
CCDC80	-1.19	8.64E-04	Coiled-coil domain containing 80
CRYAB	-1.19	2.26E-02	Crystallin, alpha B
RIC3	-1.19	1.23E-03	RIC3 acetylcholine receptor chaperone
HELQ	-1.19	3.04E-02	Helicase, POLQ-like
CETN1	-1.19	1.74E-03	Centrin, EF-hand protein, 1
POLR3A	-1.19	4.83E-02	Polymerase (RNA) III subunit A
FARSB	-1.19	5.09E-04	Phenylalanyl-tRNA synthetase beta subunit
LGR6	-1.20	4.67E-02	Leucine-rich repeat containing G protein-coupled receptor 6
NOC2L	-1.20	6.70E-04	NOC2 like nucleolar associated transcriptional repressor

NASP	-1.20	4.78E-04	Nuclear autoantigenic sperm protein (histone-binding)
PPIP5K2	-1.20	1.07E-03	Diphosphoinositol pentakisphosphate kinase 2
MYO15B	-1.20	1.44E-02	Myosin XVB pseudogene
TK1	-1.20	1.59E-03	Thymidine kinase 1, soluble
FOLH1	-1.20	8.93E-04	Folate hydrolase (prostate-specific membrane antigen) 1
PELI2	-1.20	2.64E-02	Pellino homolog 2 (Drosophila)
GLIS1	-1.20	2.08E-02	GLIS family zinc finger 1
C11orf24	-1.20	1.17E-02	Chromosome 11 open reading frame 24
KIF18A	-1.21	3.11E-02	Kinesin family member 18A
EME1	-1.21	1.60E-03	Essential meiotic structure-specific endonuclease 1
CUEDC2	-1.21	9.50E-04	CUE domain containing 2
KLF9	-1.21	1.12E-03	Kruppel-like factor 9
TIMM8A	-1.21	6.20E-03	Translocase of inner mitochondrial membrane 8 homolog A (yeast)
CCNB2	-1.21	2.68E-03	Cyclin B2
MT3	-1.21	2.45E-02	Metallothionein 3
ALG14	-1.22	6.00E-04	ALG14, UDP-N-acetylglucosaminyltransferase subunit
RBMS3	-1.22	1.12E-02	RNA binding motif, single stranded interacting protein 3
SRRL	-1.22	3.45E-03	Serine racemase-like
RPH3A	-1.22	4.78E-02	Rabphilin 3A homolog (mouse)
TOP2A	-1.22	1.00E-03	Topoisomerase (DNA) II alpha 170kDa
CENPK	-1.22	1.37E-03	Centromere protein K
TPPP2	-1.23	5.95E-03	Tubulin polymerization-promoting protein family member 2
NOTUM	-1.23	1.14E-02	Notum pectinacetylesterase homolog (Drosophila)
PLEK	-1.23	1.08E-02	Pleckstrin
MPC1	-1.23	7.33E-04	Mitochondrial pyruvate carrier 1
FANCG	-1.23	5.28E-03	Fanconi anemia complementation group G
ARSB	-1.23	1.98E-03	Arylsulfatase B
ATP2B2	-1.23	2.23E-03	ATPase, Ca++ transporting, plasma membrane 2
TXNRD3	-1.23	2.91E-04	Thioredoxin reductase 3
CSRP2BP	-1.24	9.37E-03	CSRP2 binding protein
RPS25	-1.24	2.91E-03	Ribosomal protein S25
SLC1A4	-1.24	3.68E-02	Solute carrier family 1 (glutamate/neutral amino acid transporter), membe
HEG1	-1.24	3.95E-02	Heart development protein with EGF-like domains 1
TICRR	-1.24	5.47E-04	TOPBP1-interacting checkpoint and replication regulator
FEN1	-1.24	8.41E-04	Flap structure-specific endonuclease 1
PFKFB4	-1.24	4.91E-04	6-phosphofructo-2-kinase/fructose-2,6-biphosphatase 4
KIF2C	-1.24	1.95E-03	Kinesin family member 2C
ABCB1LA	-1.24	3.47E-04	ATP-binding cassette, sub-family B (MDR/TAP), member 1-like A
MLH3	-1.25	7.01E-03	MutL homolog 3
EIF5A2	-1.25	3.44E-03	Eukaryotic translation initiation factor 5A2
TOP3A	-1.25	2.38E-02	Topoisomerase (DNA) III alpha
OLA1	-1.25	3.97E-04	Obg-like ATPase 1
DEAF1	-1.25	1.97E-02	DEAF1, transcription factor
SAMHD1	-1.25	3.43E-04	SAM domain and HD domain 1
HYKK	-1.25	1.67E-03	Hydroxylysine kinase
APITD1	-1.25	1.91E-03	Apoptosis-inducing, TAF9-like domain 1
SERBP1	-1.26	6.63E-03	SERPINE1 mRNA binding protein 1
SNRPA1	-1.26	3.93E-03	Small nuclear ribonucleoprotein polypeptide A'
FBLN2	-1.26	1.48E-03	Fibulin 2
ATR	-1.26	5.47E-04	ATR serine/threonine kinase
TCTE1	-1.26	4.42E-02	T-complex-associated-testis-expressed 1
MANSC1	-1.26	7.42E-03	MANSC domain containing 1
RPSA	-1.26	4.71E-03	Ribosomal protein SA
FANCC	-1.27	2.32E-02	Fanconi anemia complementation group C
DPP6	-1.27	4.78E-02	Dipeptidyl-peptidase 6
CA4	-1.27	8.66E-03	Carbonic anhydrase IV

CHCHD3	-1.27	2.26E-04	Coiled-coil-helix-coiled-coil-helix domain containing 3
DDX24	-1.27	2.80E-04	DEAD (Asp-Glu-Ala-Asp) box helicase 24
BRCA2	-1.27	1.08E-03	Breast cancer 2
POMC	-1.28	1.17E-02	Proopiomelanocortin
XPNPEP1	-1.28	3.98E-04	X-prolyl aminopeptidase (aminopeptidase P) 1, soluble
CHTF8	-1.28	1.91E-02	Chromosome transmission fidelity factor 8
TDP1	-1.28	3.86E-04	Tyrosyl-DNA phosphodiesterase 1
CKAP2	-1.28	3.17E-02	Cytoskeleton associated protein 2
TTLL11	-1.29	5.01E-03	Tubulin tyrosine ligase-like family, member 11
HAUS3	-1.29	8.93E-03	HAUS augmin-like complex, subunit 3
PLA2R1	-1.29	3.04E-03	Phospholipase A2 receptor 1, 180kDa
ORC5	-1.29	3.42E-03	Origin recognition complex subunit 5
B4GALNT3	-1.29	1.61E-02	Beta-1,4-N-acetyl-galactosaminyl transferase 3
ATAD3A	-1.29	4.14E-04	ATPase family, AAA domain containing 3A
EPHA5	-1.29	2.64E-02	EPH receptor A5
NUP93	-1.30	7.76E-04	Nucleoporin 93kDa
TBL3	-1.30	9.47E-04	Transducin beta like 3
ASPH	-1.30	2.22E-03	Aspartate beta-hydroxylase
ACBD5	-1.30	2.04E-03	Acyl-CoA binding domain containing 5
CHCHD4	-1.30	4.24E-04	Coiled-coil-helix-coiled-coil-helix domain containing 4
SPAG6	-1.30	2.01E-02	Sperm associated antigen 6
CPED1	-1.30	3.15E-04	Cadherin-like and PC-esterase domain containing 1
NDUFA10	-1.30	1.67E-03	NADH:ubiquinone oxidoreductase subunit A10
PBK	-1.30	3.21E-03	PDZ binding kinase
PPP6R2	-1.31	8.55E-03	Protein phosphatase 6, regulatory subunit 2
MAN1B1	-1.31	2.53E-04	Mannosidase alpha class 1B member 1
C10orf2	-1.32	4.37E-04	Chromosome 10 open reading frame 2
KIF15	-1.33	1.05E-03	Kinesin family member 15
CAMK2A	-1.33	2.32E-02	Calcium/calmodulin-dependent protein kinase II alpha
LIFR	-1.33	5.49E-04	Leukemia inhibitory factor receptor alpha
CCDC13	-1.33	6.78E-04	Coiled-coil domain containing 13
CENPM	-1.33	2.01E-04	Centromere protein M
NEIL3	-1.33	2.29E-03	Nei like DNA glycosylase 3
TMEM130	-1.33	3.22E-03	Transmembrane protein 130
ADRA1D	-1.33	1.52E-02	Adrenoceptor alpha 1D
SLC41A3	-1.33	3.49E-03	Solute carrier family 41 member 3
RFC4	-1.34	2.34E-03	Replication factor C subunit 4
BRIP1	-1.34	7.33E-03	BRCA1 interacting protein C-terminal helicase 1
TGM6	-1.34	3.24E-02	Transglutaminase 6
URAH	-1.34	2.35E-04	5-hydroxyisourate hydrolase
HMX1	-1.34	3.99E-03	H6 family homeobox 1
PTMA	-1.35	1.38E-02	Prothymosin, alpha
C1H2ORF40	-1.35	9.52E-04	Chromosome 1 open reading frame, human C2orf40
RPP38	-1.36	4.77E-03	Ribonuclease P/MRP 38kDa subunit
IL1RL1	-1.36	9.98E-04	Interleukin 1 receptor like 1
ABCB10	-1.36	4.34E-02	ATP binding cassette subfamily B member 10
EIF3A	-1.36	2.59E-02	Eukaryotic translation initiation factor 3 subunit A
ORC1	-1.36	3.24E-02	Origin recognition complex subunit 1
MELTF	-1.37	5.60E-03	Melanotransferrin
CDC7	-1.37	8.88E-04	Cell division cycle 7
C6H10ORF107	-1.37	1.42E-02	Chromosome 6 open reading frame, human C10orf107
LTBP2	-1.37	3.74E-02	Latent transforming growth factor beta binding protein 2
FAM46B	-1.37	5.06E-03	Family with sequence similarity 46, member B
TTRL	-1.38	1.20E-03	Transthyretin-like
DCK	-1.38	3.78E-04	Deoxycytidine kinase
TTK	-1.38	1.41E-03	TTK protein kinase

RRM1	-1.38	3.56E-04	Ribonucleotide reductase catalytic subunit M1
SNAPC4	-1.38	1.71E-02	Small nuclear RNA activating complex, polypeptide 4, 190kDa
CA13	-1.38	2.70E-04	Carbonic anhydrase XIII
TMEM37	-1.39	4.11E-04	Transmembrane protein 37
FABP5	-1.39	5.73E-04	Fatty acid binding protein 5 (psoriasis-associated)
LAMA2	-1.39	7.11E-03	Laminin, alpha 2
SUN2	-1.39	4.58E-02	Sad1 and UNC84 domain containing 2
ESCO2	-1.39	2.12E-04	Establishment of sister chromatid cohesion N-acetyltransferase 2
PTGER4	-1.39	4.53E-03	Prostaglandin E receptor 4
CDCA7	-1.40	2.46E-03	Cell division cycle associated 7
LINC00954	-1.40	4.31E-02	Long intergenic non-protein coding RNA 954
CD101	-1.40	3.48E-02	CD101 molecule
C11orf31	-1.40	8.64E-04	Chromosome 11 open reading frame 31
C8ORF22	-1.40	2.61E-04	Chromosome 2 open reading frame, human C8orf22
WDR3	-1.40	1.09E-03	WD repeat domain 3
RFX5	-1.40	3.32E-02	Regulatory factor X, 5 (influences HLA class II expression)
SERPINF1	-1.41	1.29E-03	Serpin peptidase inhibitor, clade F (alpha-2 antiplasmin, pigment epitheliu
C5H15ORF52	-1.41	1.87E-02	Chromosome 5 open reading frame, human C15orf52
FAM57A	-1.41	3.88E-02	Family with sequence similarity 57, member A
CCNA2	-1.41	3.66E-04	Cyclin A2
PLCXD1	-1.41	1.17E-03	Phosphatidylinositol-specific phospholipase C, X domain containing 1
BTC	-1.41	1.63E-03	Betacellulin
ATAD5	-1.41	1.73E-03	ATPase family, AAA domain containing 5
ZFYVE9	-1.41	3.60E-02	Zinc finger, FYVE domain containing 9
PSMC3IP	-1.41	6.88E-04	PSMC3 interacting protein
UMODL1	-1.41	3.54E-02	Uromodulin-like 1
CRISPLD1	-1.42	8.27E-04	Cysteine-rich secretory protein LCCL domain containing 1
CENPJ	-1.42	1.94E-02	Centromere protein J
FAXC	-1.43	2.44E-03	Failed axon connections homolog
AKR1B10	-1.43	1.82E-03	Aldo-keto reductase family 1, member B10 (aldose reductase)
NUF2	-1.43	1.23E-03	NUF2, NDC80 kinetochore complex component
EFHC2	-1.44	1.26E-03	EF-hand domain (C-terminal) containing 2
KLHL29	-1.44	1.73E-03	Kelch like family member 29
FIGNL1	-1.44	8.37E-04	Fidgetin like 1
SHISA6	-1.44	2.66E-02	Shisa homolog 6 (Xenopus laevis)
CES1	-1.44	1.78E-03	Carboxylesterase 1 (monocyte/macrophage serine esterase 1)
L3HYPDH	-1.44	3.81E-03	Trans-L-3-hydroxyproline dehydratase
IL1R2	-1.44	3.57E-02	Interleukin 1 receptor type 2
UNC93A	-1.45	1.57E-02	Unc-93 homolog A (C. Elegans)
ATP8A2	-1.45	4.06E-02	ATPase, aminophospholipid transporter, class I, type 8A, member 2
GPR88	-1.45	4.52E-02	G protein-coupled receptor 88
RTTN	-1.45	2.69E-03	Rotatin
BORA	-1.45	1.57E-02	Bora, aurora kinase A activator
FAM40B	-1.45	2.12E-02	Family with sequence similarity 40, member B
DPYSL4	-1.45	3.39E-02	Dihydropyrimidinase-like 4
PHF14	-1.45	9.39E-03	PHD finger protein 14
NAT10	-1.45	2.81E-04	N-acetyltransferase 10
FAM149A	-1.46	9.25E-04	Family with sequence similarity 149 member A
SLC39A8	-1.46	1.27E-03	Solute carrier family 39 (zinc transporter), member 8
DNA2	-1.47	8.17E-04	DNA replication helicase/nuclease 2
INS	-1.47	3.69E-02	Insulin
ITM2C	-1.47	1.04E-02	Integral membrane protein 2C
MDK	-1.48	4.44E-04	Midkine (neurite growth-promoting factor 2)
AMY2A	-1.48	2.41E-02	Amylase, alpha 2A; pancreatic
ADCY1	-1.48	2.41E-03	Adenylate cyclase 1 (brain)
MMEL1	-1.49	2.64E-02	Membrane metallo-endopeptidase-like 1

MB21D1	-1.49	8.98E-03	Mab-21 domain containing 1
RAD9B	-1.49	1.31E-02	RAD9 checkpoint clamp component B
RAD52	-1.49	1.60E-03	RAD52 homolog, DNA repair protein
GRIA2	-1.49	1.24E-02	Glutamate receptor, ionotropic, AMPA 2
TFPI	-1.50	2.21E-03	Tissue factor pathway inhibitor (lipoprotein-associated coagulation inhibit
ORC6	-1.50	1.78E-04	Origin recognition complex subunit 6
RGS6	-1.50	2.40E-02	Regulator of G-protein signaling 6
ESPN	-1.50	2.72E-02	Espin
RIF1	-1.50	1.22E-03	Replication timing regulatory factor 1
MRPL50	-1.51	3.61E-02	Mitochondrial ribosomal protein L50
CACNA1S	-1.51	3.40E-02	Calcium channel, voltage-dependent, L type, alpha 1S subunit
E2F8	-1.51	9.49E-03	E2F transcription factor 8
SLBP	-1.51	3.38E-04	Stem-loop binding protein
RRP9	-1.51	6.97E-04	Ribosomal RNA processing 9, small subunit (SSU) processome componer
CBR1	-1.51	6.07E-03	Carbonyl reductase 1
PHYHIPL	-1.51	3.10E-02	Phytanoyl-CoA 2-hydroxylase interacting protein-like
MCM6	-1.51	2.31E-04	Minichromosome maintenance complex component 6
MYCN	-1.51	1.26E-02	V-myc avian myelocytomatosis viral oncogene neuroblastoma derived hor
TENM2	-1.52	1.82E-02	Teneurin transmembrane protein 2
TPM2	-1.52	3.62E-02	Tropomyosin 2 (beta)
PAK6	-1.52	2.94E-03	P21 protein (Cdc42/Rac)-activated kinase 6 [Source:EntrezGene;Acc:569
UHRF1BP1L	-1.52	1.38E-02	UHRF1 binding protein 1-like
UPP1	-1.53	7.09E-04	Uridine phosphorylase 1
RAB20	-1.53	1.26E-04	RAB20, member RAS oncogene family
APCDD1L	-1.53	1.20E-03	Adenomatosis polyposis coli down-regulated 1-like
TCF7	-1.53	2.24E-03	Transcription factor 7 (T-cell specific, HMG-box)
FEZ2	-1.54	1.36E-03	Fasciculation and elongation protein zeta 2
AMN	-1.54	2.57E-04	Amnion associated transmembrane protein
KIAA1161	-1.54	3.74E-04	KIAA1161
POP5	-1.54	3.16E-04	POP5 homolog, ribonuclease P/MRP subunit
RBBP7	-1.55	1.67E-04	Retinoblastoma binding protein 7
DNM1L	-1.55	2.49E-02	Dynamin 1-like
DNMT3B	-1.55	6.10E-04	DNA (cytosine-5-)-methyltransferase 3 beta
TESC	-1.55	1.11E-03	Tescalcin
KCNG3	-1.55	2.04E-02	Potassium voltage-gated channel modifier subfamily G member 3
CDC45	-1.55	3.78E-04	Cell division cycle 45
RAD51	-1.55	6.61E-03	RAD51 recombinase
LRRC14B	-1.56	4.26E-02	Leucine rich repeat containing 14B
NEGR1	-1.56	1.26E-02	Neuronal growth regulator 1
SLC6A6	-1.56	1.10E-02	Solute carrier family 6 (neurotransmitter transporter), member 6
PKP2	-1.56	2.13E-04	Plakophilin 2
NDUFS4	-1.56	8.53E-04	NADH dehydrogenase (ubiquinone) Fe-S protein 4, 18kDa (NADH-coenz
FBN2	-1.56	1.38E-03	Fibrillin 2
ESF1	-1.57	3.19E-04	ESF1 nucleolar pre-rRNA processing protein homolog
CADM3	-1.57	1.06E-02	Cell adhesion molecule 3
HHATL	-1.57	4.22E-03	Hedgehog acyltransferase-like
INIP	-1.57	4.61E-02	INTS3 and NABP interacting protein
TSPAN7	-1.57	1.96E-02	Tetraspanin 7
ACOT12	-1.58	3.46E-03	Acyl-CoA thioesterase 12
CIT	-1.58	3.41E-03	Citron rho-interacting serine/threonine kinase
NAT8L	-1.58	9.61E-03	N-acetyltransferase 8 like
DIO2	-1.58	3.97E-03	Deiodinase, iodothyronine, type II
MCM5	-1.58	3.24E-04	Minichromosome maintenance complex component 5
HHIP	-1.58	6.66E-04	Hedgehog interacting protein
PRSSL1	-1.58	3.38E-02	Protease, serine-like 1
ACCS	-1.58	1.25E-02	1-aminocyclopropane-1-carboxylate synthase homolog

TDRD9	-1.59	4.63E-02	Tudor domain containing 9
TYMS	-1.59	9.37E-05	Thymidylate synthetase
ISM1	-1.59	5.32E-04	Isthmin 1, angiogenesis inhibitor
VWC2	-1.59	1.48E-02	Von Willebrand factor C domain containing 2
PPP1R14D	-1.59	4.80E-03	Protein phosphatase 1, regulatory (inhibitor) subunit 14D
BGLAP	-1.60	3.24E-02	Bone gamma-carboxyglutamate (gla) protein
PHYHD1	-1.60	1.67E-03	Phytanoyl-CoA dioxygenase domain containing 1
NPM3	-1.61	9.81E-05	Nucleophosmin/nucleoplasmin 3
PACRGL	-1.61	1.73E-02	PARK2 co-regulated like
VSNL1	-1.61	2.28E-04	Visinin-like 1
FAP	-1.61	4.94E-02	Fibroblast activation protein, alpha
HELB	-1.61	1.74E-02	Helicase (DNA) B
TSHZ2	-1.61	2.37E-02	Teashirt zinc finger homeobox 2
GSE1	-1.62	1.12E-02	Gse1 coiled-coil protein
TRIP13	-1.62	6.76E-04	Thyroid hormone receptor interactor 13
FAM110B	-1.62	6.49E-03	Family with sequence similarity 110 member B
ITIH6	-1.63	2.83E-04	Inter-alpha-trypsin inhibitor heavy chain family member 6
OXTR	-1.63	3.49E-02	Oxytocin receptor
TUB	-1.63	4.67E-02	Tubby bipartite transcription factor
SURF2	-1.63	4.03E-02	Surfeit 2
PDE3A	-1.63	1.08E-02	Phosphodiesterase 3A, cGMP-inhibited
VWDE	-1.63	2.71E-02	Von Willebrand factor D and EGF domains
HOXC11	-1.64	1.78E-02	Homeobox C11
TIMM17A	-1.64	1.57E-04	Translocase of inner mitochondrial membrane 17 homolog A (yeast)
MCM2	-1.64	2.29E-04	Minichromosome maintenance complex component 2
LPL	-1.65	4.54E-04	Lipoprotein lipase
STXBP5	-1.65	3.04E-02	Syntaxin binding protein 5 (tomosyn)
TUBA3E	-1.65	3.88E-02	Tubulin, alpha 3e
SLC25A32	-1.65	1.04E-04	Solute carrier family 25 member 32
COL14A1	-1.66	1.82E-04	Collagen, type XIV, alpha 1
CHAF1B	-1.66	3.96E-04	Chromatin assembly factor 1 subunit B
SOGA3	-1.66	4.14E-03	SOGA family member 3
TMEM246	-1.66	3.30E-02	Transmembrane protein 246
BRCA1	-1.66	3.41E-03	Breast cancer 1
USH2A	-1.67	1.96E-03	Usher syndrome 2A (autosomal recessive, mild)
THL	-1.67	1.28E-02	Tyrosine hydroxylase-like
SELE	-1.67	4.58E-02	Selectin E (endothelial adhesion molecule 1)
SGK196	-1.67	9.59E-05	Protein kinase-like protein SgK196
FDXR	-1.67	8.33E-04	Ferredoxin reductase
DGUOK	-1.68	1.15E-04	Deoxyguanosine kinase
MASTL	-1.68	3.08E-03	Microtubule associated serine/threonine kinase like
UCHL5	-1.68	3.79E-04	Ubiquitin C-terminal hydrolase L5
ANKRD52	-1.68	1.29E-02	Ankyrin repeat domain 52
IP6K2	-1.68	9.81E-05	Inositol hexakisphosphate kinase 2
LOXL1	-1.68	5.85E-04	Lysyl oxidase-like 1
FAAP24	-1.68	4.30E-04	Fanconi anemia core complex associated protein 24
GINS1	-1.69	6.45E-05	GINS complex subunit 1
HAPLN1	-1.69	3.59E-03	Hyaluronan and proteoglycan link protein 1
SUSD1	-1.69	2.44E-02	Sushi domain containing 1
SLC11A1	-1.69	4.62E-02	Solute carrier family 11 (proton-coupled divalent metal ion transporter), m
FN3K	-1.69	1.66E-04	Fructosamine 3 kinase
TFB1M	-1.69	2.88E-02	Transcription factor B1, mitochondrial
BARD1	-1.70	9.33E-03	BRCA1 associated RING domain 1
RAD54B	-1.71	1.07E-02	RAD54 homolog B (S. Cerevisiae)
LAG3	-1.71	3.42E-02	Lymphocyte activating 3
DTL	-1.71	4.14E-03	Denticleless E3 ubiquitin protein ligase homolog

THBS4	-1.71	4.67E-03	Thrombospondin 4
SLC13A3	-1.72	1.60E-03	Solute carrier family 13 (sodium-dependent dicarboxylate transporter), me
ST8SIA3	-1.72	1.16E-02	ST8 alpha-N-acetyl-neuraminide alpha-2,8-sialyltransferase 3
AIFM2	-1.72	8.26E-04	Apoptosis inducing factor, mitochondria associated 2
CFAP206	-1.72	4.75E-02	Cilia and flagella associated protein 206
TADA1	-1.73	1.36E-03	Transcriptional adaptor 1
ABI2	-1.74	1.53E-02	Abl-interactor 2
OBSL1	-1.74	1.62E-02	Obscurin-like 1
YTHDC1	-1.74	3.26E-02	YTH domain containing 1
MYC	-1.75	1.77E-04	V-myc avian myelocytomatosis viral oncogene homolog
MAST2	-1.76	1.50E-02	Microtubule associated serine/threonine kinase 2
EPB41L1	-1.76	7.90E-03	Erythrocyte membrane protein band 4.1-like 1
IRS4	-1.76	8.57E-04	Insulin receptor substrate 4
MAML1	-1.76	4.45E-02	Mastermind-like 1 (Drosophila)
SLC9A3R2	-1.76	1.49E-02	SLC9A3 regulator 2
CCDC191	-1.77	4.08E-02	Coiled-coil domain containing 191
RGCC	-1.77	7.49E-03	Regulator of cell cycle
EZR	-1.77	8.02E-05	Ezrin
CYP2AC7	-1.78	1.75E-02	Cytochrome P450, family 2, subfamily AC, polypeptide 7
ARNTL2	-1.79	1.85E-03	Aryl hydrocarbon receptor nuclear translocator-like 2
FZD2	-1.79	1.24E-04	Frizzled class receptor 2
CDT1	-1.79	2.29E-04	Chromatin licensing and DNA replication factor 1
DYL1	-1.79	3.93E-02	Dynein light chain 1 cytoplasmic
TMEM242	-1.80	1.94E-02	Transmembrane protein 242
PTN	-1.80	2.53E-02	Pleiotrophin
RFC3	-1.80	2.56E-02	Replication factor C subunit 3
ARHGAP18	-1.80	4.76E-05	Rho GTPase activating protein 18
PPP2R2C	-1.80	5.89E-04	Protein phosphatase 2, regulatory subunit B, gamma
HELLS	-1.81	6.85E-04	Helicase, lymphoid-specific
PHF21B	-1.81	7.83E-03	PHD finger protein 21B
SLC25A4	-1.82	6.09E-05	Solute carrier family 25, member 4
NDUFS1	-1.82	4.69E-04	NADH:ubiquinone oxidoreductase core subunit S1
HSP90AB1	-1.82	8.44E-05	Heat shock protein 90kDa alpha (cytosolic), class B member 1
DCLRE1C	-1.83	1.91E-02	DNA cross-link repair 1C
EI24	-1.83	3.22E-03	EI24, autophagy associated transmembrane protein
NTM	-1.83	2.37E-03	Neurotrimin
GNG13	-1.83	2.08E-02	Guanine nucleotide binding protein (G protein), gamma 13
CCDC125	-1.83	1.40E-02	Coiled-coil domain containing 125
MCAM	-1.84	2.92E-02	Melanoma cell adhesion molecule
PCNT	-1.84	3.23E-02	Pericentrin
PAPPA	-1.84	5.89E-05	Pregnancy-associated plasma protein A, pappalysin 1
PLD5	-1.85	1.96E-02	Phospholipase D family member 5
ROR2	-1.85	3.20E-02	Receptor tyrosine kinase-like orphan receptor 2
DNAH9	-1.85	4.69E-02	Dynein, axonemal, heavy chain 9
PPM1K	-1.86	2.82E-03	Protein phosphatase, Mg2+/Mn2+ dependent 1K
OSBPL10	-1.86	3.58E-02	Oxysterol binding protein-like 10
CCNG1	-1.87	6.74E-05	Cyclin G1
TENM4	-1.88	9.84E-04	Teneurin transmembrane protein 4
BRD3	-1.88	1.48E-03	Bromodomain containing 3
MTBP	-1.88	1.45E-03	MDM2 binding protein
GRIN3A	-1.89	3.03E-02	Glutamate receptor, ionotropic, N-methyl-D-aspartate 3A
NLE1	-1.89	3.06E-02	Notchless homolog 1 (Drosophila)
TCERG1L	-1.89	4.44E-02	Transcription elongation regulator 1 like
APOV1	-1.89	3.13E-02	Apovitellenin 1
UPP2	-1.90	3.76E-02	Uridine phosphorylase 2
TMEM56	-1.91	2.49E-02	Transmembrane protein 56

MCM3	-1.92	1.95E-04	Minichromosome maintenance complex component 3
FMOD	-1.92	1.66E-02	Fibromodulin
RNF182	-1.92	4.06E-02	Ring finger protein 182
CPT1A	-1.92	8.29E-05	Carnitine palmitoyltransferase 1A (liver)
TUBGCP4	-1.93	4.36E-02	Tubulin gamma complex associated protein 4
MDM1	-1.93	1.35E-02	Mdm1 nuclear protein
C14ORF80	-1.93	1.41E-03	Chromosome 8 open reading frame, human C14orf80
MYH15	-1.94	5.70E-05	Myosin, heavy chain 15
MMP16	-1.94	2.02E-03	Matrix metallopeptidase 16 (membrane-inserted)
PPARGC1A	-1.95	1.66E-04	PPARG coactivator 1 alpha
ANKRD31	-1.95	3.44E-02	Ankyrin repeat domain 31
BTBD11	-1.95	5.67E-03	BTB (POZ) domain containing 11
ART5	-1.95	4.17E-02	ADP-ribosyltransferase 5
GAB2	-1.97	1.87E-02	GRB2-associated binding protein 2
SLC5A9	-1.97	1.79E-02	Solute carrier family 5 (sodium/sugar cotransporter), member 9
AK1	-1.98	3.20E-05	Adenylate kinase 1
NIPAL4	-1.98	1.00E-04	NIPA-like domain containing 4
MCF2L2	-1.98	2.22E-02	MCF.2 cell line derived transforming sequence-like 2
DHRS11	-1.98	3.26E-02	Dehydrogenase/reductase (SDR family) member 11
TTLL3	-1.99	3.01E-02	Tubulin tyrosine ligase-like family member 3
HSPB7	-2.00	1.01E-02	Heat shock 27kDa protein family, member 7 (cardiovascular)
RAI2	-2.00	2.15E-03	Retinoic acid induced 2
ASL1	-2.01	1.56E-02	Argininosuccinate lyase
TMEM50B	-2.01	1.90E-02	Transmembrane protein 50B
NPTX2	-2.02	1.29E-04	Neuronal pentraxin II
BHLHE40	-2.02	3.01E-04	Basic helix-loop-helix family member e40
COL18A1	-2.02	3.06E-05	Collagen, type XVIII, alpha 1
ZWILCH	-2.03	2.77E-02	Zwilch kinetochore protein
SLC25A39	-2.03	3.30E-04	Solute carrier family 25, member 39
SRL	-2.03	8.23E-04	Sarcalumenin
ANXA10	-2.04	4.73E-02	Annexin A10
QPCT	-2.04	1.99E-03	Glutaminyl-peptide cyclotransferase
DNASE1L2	-2.05	1.20E-02	Deoxyribonuclease I-like 2
KLHL30	-2.06	1.44E-03	Kelch like family member 30
OPCML	-2.06	8.00E-04	Opioid binding protein/cell adhesion molecule-like
FOXA1	-2.06	2.48E-02	Forkhead box A1
TMX3	-2.07	8.72E-03	Thioredoxin related transmembrane protein 3
LRRK1	-2.08	1.68E-02	Leucine-rich repeat kinase 1
TOX	-2.09	4.92E-02	Thymocyte selection associated high mobility group box
CLEC3A	-2.09	4.52E-02	C-type lectin domain family 3 member A
RBP2	-2.09	1.26E-02	Retinol binding protein 2, cellular
ZDHHC5	-2.09	2.08E-03	Zinc finger, DHHC-type containing 5
CRB2	-2.10	2.56E-02	Crumbs family member 2
MYL2	-2.10	1.68E-02	Myosin, light chain 2, regulatory, cardiac, slow
MATN2	-2.11	3.76E-02	Matrilin 2
GSG1	-2.12	4.11E-03	Germ cell associated 1
BTF3	-2.13	2.01E-04	Basic transcription factor 3
PASK	-2.13	1.62E-02	PAS domain containing serine/threonine kinase
FAM160B1	-2.13	1.10E-02	Family with sequence similarity 160, member B1
ST3GAL3	-2.14	4.04E-02	ST3 beta-galactoside alpha-2,3-sialyltransferase 3
TEX33	-2.14	1.18E-02	Testis expressed 33
PDE10A	-2.15	5.00E-04	Phosphodiesterase 10A
UBE2E1	-2.15	1.22E-02	Ubiquitin-conjugating enzyme E2E 1 (UBC4/5 homolog, yeast)
ADORA2B	-2.15	1.21E-02	Adenosine A2b receptor
IGFBP2	-2.15	3.73E-03	Insulin like growth factor binding protein 2
PDK4	-2.16	1.90E-05	Pyruvate dehydrogenase kinase, isozyme 4

SPIDR	-2.17	7.25E-05	Scaffolding protein involved in DNA repair
MYH1D	-2.18	2.90E-02	Myosin, heavy chain 1D, skeletal muscle
B3GALT5	-2.18	2.98E-02	UDP-Gal:betaGlcNAc beta 1,3-galactosyltransferase, polypeptide 5
FP325317.1	-2.18	2.56E-04	Uncharacterized protein [Source:UniProtKB/TrEMBL;Acc:F6QWF9]
KIF26A	-2.18	4.97E-02	Kinesin family member 26A
RYR3	-2.19	4.23E-02	Ryanodine receptor 3
LRRC75B	-2.19	9.47E-03	Leucine rich repeat containing 75B
PROK1	-2.19	4.51E-03	Prokineticin 1
UBE2F	-2.20	4.16E-02	Ubiquitin-conjugating enzyme E2F (putative)
C12orf75	-2.20	1.48E-03	Chromosome 12 open reading frame 75
GPX7	-2.21	1.57E-02	Glutathione peroxidase 7
ABI3BP	-2.21	2.34E-04	ABI family member 3 binding protein
GPNMB	-2.22	4.34E-02	Glycoprotein nmb
IRX4	-2.22	4.49E-02	Iroquois homeobox 4
SGIP1	-2.23	2.81E-02	SH3-domain GRB2-like (endophilin) interacting protein 1
ARG2	-2.23	1.56E-02	Arginase 2
TSC22D3	-2.24	1.24E-02	TSC22 domain family member 3
SPATA17	-2.24	1.14E-02	Spermatogenesis associated 17
LCT	-2.25	7.41E-03	Lactase
GPC2	-2.26	4.68E-02	Glypican 2
PCYOX1L	-2.27	7.23E-03	Prenylcysteine oxidase 1 like
TNIP2	-2.27	6.75E-05	TNFAIP3 interacting protein 2
ADCY8	-2.28	5.07E-04	Adenylate cyclase 8 (brain)
MOGAT1	-2.29	1.54E-05	Monoacylglycerol O-acyltransferase 1
NALCN	-2.29	4.07E-02	Sodium leak channel, non-selective
ALDH1L2	-2.29	1.71E-02	Aldehyde dehydrogenase 1 family, member L2
ARHGAP32	-2.29	1.55E-02	Rho GTPase activating protein 32
KIF1BP	-2.29	2.66E-03	KIF1 binding protein
ENTPD1	-2.30	1.78E-05	Ectonucleoside triphosphate diphosphohydrolase 1
ZNF236	-2.30	2.65E-02	Zinc finger protein 236
ANK2	-2.30	7.28E-03	Ankyrin 2, neuronal
CBFB	-2.30	4.95E-02	Core-binding factor, beta subunit
FIGNL2	-2.30	8.78E-03	Fidgetin-like 2
DTNA	-2.31	2.58E-02	Dystrobrevin, alpha
PCYT1B	-2.31	2.26E-02	Phosphate cytidylyltransferase 1, choline, beta
FAM193B	-2.31	5.62E-03	Family with sequence similarity 193 member B
VANGL1	-2.31	3.90E-02	VANGL planar cell polarity protein 1
C1H12ORF63	-2.31	8.74E-03	Chromosome 1 open reading frame, human C12orf 63
COL2A1	-2.32	2.36E-02	Collagen, type II, alpha 1
RBP3	-2.32	1.30E-03	Retinol binding protein 3
HPCA	-2.33	2.30E-02	Hippocalcin
CYP1A4	-2.33	1.64E-04	Cytochrome P450 1A4
USP53	-2.33	8.35E-03	Ubiquitin specific peptidase 53
EPHA3	-2.34	2.95E-02	EPH receptor A3
PIP5K1B	-2.34	4.05E-02	Phosphatidylinositol-4-phosphate 5-kinase, type I, beta
PRPSAP1	-2.35	2.67E-02	Phosphoribosyl pyrophosphate synthetase-associated protein 1
CPLX1	-2.35	1.23E-02	Complexin 1
NOX4	-2.35	8.64E-03	NADPH oxidase 4
HSD17B3	-2.36	2.35E-02	Hydroxysteroid (17-beta) dehydrogenase 3
PLCZ1	-2.36	3.86E-02	Phospholipase C zeta 1
DNAJA2	-2.37	3.03E-02	DnaJ heat shock protein family (Hsp40) member A2
FAR1	-2.37	1.81E-02	Fatty acyl CoA reductase 1
GIGYF2	-2.37	4.03E-02	GRB10 interacting GYF protein 2
CHRNA7	-2.37	9.45E-03	Cholinergic receptor, nicotinic, alpha 7 (neuronal)
AMIGO2	-2.37	9.12E-03	Adhesion molecule with Ig-like domain 2
GULP1	-2.38	3.44E-02	GULP, engulfment adaptor PTB domain containing 1

PID1	-2.39	2.52E-04	Phosphotyrosine interaction domain containing 1
TBC1D23	-2.39	2.25E-02	TBC1 domain family member 23
PIM3	-2.40	4.82E-04	Pim-3 oncogene
SLC16A6	-2.41	4.13E-02	Solute carrier family 16, member 6
SEZ6L	-2.41	2.50E-02	Seizure related 6 homolog (mouse)-like
CKAP2L	-2.41	5.49E-03	Cytoskeleton associated protein 2 like
SF3B3	-2.42	1.08E-03	Splicing factor 3b subunit 3
RASGEF1A	-2.43	1.51E-04	RasGEF domain family, member 1A
HSP25	-2.43	3.23E-03	Heat shock protein 25
PRDM6	-2.43	4.74E-02	PR domain 6
MAPKBP1	-2.44	9.24E-03	Mitogen-activated protein kinase binding protein 1
LSM8	-2.48	8.57E-04	LSM8 homolog, U6 small nuclear RNA associated (S. Cerevisiae)
SLC35A1	-2.48	2.11E-03	Solute carrier family 35 (CMP-sialic acid transporter), member A1
SELP	-2.48	8.83E-03	Selectin P (granule membrane protein 140kDa, antigen CD62)
TIPRL	-2.48	2.62E-02	TOR signaling pathway regulator
HAS3	-2.48	3.49E-03	Hyaluronan synthase 3
NSUN6	-2.49	3.71E-02	NOP2/Sun RNA methyltransferase family member 6
PPP1R1B	-2.49	7.97E-04	Protein phosphatase 1, regulatory (inhibitor) subunit 1B
CFAP58	-2.50	4.35E-03	Cilia and flagella associated protein 58
SLC45A3	-2.50	1.06E-03	Solute carrier family 45 member 3
FKBP5	-2.52	3.68E-05	FK506 binding protein 5
PTPRS	-2.53	5.22E-03	Protein tyrosine phosphatase, receptor type, S
POLN	-2.53	3.07E-02	Polymerase (DNA) nu
MYO16	-2.53	3.81E-02	Myosin XVI
RNF144A	-2.53	7.42E-05	Ring finger protein 144A
SLC26A7	-2.53	1.40E-03	Solute carrier family 26 (anion exchanger), member 7
ZFHX3	-2.54	1.28E-02	Zinc finger homeobox 3
AVDL	-2.54	1.49E-04	Avidin-like
TUSC5	-2.55	1.01E-03	Tumor suppressor candidate 5
SPTBN5	-2.56	8.21E-03	Spectrin, beta, non-erythrocytic 5
HTR1A	-2.57	1.55E-02	5-hydroxytryptamine receptor 1A
CHODL	-2.57	2.52E-04	Chondrolectin
MAGI2	-2.57	1.60E-02	Membrane associated guanylate kinase, WW and PDZ domain containing
ZPLD1	-2.58	1.83E-02	Zona pellucida-like domain containing 1
TMEM108	-2.58	1.32E-02	Transmembrane protein 108
SLC7A9	-2.59	8.12E-03	Solute carrier family 7, member 9
RBM22	-2.60	6.08E-03	RNA binding motif protein 22
NIN	-2.60	3.39E-02	Ninein
GPR85	-2.61	3.65E-02	G protein-coupled receptor 85
ABLIM1	-2.61	6.59E-03	Actin binding LIM protein 1
MYH1E	-2.61	1.58E-02	Myosin, heavy chain 1E, skeletal muscle
FBXO40	-2.62	3.31E-03	F-box protein 40
UNG	-2.62	4.87E-04	Uracil-DNA glycosylase
SDCCAG3	-2.63	1.83E-05	Serologically defined colon cancer antigen 3
SPOCK1	-2.64	1.49E-02	Sparc/osteonectin, cwcv and kazal-like domains proteoglycan (testican) 1
FUBP3	-2.65	8.12E-05	Far upstream element (FUSE) binding protein 3
RANBP9	-2.67	1.21E-02	RAN binding protein 9
LRRN3	-2.67	1.06E-03	Leucine rich repeat neuronal 3
KCTD8	-2.68	4.42E-02	Potassium channel tetramerization domain containing 8
CNR1	-2.69	2.81E-02	Cannabinoid receptor 1 (brain)
CAPN9	-2.69	4.15E-02	Calpain 9
RIMS3	-2.70	1.07E-03	Regulating synaptic membrane exocytosis 3
DAB1	-2.71	1.13E-04	Dab, reelin signal transducer, homolog 1 (Drosophila)
OR5AS1	-2.71	4.60E-03	Olfactory receptor, family 5, subfamily AS, member 1
PPP2R2D	-2.72	2.72E-02	Protein phosphatase 2, regulatory subunit B, delta
SCRN1	-2.72	1.48E-03	Secernin 1

PI4KB	-2.72	1.87E-02	Phosphatidylinositol 4-kinase, catalytic, beta
AvBD7	-2.73	4.84E-04	Avian beta-defensin 7
FAM38B	-2.73	2.97E-02	Family with sequence similarity 38, member B
CEP83	-2.73	1.14E-02	Centrosomal protein 83kDa
FAM189A1	-2.74	7.19E-05	Family with sequence similarity 189 member A1
SH3BGR	-2.76	1.50E-04	SH3 domain binding glutamate rich protein
TNN	-2.76	1.40E-03	Tenascin N
PHC3	-2.80	2.47E-02	Polyhomeotic homolog 3
SWAP70	-2.80	3.01E-02	SWAP switching B-cell complex 70kDa subunit
HSPH1	-2.81	1.04E-02	Heat shock 105kDa/110kDa protein 1
GPR17	-2.83	3.13E-02	G protein-coupled receptor 17
BNC1	-2.84	1.63E-02	Basonuclin 1
GNG4	-2.84	1.89E-05	Guanine nucleotide binding protein (G protein), gamma 4
CCDC122	-2.86	3.46E-03	Coiled-coil domain containing 122
BIRC7	-2.88	2.62E-02	Baculoviral IAP repeat containing 7
AvBD2	-2.88	1.01E-04	Avian beta-defensin 2
TTLL9	-2.89	3.32E-03	Tubulin tyrosine ligase like 9
PRDM16	-2.89	2.63E-03	PR domain containing 16
IGFBP1	-2.91	2.12E-05	Insulin like growth factor binding protein 1
DELTARB1	-2.92	8.75E-03	Variant retinoblastoma transcription factor
FAM105A	-2.92	3.51E-05	Family with sequence similarity 105, member A
RD3L	-2.92	6.23E-04	Retinal degeneration 3-like
ACBD7	-2.93	4.94E-04	Acyl-CoA binding domain containing 7
STMN3	-2.93	4.15E-02	Stathmin-like 3
SERPINI1	-2.94	4.71E-02	Serpin peptidase inhibitor, clade I (neuroserpin), member 1
SPNS2	-2.95	2.32E-04	Spinster homolog 2 (Drosophila)
ATXN7L1	-2.95	5.99E-04	Ataxin 7-like 1
MYO1H	-2.96	4.70E-05	Myosin IH
S100A9	-2.97	1.81E-04	S100 calcium binding protein A9
ITGBL1	-2.97	5.47E-04	Integrin subunit beta like 1
KCNJ5	-2.97	2.81E-02	Potassium inwardly-rectifying channel, subfamily J, member 5
SLC24A1	-2.98	6.98E-05	Solute carrier family 24 (sodium/potassium/calcium exchanger), member [illegible]
TMEM259	-2.99	2.75E-02	Transmembrane protein 259
COL23A1	-2.99	2.17E-02	Collagen, type XXIII, alpha 1
CDHR5	-2.99	1.29E-02	Cadherin-related family member 5
CHD2	-3.01	2.33E-02	Chromodomain helicase DNA binding protein 2
STOML2	-3.02	3.62E-03	Stomatin like 2
SYT1	-3.04	1.05E-02	Synaptotagmin I
AvBD6	-3.07	1.48E-03	Avian beta-defensin 6
CREBBP	-3.08	5.39E-05	CREB binding protein
GRIA4	-3.09	2.86E-03	Glutamate receptor, ionotropic, AMPA 4
PDE1C	-3.09	2.40E-02	Phosphodiesterase 1C, calmodulin-dependent 70kDa
CHRDL1	-3.09	2.40E-02	Chordin-like 1
ZHX3	-3.09	7.09E-03	Zinc fingers and homeoboxes 3
CXCL14	-3.09	2.60E-03	C-X-C motif chemokine ligand 14
SERPINB10	-3.12	1.38E-04	Serpin peptidase inhibitor, clade B (ovalbumin), member 10
LIPC	-3.12	5.51E-05	Lipase C, hepatic type
CATH1	-3.13	2.63E-04	Cathelicidin-1
SIX4	-3.15	2.11E-05	SIX homeobox 4
NTRK2	-3.15	4.26E-04	Neurotrophic tyrosine kinase, receptor, type 2
ARHGAP26	-3.17	2.14E-02	Rho GTPase activating protein 26
SCML2	-3.17	4.35E-03	Sex comb on midleg-like 2 (Drosophila)
DLG2	-3.17	3.32E-03	Discs, large homolog 2 (Drosophila)
TALDO1	-3.19	1.86E-03	Transaldolase 1
NAF1	-3.22	6.83E-04	Nuclear assembly factor 1 ribonucleoprotein
AvBD1	-3.24	1.97E-04	Avian beta-defensin 1

TMTC1	-3.26	1.07E-04	Transmembrane and tetratricopeptide repeat containing 1
CASC5	-3.28	6.27E-03	Cancer susceptibility candidate 5
ZNF518B	-3.31	3.94E-03	Zinc finger protein 518B
LECT2	-3.31	1.11E-04	Leukocyte cell derived chemotaxin 2
FBXO22	-3.33	3.33E-03	F-box protein 22
FST	-3.34	9.57E-04	Follistatin
MMP9	-3.35	1.07E-03	Matrix metallopeptidase 9
CDON	-3.39	3.55E-05	Cell adhesion associated, oncogene regulated
UAP1L1	-3.41	3.73E-02	UDP-N-acteylglucosamine pyrophosphorylase 1-like 1
LINGO1	-3.42	3.04E-05	Leucine rich repeat and Ig domain containing 1
MALL	-3.44	2.16E-03	Mal, T-cell differentiation protein-like
GAL3ST2	-3.54	1.94E-02	Galactose-3-O-sulfotransferase 2
ACACB	-3.54	2.46E-03	Acetyl-CoA carboxylase beta
CNNM2	-3.54	3.17E-03	Cyclin M2
FGF12	-3.58	6.47E-04	Fibroblast growth factor 12
AvBD4	-3.59	3.66E-03	Avian beta-defensin 4
CYGB	-3.67	6.50E-04	Cytoglobin
SSX2IP	-3.68	1.07E-05	Synovial sarcoma, X breakpoint 2 interacting protein
ALX1	-3.70	1.23E-03	ALX homeobox 1
HOXB6	-3.75	3.69E-05	Homeobox B6
CHAC1	-3.85	5.53E-06	ChaC glutathione-specific gamma-glutamylcyclotransferase 1
C1QTNF9	-4.08	1.22E-04	C1q and tumor necrosis factor related protein 9
VSTM2A	-4.09	2.33E-04	V-set and transmembrane domain containing 2A
CTRC	-4.11	4.26E-02	Chymotrypsin C (caldecrin)
ZBTB16	-4.12	2.98E-04	Zinc finger and BTB domain containing 16
DIO3	-4.13	1.33E-02	Deiodinase, iodothyronine, type III
LHFPL5	-4.14	4.82E-05	Lipoma HMGIC fusion partner-like 5
FABP2	-4.58	4.93E-02	Fatty acid binding protein 2
GCG	-4.59	3.06E-02	Glucagon

Supplementary file 2. GO enrichment analysis for upregulated DEGs found in the liver of broilers at D7 compared to D0					
**Term**	**Enrichment**	**PValue**	**Count**	**Garoup**	**Genes**
Immune response	2.69	3.11E-06	29	BP	NRROS, HHLA2, CST7, CTSS, TNFSF13B, RXFP3, ENPP2, CCR9, DMA, TLR21, CCR7, CCL1, B2M, CD74, TNFSF15, IL15, BLB1, IL13, LIF, OPRK1, TLR2A, PDCD1LG2, BF2, CD40LG, IGLL1, CD28, FAS, XCL1, TLR3
Innate immune response	2.23	2.08E-03	19	BP	LYN, C1QA, SPON2, FCER1G, TLR2A, ATG14, WFDC2, JCHAIN, LCK, NEDD4, HKDC1, SARM1, BTK, TRIM14, ANKS6, TLR21, SERINC3, TLR3, ERK
Inflammatory response	1.90	1.39E-02	18	BP	PARP4, NRROS, TLR2A, P2RX7, CRHBP, CD40LG, ELF3, HKDC1, IL2RA, FASN, HYAL3, C3AR1, XCL1, TRIM14, TLR21, CCL1, CCR7, TLR3
Positive regulation of ERK1 and ERK2 cascade	2.20	5.95E-03	16	BP	CSF1R, CD74, TPBG, OSGIN1, IGF1, TNFRSF11A, DUSP15, P2RY6, PTPRC, GLIPR2, TRPV4, GPR183, F2RL1, XCL1, CCL1,
Nervous system development	2.02	1.27E-02	16	BP	SEMA5A, FMR1, SOX11, PAX5, NAV2, PAX2, NEUROD4, BRINP1, NAV3, UNC119, CSPG5, DPF3, HMCN1, SIM1, AGRN, DSCAML1
Negative regulation of gene expression	1.87	2.42E-02	16	BP	EPM2A, KDM4A, SPI1, AXIN1, ZBTB20, SOX11, PARP14, PARP9, CD3E, PARK2, TP53INP1, TBX20, CD28, ROS1, XDH, WNT4
Axon guidance	2.14	1.01E-02	15	BP	SEMA5A, CSF1R, TRIO, WNT3A, LAMA1, SEMA3E, EFNA5, ROBO1, PRTG, GBX2, KIF5C, PLA2G10, NRCAM, DSCAML1, MEGF9
Cholesterol biosynthetic process	11.83	8.97E-12	14	BP	FDPS, MVK, INSIG1, PMVK, MVD, DHCR7, HMGCR, HSD17B7, A4GALT, LSS, LIPA, FDFT1
Positive regulation of peptidyl-tyrosine phosphorylation	4.91	3.21E-06	14	BP	CD74, IL15, LIF, LRP4, IGF1, EFNA5, CD3E, KITLG, PTPRC, BANK1, NTF3, PECAM1, ENPP2,
Positive regulation of phosphatidylinositol 3-kinase/protein kinase B signal	2.03	2.62E-02	13	BP	CSF1R, TCF7L2, FLT1, TPBG, INSRR, SEMA3E, IGF1, RTN4, PIK3R5, NEDD4, CD28, F2RL1, HCLS1
Defense response to virus	2.32	1.38E-02	12	BP	SPON2, PTPRC, ZNFX1, GPAM, IRF1, PLA2G10, OPRK1, SERINC3, ATG14, DDX60, ATG7, TLR3
Positive regulation of MAPK cascade	1.92	4.74E-02	12	BP	LYN, P2RX7, FLT1, AVPR1B, BANK1, IGFBP4, PRKCE, INSRR, LIF, FGF23, GRM1, WNT4

Cholesterol homeostasis	2.92	4.03E-03	11	BP	CYP39A1, ERRFI1, GRAMD1B, INSIG1, MTTP, PLA2G10, ANGPTL3, HDAC9, CYP7A1,
Positive regulation of GTPase activity	2.84	4.95E-03	11	BP	DOCK10, DOCK11, DOCK8, DOCK7, ARAP2, ARAP3, XCL1, CCL1, ADAP1L, AGRN, RALGPS1
Regulation of apoptotic process	2.04	4.22E-02	11	BP	CARD10, PINK1, TRAF5, NTF3, ABL2, FAS, BAK1, IFT57, CARD11, BCL2L14, PARK2
Fatty acid metabolic process	3.05	5.02E-03	10	BP	GPAM, UCP3, NAAA, ANGPTL3, AOAH, LIPA, ACSBG2, ACADSB, ACSF2, AACS
T cell receptor signaling pathway	2.82	8.45E-03	10	BP	PTPRC, LCK, ZC3H12A, BTK, CD28, LCP2, CD247, CD3E, ICOSLG, TNFRSF21
Phospholipase C-activating G protein-coupled receptor signaling	2.38	2.36E-02	10	BP	P2RY6, AVPR1B, C3AR1, ABL2, F2RL1, DGKK, PLCB2, DGKZ, HCRTR2, RXFP3
Immune system process	5.40	1.75E-04	9	BP	FCER1G, RNF213, IRF4, IRF1, IL2RA, INPP5D, SH2D1B, CD3E,
Chemotaxis	4.65	5.30E-04	9	BP	FLT1, FES, C3AR1, CCR9, RAC2, PROK2, IL16, CCR7, DOCK2
Fatty acid biosynthetic process	3.22	6.18E-03	9	BP	FADS2, ACLY, ELOVL5, SCD, FASN, ELOVL2, ELOVL6, FADS1, ACACA
Cholesterol metabolic process	3.16	6.95E-03	9	BP	CYP39A1, ERRFI1, SQLE, NSDHL, CYP11A1, INSIG1, ANGPTL3, ABCG1, SREBF2
Cellular response to starvation	2.61	2.08E-02	9	BP	KLF10, INHBB, WIPI1, ATG14, ATG7, TNRC6A, SREBF2, MFSD2A, WNT4
Positive regulation of interleukin-6 production	3.10	1.36E-02	8	BP	P2RX7, CD74, SPON2, TRPV4, ZBTB20, F2RL1, TLR3, EREG
Positive regulation of B cell	3.94	7.53E-03	7	BP	CD74, BST1, PTPRC, WNT3A, GPR183, CARD11, TNFSF13B
Negative thymic T cell selection	13.94	2.16E-05	6	BP	CD74, PTPRC, CD28, CCR7, CD3E, DOCK2
Unsaturated fatty acid biosynthetic process	10.14	1.56E-04	6	BP	FADS2, ELOVL5, SCD, ELOVL2, ELOVL6, FADS1
T cell costimulation	7.43	8.46E-04	6	BP	HHLA2, CD28, PDCD1LG2, CD3E, ICOS, CARD11
Positive regulation of glycolytic	6.97	1.18E-03	6	BP	P2RX7, KAT2B, UCHL1, GPD1, ZBTB20, IGF1
Positive regulation of T cell	3.98	1.55E-02	6	BP	KITLG, HHLA2, IL15, CD28, PDCD1LG2, HES4
Apoptotic signaling pathway	3.60	2.35E-02	6	BP	DIDO1, IFI27L2, CD28, INHBB, CD3E, WNT4
Circadian rhythm	3.48	2.67E-02	6	BP	KLF10, HTR7, MTTP, CRY2, PROK2, ASS1
Regulation of cytokine production	3.28	3.38E-02	6	BP	LYN, GPAM, ZBTB25, ZBTB20, LITAF, ICOSLG

Positive thymic T cell selection	9.29	1.34E-03	5	BP	CD74, PTPRC, CD3E, DOCK2, CD3D
Negative regulation of non-canonical NF-kappaB signal transduction	5.47	1.12E-02	5	BP	ADGRG3, SPI1, RASSF2, ZC3H12A, LITAF
Antigen processing and presentation of exogenous peptide antigen via MHC class II	5.47	1.12E-02	5	BP	CD74, IGLL1, BLB1, DMA, B2M
Sensory perception of pain	5.47	1.12E-02	5	BP	P2RX7, NPY1R, OPRK1, GRM1, PTGES
Protein targeting to lysosome	5.16	1.38E-02	5	BP	GGA3, NEDD4, M6PR, VPS54, SORL1
Regulation of mitochondrial membrane potential	4.65	2.01E-02	5	BP	PINK1, BCO2, BAK1, LIPA, PARK2
Calcium ion import across plasma membrane	4.22	2.79E-02	5	BP	TRPM1, SLC8A3, SLC24A4, TRPV6, TRPV4
Positive regulation of T cell activation	4.04	3.24E-02	5	BP	IGLL1, LCK, BLB1, DMA, B2M
Extrinsic apoptotic signaling pathway via death domain receptors	4.04	3.24E-02	5	BP	ITPRIP, FAS, PIK3R1, NGF, SKIL
Response to antibiotic	3.87	3.73E-02	5	BP	CYB5R4, CYP1A2, HYAL3, VPS54, ALPL
Positive regulation of interleukin-2 production	3.87	3.73E-02	5	BP	PTPRC, IRF4, CD28, CD3E, CARD11
Calcium ion homeostasis	3.72	4.26E-02	5	BP	KL, TRPV6, ALPL, ITPR3, FGF23
Response to calcium ion	3.57	4.83E-02	5	BP	P2RX7, TRPV6, KCNMA1, VPS54, ITPR3
Negative regulation of B cell	7.43	1.40E-02	4	BP	LYN, INPP5D, BTK, TNFRSF21
Isoprenoid biosynthetic process	7.43	1.40E-02	4	BP	FDPS, MVK, MVD, HMGCR
Negative regulation of amyloid-beta formation	7.43	1.40E-02	4	BP	GGA3, IGF1, SORL1, RTN4
Immunoglobulin production involved in immunoglobulin-mediated immune response	6.76	1.84E-02	4	BP	IGLL1, BLB1, DMA, B2M
Peptide antigen assembly with MHC class II protein complex	6.76	1.84E-02	4	BP	IGLL1, BLB1, DMA, B2M
Positive regulation of immune response	6.76	1.84E-02	4	BP	IL15, BLB1, DMA, B2M

Phospholipid homeostasis	5.72	2.95E-02	4	BP	GPAM, ANGPTL3, TLCD1, ABCG1
Negative regulation of neurogenesis	5.31	3.61E-02	4	BP	PRTG, BRINP1, SORL1, B2M
Kidney morphogenesis	4.96	4.34E-02	4	BP	SALL1, GCNT1, PAX2, WNT4
Triterpenoid biosynthetic process	18.58	8.34E-03	3	BP	LSS
Negative regulation of vascular endothelial cell proliferation	18.58	8.34E-03	3	BP	MEF2C, FLT1, COL4A3
Cellular response to hyperoxia	11.15	2.59E-02	3	BP	FAS, ATG7, NOX1
Positive regulation of prostaglandin secretion	11.15	2.59E-02	3	BP	P2RX7, PLA2G10, PTGES
Regulation of cellular response to oxidative stress	9.29	3.74E-02	3	BP	PINK1, SLC7A11, PARK2
Skeletal muscle acetylcholine-gated channel clustering	9.29	3.74E-02	3	BP	LRP4, AGRN, RAPSN
Membrane	1.19	1.37E-03	236	CC	IFI27L2, TRAF3IP3, PLEKHB1, LDLRAD3, CWH43, SOGA1, RXFP3, NSDHL, HERC2, ICOSLG, MFSD2A, TMEM52B, TIMD4, KCNK17, TM4SF4, SLC51A, EREG, CYP39A1, TIAM2, MYL1, ADPRH, FREM2, PTGES, SMIM3, MACF1, TMEM161B, TMEM63A, IGSF3, SLC45A1, HHLA2, SLC22A2, MGST3, UMOD, CD1C, PPL, ACMSD, TRPM1, RAB11FIP1, RNF213, TSPAN8, TTPA, KCNMB1, AGMO, INPP5D, ARSJ, TLR21, TRPM8, ICOS, SNX8, FDFT1, STARD13, TNFSF15, GREB1, SUCO, TLR2A, IGF1, FER1L6, MARCO, ACER1, BST1, SQLE, SLC6A7, GLIPR1L, GPR183, CD28, PLCH1, TMCC2, FBXL2, SLC22A7, NOX1, PIGR, TENM3, SLC7A14, ARHGAP1, TMEM181, SLC7A11, LRRC32, CD3E, CD3D, ROBO1, BRS3, CCKAR, KCNT2, CCR9, FLVCR2, CCR7, FCER1G, GPR34, AVPR1B, ELOVL5, NFAM1, ELOVL2, INSRR, ELOVL6, PAX2,

Plasma membrane	1.20	3.02E-03	191	CC	SEMA5A, ERRFI1, ITPRIP, STEAP4, NPY2R, C2ORF88, SLC4A1, LDLRAD3, LAPTM4B, MFSD13A, GJA1, ALCAM, HTR7, HERC2, RASSF2, BASP1, C3AR1, NRCAM, HMCN1, ROS1, MFSD2A, FNBP1, KCNH7, PRKCE, ARRDC3, CD180, ANXA13, NPY1R, SOX11, SLC51A, SLC9A2, ACE2, SCNN1G, SCN8A, MAP1B, SIDT1, BTK, SLC27A6, IGFALS, DGKK, CARD11, ABCG1, MACF1, CSF1R, ENPEP, CALCRL, TMEM63A, SLC41A2, PCDH15, CHRNA8, DAPP1, ITPR3, EFNA5, PDS5A, LYPD6, PCDH17, PIK3R5, CACNA1G, ADGRG3, PKHD1, DPP4, TSPAN8, CDH20, CDH22, LPXN, NTSR1, LYN, ATP8B3, FZD5, WNT3A, TNFSF15, HTR1D, MICAL3, OSGIN1, TLR2A, CP, BST1, BFSP1, SCCPDH, TRPV6, FASN, TRPV4, GPR183, SLCO1A2, SPIRE2, CD28, FAT2, ALPL, CSPG5, CNTN3, SERINC3, CDK14, PLEKHM3, NOX1, PIGR, ACHE,
Cytosol	1.27	7.15E-04	167	CC	ERRFI1, PANK3, FMR1, WWC2, MAP3K7CL, DIXDC1, HNMT, LIPA, ENO3, PARK2, NR3C2, MYLK, HID1, GLE1, RGS5, BAIAP2L2, HERC2, RASSF2, ANKS1A, B2M, SCAPER, FAAP100, EPM2A, STARD4, PRKCE, TBC1D2B, DGKZ, RUNDC3A, ACLY, PPP1R3C, MAP1B, ZNF318, GPD1, BTK, FSCN1, PPEF2, CARD11, MAOA, MTTP, FAM109B, TNFRSF11A, PIK3R1, DDX60, ACACA, PIK3R5, ACMSD, RAB11FIP1, RNF213, INPP5D, AFMID, PMVK, LPXN, TPPP, SNX8, FDPS, CBX7, WNT3A, MICAL3, LIF, DCDC2, INO80, HPS5, STAM, GCLC, BPIFB4, PQLC1, FASN, PLCH1, GTF2IRD1, CEP85, BRWD1, CDK14, KDM5B, C2CD5, ZCCHC11, NUMA1, DOCK8, GBE1, SLC7A14, PIK3CD, DUSP15, AACS, ANKRD9, UCHL1, CCKAR, XPO4, GM2A, VCPKMT, CEP170, CYTIP, CEP290, UNC45A, PARP4, TSC22D1, IL15, PDE4C,

Extracellular space	1.73	4.64E-07	88	CC	CRISP2, PCSK1, ACHE, SPON2, NRROS, LOXL4, DBH, LRRC32, SOGA1, CTSS, TNFSF13B, CRHBP, C1QTNF8, GLIPR2, ENPP2, TIMP2, CA6, FAM3D, TIMP4, HABP2, KRT6A, SPINK4, CHIA, SPINK7, GPX3, IGFBP4, IL15, CD180, IL16, NGF, EREG, SLCO1C1, ACE2, TMEFF1, SLCO4A1, PLA2G10, ANGPTL3, PECAM1, XCL1, ANGPTL4, LRRC3B, SMPDL3A, WNT11B, LXN, GRP, LAMA1, OVSTL, FGL2, UMOD, SEMA3E, AFP, CST7, FSTL4, CPN2, SCUBE2, PRRG4, HPX, CLEC3B, AVBD10, NTF3, SPP1, CCL1, C3ORF33, AVBD8, FGF23, XDH, WNT4, S100A10, WNT3A, TNFSF15, GZMA, LIF, INHBB, IGF1, SORL1, LRRC66, CP, A2ML4, BF2, KITLG, CD40LG, GLIPR1L, IGLL1, SMOC1,
Extracellular region	1.37	6.60E-03	69	CC	PIGR, CRISP2, IL22, SPON2, NRROS, FLT1, LEAP2, CWH43, CTSS, WFDC2, CRHBP, GLIPR2, ENPP2, TIMP2, CA6, PROK2, HMCN1, EDIL3, B2M, TIMP4, HABP2, CHIA, IGFBP4, IL15, IL13, NGF, EREG, AVD, ACE2, COL4A4, PLA2G10, CRY2, COL4A3, XCL1, DNASE1, C1QA, WNT11B, LAMA1, OVSTL, COCH, NTN3, SCUBE2, PRRG4, DPP4, HPX, AVBD10, WIF1, ADAMTS18, NTF3, SPP1, CCL1, AVBD8, WNT4, CD74, WNT3A, LIF, GUCA2B, PRSS35, INHBB, IGF1, SORL1, A2ML4,
Endoplasmic reticulum	1.50	5.17E-03	48	CC	LRRC59, NRROS, CALCRL, MGST3, INSIG1, MTTP, ITPR3, ZDHHC21, ATP2A1, DBH, CWH43, CST7, CD3E, PARK2, PKHD1, TRPM1, CYB5R4, NSDHL, AGMO, HYAL3, AGR3, BAK1, SEC31B, FDFT1, CD74, STARD4, WNT3A, ELOVL5, SPINK7, TPBG, PRKCE, ELOVL2, DHCR24, ELOVL6, SIRT6, KCNRG, SORL1, MTTPL, SQLE, ACER1, TSPO2, PINK1, [illegible]

Endoplasmic reticulum membrane	1.39	2.96E-02	42	CC	LRRC59, GRAMD1B, INSIG1, ITPR3, MSMO1, ATP2A1, HMGCR, TMEM260, PTGS2, HSD17B7, CYP7A1, RTN4, UCHL1, AGMO, FLVCR2, SC5D, SPTSSB, A4GALT, FDFT1, MFSD2A, RHBDF1, CERS4, STARD3NL, ELOVL5, ELOVL2, TAP2, ORMDL1, ELOVL6, SLC51A, LSS, SORL1, TMEM174, SREBF2, SLC8A3, CYP39A1, SQLE, SCD, CYP1A2, SLC27A6, DHCR7
Cell surface	1.63	1.78E-02	26	CC	CSF1R, NRROS, ITGB2, LRP4, UMOD, SLC7A11, ROBO1, SCUBE2, TSPAN8, WIF1, CCR9, ROS1, WNT4, CD74, FCER1G, WNT3A, SORL1, ACE2, CYP2W2, CD40LG, ANGPTL3, SARM1, F2RL1, FAS, CDH17, DSCAML1
External side of plasma membrane	2.07	1.75E-03	23	CC	CD74, ENPEP, HHLA2, TNFRSF18, TNFRSF11A, PDCD1LG2, EFNA5, CD3E, TARP, CD3D, BF2, ENOX1, P2RX7, SCNN1G, PTPRC, ALCAM, CD28, FAS, CCR7, TRPM8, ABCG1, ICOSLG, CD244
Axon	1.75	2.45E-02	19	CC	KCNC2, FMR1, INSRR, CHRNA8, NGF, ROBO1, SLC8A3, PRTG, PINK1, ALCAM, SCN8A, FEZ1, NTF3, ALDH1A1, SCN4A, NRCAM, HMCN1, AGRN, DSCAML1
Dendrite	1.73	2.76E-02	19	CC	EPM2A, ELOVL5, KCNC2, TPBG, HTR1D, CHRNA8, SORBS2, NGF, GRM1, GCHFR, HTR7, ALCAM, BRINP1, MAP1B, NTF3, SARM1, GNB3, CPEB4, MARK1
Apical plasma membrane	1.66	3.88E-02	19	CC	ENPEP, SLC24A4, SLC31A1, SLC22A2, SLC5A11, UMOD, SHROOM1, TMEM174, SLC9A2, PKHD1, DPP4, SCNN1G, ACE2, GJA1, SLC9A4, PARD6B, TRPV4, KCNMA1, SLC22A7
Membrane raft	2.28	1.16E-02	13	CC	LYN, TNFRSF11A, DPP4, GRIP1, PTPRC, LCK, BTK, MAL, FAS, TRPM8, AGRN, NTSR1, CARD11
Basement membrane	2.74	1.03E-02	10	CC	LAMA1, COL4A4, SMOC1, COL4A3, HMCN1, EFNA5, AGRN, FREM2, MEGF9, NTN3
Centriolar satellite	2.13	4.51E-02	10	CC	C2CD5, TMEM63A, CYTH4, C2CD3, RASSF7, DCDC2, DBH, CEP290, PAX2, PIK3R5
Neuromuscular junction	2.80	2.28E-02	8	CC	SLC8A3, P2RX7, FCHSD2, SARM1, LRP4, SYNGR3, AGRN, RAPSN
Microtubule organizing center	3.16	3.95E-02	6	CC	CCNE1, TEKT2, DCDC2, MAPRE2, CLASP1, CLASP2
MHC class II protein complex	6.25	6.96E-03	5	CC	CD74, IGLL1, BLB1, DMA, B2M

Immunological synapse	3.70	4.35E-02	5	CC	ALCAM, LCK, CD28, CD3E, CARD11
Myosin II complex	5.71	3.00E-02	4	CC	MYL4, MYL1, TNNC1, MYL3
Alpha-beta T cell receptor complex	14.99	1.40E-02	3	CC	CD247, CD3E, CD3D
Protein binding	1.21	1.51E-02	126	MF	TRIO, NRROS, ZBTB25, FBXO25, DIXDC1, LRRC72, ZBTB20, LDLRAD3, FBXO21, BACH1, MYLK, BAIAP2L2, MAP3K9, ANKS1A, ROS1, SAMD10, SPINK4, ADGB, TLE3, CARD10, FNBP1, SPINK7, CD180, IFT80, DGKZ, GRIP2, TANC2, TIAM2, SCN8A, BTK, TRIM14, ANKS6, IGFALS, DGKK, WDFY4, CARD11, LRRC59, CREM, TRANK1, PIK3R1, SAMSN1, CASKIN2, CMYA5, ABL2, TLR21, LONRF1, MAP2K5, SEC31B, KLHL25, MICAL3, TLR2A, HPS5, LRRC66, LRCH2, PTPRC, TADA2A, TRPV6, TRPV4, SLCO1A2, GNB3, FBXL2, BRWD1, EIF4G3, KCNC2, CHD6, WDR41, LRRC32, ROBO1, ANKRD9, KBTBD12, C1QTNF8, TRIM3, CEP170, CYTIP, TNFRSF18, ARHGEF16, ARAP2, LRRC40, ARAP3, WIPI1, NAV2, SLCO1C1, TMEFF1, KAT2B, ASPM, FCHSD2, NAV3, SLCO4A1, IRF4, LCP2, SCN4A, LRRC3B, L3MBTL4, TLR3
Protein homodimerization activity	1.57	4.38E-03	42	MF	CSF1R, TENM3, PANK3, FMR1, BHLHE41, LRP4, FAM109B, SLC4A1, CST7, MFF, PTGS2, CLCN1, JCHAIN, NADK2, GLIPR2, TPPP, BAK1, CRYM, ATG7, B2M, SKIL, XDH, HES4, EPM2A, FCER1G, STARD3NL, TNNC1, GZMA, AXIN1, INHBB, SIRT6, SLC51A, S100Z, GPD1, ZNF318, PECAM1, MVP, COL9A3, CD247
Actin binding	1.61	4.34E-02	20	MF	CAP1, MACF1, WIPF3, MICAL3, LMO7, LDB3, LRRC10, PARK2, NCALD, LMOD3, INF2, ABLIM2, MAP1B, KCNMA1, SPIRE2, LPXN, MYOZ3, EVL, DNASE1, MYO1F
Oxidoreductase activity	2.04	7.27E-03	18	MF	MAOB, ADH1C, MAOA, LOXL4, MSMO1, ETFA, CP, ENOX1, FADS2, CYB5R4, SCCPDH, FASN, AGMO, SARDH, SC5D, A4GALT, FADS1, NOX1

Guanyl-nucleotide exchange factor activity	1.89	1.45E-02	18	MF	PSD, TRIO, DOCK8, DOCK7, ARHGEF16, WDR41, DOCK10, FGD3, TIAM2, DOCK11, FGD4, CYTH4, ARHGEF1, DENND6B, DOCK2, SBF2, RALGPS1
Iron ion binding	2.34	6.79E-03	14	MF	MSMO1, DOHH, CYP7A1, CYP39A1, CYP2W2, CYP11A1, SCD, AGMO, ALOX5, CYP1A2, SC5D, HBAD, PPEF2, XDH
Transmembrane transporter activity	2.11	1.99E-02	13	MF	SLC46A3, SLC45A1, SLC22A2, SLC16A12, SLC7A14, SLC7A11, SLC22A13L, SLCO1C1, GJA1, SV2C, SLCO1A2, FLVCR2,
Heme binding	1.98	3.11E-02	13	MF	GUCY1B4, ADGB, STEAP4, KCNH7, PTGS2, CYP7A1, CYP39A1, CYB5R4, CYP2W2, CYP11A1, CYP1A2, FLVCR2,
Transmembrane signaling receptor activity	3.37	7.56E-04	12	MF	PIGR, NFAM1, PECAM1, FAS, CHRNA8, TLR21, TLR2A, CD247, CD3E, CD3D, SORL1, TLR3
Phosphatidylinositol-3,4,5-trisphosphate binding	4.84	2.63E-03	7	MF	FCHSD2, CYTH4, ARAP2, BTK, ARAP3, DAPP1, ADAP1L
Signaling adaptor activity	3.39	1.59E-02	7	MF	BANK1, FMR1, TRAF5, WWC2, SARM1, HCLS1, CARD11
FAD binding	3.23	1.99E-02	7	MF	SQLE, STEAP4, TXNRD1, CRY2, MICAL3, DHCR24, XDH
Tubulin binding	3.08	2.45E-02	7	MF	GJA1, STMN4, TPPP, CLASP1, NCALD, PARK2, CLASP2
NAD+ poly-ADP-ribosyltransferase activity	3.87	1.76E-02	6	MF	ART1, PARP4, PARP14, PARP9, PARP12, ART4
Non-membrane spanning protein tyrosine kinase activity	2.98	4.88E-02	6	MF	LYN, FES, LCK, BTK, ABL2, FRK
Actin monomer binding	4.61	2.09E-02	5	MF	MYL4, PRKCE, TMSB15B, MYL3, LMOD3
Cholesterol transfer activity	4.61	2.09E-02	5	MF	GRAMD1B, STARD4, STARD5, MTTP, ABCG1
Peptide antigen binding	10.75	7.20E-04	5	MF	BLB1, TAP2, DMA, DHCR24, B2M
Tumor necrosis factor receptor binding	4.03	3.28E-02	5	MF	CD40LG, BRE, TNFSF15, TRAF5, TNFSF13B
T cell receptor binding	15.48	1.27E-03	4	MF	LCK, CD3E, DOCK2, CD3D
Metalloendopeptidase inhibitor activity	8.60	9.10E-03	4	MF	LXN, TIMP2, NGF, TIMP4
MHC class II protein complex	6.45	2.12E-02	4	MF	IGLL1, BLB1, DMA, B2M
Phospholipase binding	6.45	2.12E-02	4	MF	BANK1, LCK, BTK, PARK2
Intramolecular transferase activity	19.35	7.71E-03	3	MF	LSS

Lanosterol synthase activity	19.35	7.71E-03	3	MF	LSS
NAD+ nucleosidase activity, cyclic ADP-ribose	11.61	2.40E-02	3	MF	BST1, SARM1, TLR2A
Primary methylamine	11.61	2.40E-02	3	MF	IL4I1, MAOB, MAOA
CD4 receptor	11.61	2.40E-02	3	MF	CD74, LCK, IL16
Bile acid transmembrane transporter activity	9.68	3.47E-02	3	MF	SLCO1C1, SLCO1A2, SLC51A
Tumor necrosis factor receptor activity	8.29	4.70E-02	3	MF	TNFRSF18, FAS, TNFRSF11A
Fatty acid elongase activity	8.29	4.70E-02	3	MF	ELOVL5, ELOVL2, ELOVL6

Supplementary file 3. GO enrichment analysis for downregulated DEGs found in the liver of broilers at D7 compared to D0

Term	Enrichme	PValue	Count	Group	Genes
DNA replication	7.71	3.29E-15	25	BP	TOP2A, BLM, RTEL1, PCNA, DSCC1, ORC5, ORC4, CHAF1B, CHTF8, ORC6, ORC1, RBBP7, POLE, GINS1, RRM1, RFC3, RFC4, RFC2, TICRR, RPA3, MCM3, MCM5, DNA2, DTL, MCM2
DNA repair	3.43	8.51E-07	23	BP	RRM1, RTEL1, RFC3, BLM, PARPBP, DCLRE1C, RFC4, RIF1, RFC2, PDS5B, BRCA1, INIP, BRIP1, CHAF1B, RAD51,
Cell division	2.03	9.88E-03	17	BP	TOP2A, SEH1L, BORA, ZWILCH, MIS12, PLK1, HAUS3, KIF11, PDS5B, MASTL, SMC2, CCNA2, WEE1,
DNA damage response	1.95	3.50E-02	13	BP	TOP2A, RIF1, VRK1, MASTL, SAMHD1, CHD2, INIP, ALKBH8, HELB, RAD51,
Double-strand break repair via homologous recombination	3.10	1.60E-03	12	BP	RAD52, BLM, RAD51, POLN, RPA3, TOP3A, MEIOB, BRCA1, SAMHD1, BRCA2, INIP, RAD54B
Mitotic cell cycle	2.83	3.26E-03	12	BP	CDT1, TUBA3E, WEE1, SPATA17, GSG2, PPP2R2D, PLK1, MIS12,
DNA replication initiation	7.99	8.80E-06	9	BP	ORC5, ORC4, ORC6, CDC45, ORC1, MCM3, MCM5, MCM6, MCM2
Regulation of DNA replication	6.12	2.37E-04	8	BP	ORC5, CCNA2, PCNA, DSCC1, ACTL6A, GMNN, ESCO2, UCHL5
Telomere maintenance	4.80	1.14E-03	8	BP	RTEL1, BLM, DCLRE1C, RIF1, ACTL6A, TERF1, UCHL5, ATR
Double-strand break repair	3.35	9.21E-03	8	BP	DCLRE1C, POLN, EME1, ZGRF1, TDP1, ESCO2, TRIP13, ATR
Cellular response to ionizing	5.36	1.59E-03	7	BP	BARD1, BLM, RAD51, FIGNL1, BRCA1, RAD9B, BRCA2
DNA	3.05	2.62E-02	7	BP	PSMC3IP, RTEL1, BLM, DCLRE1C,
Chromosome segregation	2.88	3.36E-02	7	BP	TOP2A, MIS12, KIF2C, TTK, ESCO2, BRCA1, DLGAP5
Attachment of mitotic spindle microtubules to kinetochore	12.11	6.69E-05	6	BP	CDT1, SEH1L, NUF2, MIS12, KIF2C, CENPC
Mitotic sister chromatid	7.83	7.15E-04	6	BP	CDC20, CHTF8, DSCC1, GSG2, ESCO2, PDS5B
Positive regulation of cell-substrate adhesion	5.55	3.78E-03	6	BP	CCDC80, ABI3BP, VWC2, NID1, FBLN2, CYR61
Interstrand cross-link repair	4.76	7.54E-03	6	BP	RAD51, DCLRE1C, POLN, FAAP24, FANCC, FANCG
Positive regulation of G1/S transition of mitotic cell	4.30	1.17E-02	6	BP	RRM1, RRM2, RGCC, CENPJ, SASS6, EGFR
Cellular response to UV	3.70	2.16E-02	6	BP	CREBBP, PCNA, MYC, PBK, NOC2L, ATR
Rhythmic process	3.25	3.57E-02	6	BP	TOP2A, CREBBP, DNM1L, PPARGC1A,
Double-strand break repair via break-induced	11.10	6.86E-04	5	BP	CDC45, MCM3, MCM5, MCM6, MCM2

Negative regulation of gene expression via chromosomal CpG island methylation	10.09	1.04E-03	5	BP	HELLS, USP7, UHRF1, MYC, BRCA1
Mitotic spindle assembly checkpoint	5.04	1.56E-02	5	BP	ZWILCH, GSG2, PLK1, TTK, TRIP13
DNA-templated DNA replication	4.44	2.42E-02	5	BP	RFC3, RFC4, POLN, RFC2, POLE
Mitotic G2 DNA damage checkpoint signaling	3.58	4.88E-02	5	BP	BLM, PLK1, BRCA1, DTL, TICRR
Base-excision	4.27	2.76E-02	5	BP	FEN1, NEIL3, RPA3, DNA2, UNG
Protein localization to	8.88	8.58E-03	4	BP	MTBP, ZWILCH, MIS12, TTK
Negative regulation of DNA	8.88	8.58E-03	4	BP	GMNN, TERF1, GTPBP4, ATR
RRNA methylation	8.88	8.58E-03	4	BP	DIMT1, TFB2M, TFB1M, FTSJ3
Protein heterotetramerization	8.07	1.14E-02	4	BP	RRM1, RRM2, FARSA, FARSB
Kinetochore assembly	6.83	1.85E-02	4	BP	MIS12, CENPK, DLGAP5, CENPC
Microtubule depolymerization	6.83	1.85E-02	4	BP	KIF18A, STMN3, KIF2C, NCKAP5
Isotype switching	6.34	2.28E-02	4	BP	ATAD5, EXO1, SWAP70, UNG
Positive regulation of DNA replication	6.34	2.28E-02	4	BP	PCNA, ATAD5, DNA2, EGFR
Negative regulation of intrinsic apoptotic	5.55	3.28E-02	4	BP	HELLS, FIGNL1, NOC2L, MMP9
Reciprocal meiotic recombination	4.93	4.47E-02	4	BP	PSMC3IP, RAD51, TRIP13, RAD54B
Mitotic telomere maintenance via semi-conservative replication	22.19	5.88E-03	3	BP	RTEL1, PCNA, DNA2
Cellular response to glucagon	13.32	1.85E-02	3	BP	GCG, PCK1, ADCY8
DTMP biosynthetic	13.32	1.85E-02	3	BP	DCTD, DUT, TYMS
Very-low-density lipoprotein particle remodeling	11.10	2.69E-02	3	BP	LIPC, LPL, APOA5
Inositol metabolic process	11.10	2.69E-02	3	BP	PPIP5K1, PPIP5K2, IMPA2
Negative regulation of protein processing	11.10	2.69E-02	3	BP	LRRK2, IL1R2, CHAC1
Arginine metabolic process	11.10	2.69E-02	3	BP	ARG2, ASL1, SLC39A8
DNA replication checkpoint	11.10	2.69E-02	3	BP	CDT1, DNA2, RAD9B

Regulation of DNA-templated DNA replication	11.10	2.69E-02	3	BP	CDT1, GMNN, TICRR
Establishment of centrosome localization	9.51	3.65E-02	3	BP	DLG1, CEP83, EZR
Female meiosis I	9.51	3.65E-02	3	BP	MEIOB, TRIP13, MLH3
Cytoplasm	1.26	1.78E-04	197	CC	LRRC14B, OXTR, CNTF, PID1, LGALSL, STMN3, ALKBH8, SMC2, HYKK, DPYSL4, SNRPD1, CHAC1, IL1R2, ARMC9, TALDO1, PASK, WDR77, MYL2, FEZ2, DNA2, OBSL1, SLBP, SPTBN5, PARPBP, PHYHIPL, PRPSAP1, DGUOK, BRIP1, HSPH1, PCBP3, THL, STXBP5, CREBBP, DTNA, FZD2, PLK1, CDC7, FUBP3, TDP1, PPP1R1B, SASS6, CEP83, FARSA, FARSB, TOP2A, TENM4, CCDC125, OLA1, BRCA1, OSBPL10, KIF15, SYNCRIP, IMPA2, AIFM2, GSG2, NUF2, DYL1, TK1, MCF2L2, DLGAP5, NDC1, ARG2, TESC, NSUN6, GAB2, KIF7, UCHL5, CIT, RNF144A, KIF1BP, KIF2C, FAM193B, CRYAB, CRB2, FAXC, ADCY1, ARNTL2, FAM105A, TUBA3E, BGLAP, PAK6, PCNT, SH3BGR, GINS1, CYP2AC7, FAM46B, GAD2, RANBP9, GCG, NPM3, FABP5, KIF26A, DLC1, SERBP1, CCNG1, RIF1, NDUFA10, GMNN, ESPN, FBXO22, ALDH1L2, KLHL30, CAPN9, MEIOB, PIM3, TGM6, PLS1, CA13, BORA, SWAP70, TPM2, [illegible]

Nucleus	1.18	5.49E-03	189	CC	DCLRE1C, RIF1, DSCC1, PRDM6, GMNN, NOC2L, FBXO22, SMC2, ALKBH8, HYKK, EME1, MEIOB, USP7, BORA, ZGRF1, FST, SWAP70, MAGI2, UBE2E1, TALDO1, VRK1, ESCO2, PASK, INIP, WDR77, FTSJ3, NEIL3, MYCN, TCERG1L, RBP2, PKP2, DNA2, TOX, HOXB6, BLM, RTEL1, PARPBP, CBFB, EIF5A2, TSHZ2, CSTF2, TCF7, CDCA7, PDS5B, PHB, AK6, ACACB, GLIS1, ORC5, ORC4, BRIP1, HSPH1, PCBP3, ORC1, DEAF1, BAG3, PRDM16, PPARGC1A, RAI2, IP6K2, UBE2F, CDT1, CREBBP, ZFHX3, HELQ, PLK1, TDRD9, RITA1, CDC7, ESRRG, MT3, PHC3, NFKBIA, DLG1, KIF18A, NASP, FUBP3, TDP1, BHLHE40, PHF14, TADA1, NOX4, FGF12, MDM1, ATR, TOP2A, CUEDC2, TENM2, FEN1, HSP90AB1, TENM4, BNC1, CDCA7L, HSPB7, KIF11, BRCA1, CHD2, BRCA2, HOXC11, SYNCRIP, SCML2, CHAF1B, PLCZ1, EXO1, GSG2, PPP6R2, SNAPC4
Cytosol	1.19	1.93E-02	132	CC	UHRF1BP1L, OXTR, ZFYVE9, GMNN, IRS4, ASL1, NOC2L, ALKBH8, CDC20, HELB, HYKK, DPYSL4, PPIP5K1, PPIP5K2, SNRPD1, FAM162A, FAM110B, PIM3, CHAC1, RGS6, PLS1, CA13, USP7, DIMT1, ACOT12, TALDO1, VRK1, PASK, GTPBP4, WDR77, RBP2, TIPRL, MAP1A, TBC1D23, EZR, EPHA3, CHODL, PFKFB4, BLM, HPCA, AK1, CDCA7, ARHGAP18, HMMR, CPLX1, GIGYF2, ORC5, DGUOK, ORC4, HSPH1, BAG3, PRDM16, PIP5K1B, STXBP5, PCK1, PPARGC1A, UBE2F, BTF3, KLHDC8B, FANCC, ARHGAP26, FANCG, NFKBIA, AKR1B10, PDE3A, TADA1, FARSA, MDM1, CUEDC2, PTPRS, HSP90AB1,

Nucleoplasm	1.46	1.33E-05	122	CC	FOXA1, ATP8A2, DCLRE1C, RIF1, MAML1, DSCC1, GMNN, ZDHHC5, NOC2L, ALDH1L2, SMC2, ALKBH8, CDC20, MYC, NEK2, DIMT1, ACOT12, ESCO2, INIP, GTPBP4, WDR77, FTSJ3, NEIL3, MYCN, RFX5, ADAM12, PKP2, DNA2, EPHA3, FKBP5, DAP3, PARPBP, YTHDC1, CDCA7, NOLC1, PDS5B, PHB, ORC5, ORC4, BRIP1, ORC6, HSPH1, DEAF1, BAG3, PRDM16, IP6K2, CPSF4, PRELID1, ACBD5, CREBBP, DTNA, NOP16, CDC7, PHC3, FANCG, TBL3, RGCC, NASP, TDP1, TADA1, TOP2A, CUEDC2, FEN1, BNC1, CDCA7L, WDR3, HSPB7, BRCA1,
Extracellular region	1.35	1.83E-02	57	CC	HSP90AB1, DHRS11, COL14A1, ISM1, CXCL14, CHRDL1, CYR61, HAPLN1, MDK, RSPO3, PROK1, IGFBP1, IGFBP2, VWDE, APOA5, MMP9, PDGFRL, COL2A1, CCDC80, CRISPLD1, RBP3, PAPPA, COL4A5, PLA2R1, MATN2, FBN2, LAMA2, ITIH6, LTBP2, PTN, NID1, TFPI, FBLN2, THBS4, INS,
Nucleolus	1.81	2.92E-04	42	CC	TOP2A, POP5, FEN1, BLM, SF3B3, DDX24, NIP7, WDR3, OLA1, FOXI1, NOLC1, NAT10, NOC2L, ARNTL2, FBXL22, RRP9, SMC2, ORC4, PLCZ1, MYC, E2F8, WDR36, NOP16, SURF2,
Centrosome	1.46	3.05E-02	33	CC	CHODL, SDCCAG3, PCNA, OLA1, SLC1A4, HMMR, AK6, BRCA2, CDC20, CDC45, GSG2, FAM110B, NUF2, LEO1, NEK2, RTTN, DYL1, PCNT, CKAP2L, PLK1, HAUS3, MASTL, NIN, RGCC,
Chromatin	2.05	1.61E-03	24	CC	BRD3, PCNA, PARPBP, RIF1, DSCC1, PLK1, TCF7, FAAP24, FANCC, VRK1, ESCO2, PDS5B, CHD2, FANCG, SMC2,
Apical plasma membrane	1.77	3.22E-02	17	CC	CRB2, ABCG8, AMN, ABCG5, SLC22A5, ABCB1LA, LHFPL5, ADCY8, USH2A, SLC9A3R2, SLC6A6, DLG1,
Kinetochore	3.50	8.93E-05	15	CC	CDT1, SEH1L, MIS12, PLK1, TTK, CENPC, CDC20, MTBP, KIF18A, NUF2,
Chromosome	2.40	8.04E-03	13	CC	TOP2A, USP7, BLM, TOP3A, BRCA1, NOC2L, FTSJ3, SMC2, RSL1D1, RAD51,
Basement membrane	3.59	9.02E-04	11	CC	DLG1, CCDC80, LAMA2, MDK, VWC2, TIMP3, COL4A5, LAMB1, NID1,
Chromosome, telomeric region	2.91	4.40E-03	11	CC	SUN2, ORC4, FEN1, RTEL1, BLM, RAD51, RIF1, RBBP7, DNA2, TERF1,
Ciliary basal body	2.00	4.90E-02	11	CC	SDCCAG3, CDC45, CENPJ, ARMC9, RTTN, TTLL11, CFAP206, EFHC2,
Site of double-strand break	2.68	2.90E-02	8	CC	HELB, RAD51, RIF1, ZGRF1, RPA3, ESCO2, SAMHD1, INIP
CMG complex	17.85	6.46E-06	6	CC	GINS1, CDC45, MCM3, MCM5, MCM6,
Ctf18 RFC-like complex	14.88	1.88E-04	5	CC	RFC3, CHTF8, RFC4, RFC2, DSCC1

Nuclear origin of replication recognition	11.90	5.27E-04	5	CC	ORC5, ORC4, ORC6, ORC1, MCM2
Lateral element	10.58	5.11E-03	4	CC	BLM, RAD51, BRCA1, BRCA2
MCM complex	9.52	7.07E-03	4	CC	MCM3, MCM5, MCM6, MCM2
Condensed chromosome	5.60	3.22E-02	4	CC	TOP2A, RAD51, RIF1, SMC2
AMPA glutamate receptor complex	5.60	3.22E-02	4	CC	GRIA2, VWC2, SHISA6, GRIA4
Nuclear chromosome	5.29	3.75E-02	4	CC	TOP2A, RAD51, FIGNL1, SMC2
BRCA1-B	17.85	9.97E-03	3	CC	BARD1, BRIP1, BRCA1
DNA replication factor C complex	11.90	2.36E-02	3	CC	RFC3, RFC4, RFC2
ATP binding	1.82	4.23E-09	99	MF	ATP8A2, SMC2, HELB, PPIP5K1, PPIP5K2, PDK4, NEK2, PIM3, EPHA5, ABCG8, ABCG5, ENTPD1, MYH15, SWAP70, ACOT12, UBE2E1, MYH1D, VRK1, PASK, MYH1E, DNA2, ROR2, EPHA3, PFKFB4, BLM, RTEL1, SLC22A5, AK1, FIGNL2, AK6, ACACB, DGUOK, BRIP1, HSPH1, ORC1, FIGNL1, PIP5K1B, UBE2F, HELQ, PLK1, TDRD9, CDC7, ABCA8, KIF18A, FARSA, ATR, FARSB, TOP2A, HSP90AB1, LRRK2, MAST2, LRRK1, OLA1, NAT10, KIF11,
DNA binding	1.41	8.02E-03	56	MF	TOP2A, FEN1, BRCA1, CHD2, BRCA2, HOXC11, EME1, EXO1, AIFM2, MYC, SNAPC4, DNMT3B, MEIOB, POLE, RFC3, ZHX3, RFC4, RFC2, SWAP70, TOP3A, RFX5, MCM3, PKP2, DNA2, MCM6, TOX, HMX1, MCM2, RTEL1, BLM, PARPBP, PCNA, UHRF1, TSHZ2, TCF7, PDS5B, ARNTL2, RAD54B
ATP hydrolysis activity	2.46	2.04E-05	29	MF	BLM, HSP90AB1, ATP8A2, ABCB1LA, OLA1, FIGNL2, DNAH9, CHD2, AK6, SMC2, ORC4, BRIP1, ORC1, FIGNL1, ABCG8, RFC3, ABCG5, RFC4, ATAD5,
Chromatin binding	1.94	2.63E-03	25	MF	FOXA1, TOP2A, PCNA, GMNN, FAAP24, ASCL1, CHD2, EGFR, SMC2, CDC45, ORC1, EXO1, MEIOB, POLE, BRD3, HELLS, CDT1, CREBBP,
Single-stranded DNA binding	4.37	3.03E-06	16	MF	BLM, TOP3A, SAMHD1, BRCA2, RAD52, NEIL3, CDC45, RAD51, FUBP3,
Microtubule binding	1.72	4.61E-02	16	MF	SGIP1, SPAG6, MAST2, PLK1, KIF11, KIF7, KIF15, KIF18A, KIF26A, MAP1A,
Magnesium ion binding	1.93	2.37E-02	15	MF	TOP2A, FEN1, DUT, ATP8A2, TESC, LRRK2, MAST2, AK1, PLK1, PRPSAP1,
Single-stranded DNA helicase activity	12.29	4.18E-09	11	MF	HELB, RFC3, RAD51, CHTF8, RFC4, RFC2, DSCC1, MCM3, MCM5, DNA2, MCM2
Heparin binding	2.50	1.85E-02	10	MF	ADAMTS15, PTPRS, LIPC, GPNMB, MDK, ABI3BP, RSPO3, LPL, PTN,

DNA clamp loader activity	4.91	3.98E-04	9	MF	BRIP1, RFC3, BLM, CHTF8, RFC4, RFC2, DSCC1, MCM3, DNA2
Tubulin binding	3.73	1.03E-02	7	MF	TPPP2, LRRK2, CENPJ, RITA1, STMN3,
DNA replication origin binding	10.83	1.33E-04	6	MF	ORC5, ORC4, CDC45, ORC1, MCM5, MCM2
Single-stranded 3'-5' DNA helicase activity	6.70	1.58E-03	6	MF	BLM, HELQ, MCM3, MCM5, MCM6, MCM2
4 iron, 4 sulfur cluster binding	3.61	2.39E-02	6	MF	RTEL1, BRIP1, TYW1, NDUFS1, DNA2, POLE
Damaged DNA binding	3.35	3.19E-02	6	MF	NEIL3, CREBBP, PCNA, DCLRE1C, RPA3, UNG
Microtubule motor activity	3.27	3.49E-02	6	MF	KIF18A, KIF26A, KIF2C, KIF11, KIF7, KIF15
DNA polymerase binding	7.82	3.05E-03	5	MF	CDT1, RTEL1, RAD51, PCNA, NAT10
Forked DNA-dependent helicase activity	5.87	9.13E-03	5	MF	BLM, MCM3, MCM5, MCM6, MCM2
Four-way junction helicase activity	5.33	1.29E-02	5	MF	BLM, MCM3, MCM5, MCM6, MCM2
Double-stranded DNA helicase activity	5.33	1.29E-02	5	MF	BLM, MCM3, MCM5, MCM6, MCM2
Protein phosphatase	5.10	1.51E-02	5	MF	TIPRL, PPP1R1B, IGFBP2, PTN, PPP1R14D
CCR6 chemokine receptor binding	6.70	1.97E-02	4	MF	AVBD4, AVBD6, AVBD1, AVBD7
RRNA (adenine-N6,N6-)-dimethyltransferase activity	17.60	1.02E-02	3	MF	DIMT1, TFB2M, TFB1M
Inositol hexakisphosphate kinase activity	14.08	1.66E-02	3	MF	PPIP5K1, PPIP5K2, IP6K2
5'-flap endonuclease	11.73	2.42E-02	3	MF	FEN1, EXO1, DNA2
5'-3' DNA helicase activity	8.80	4.27E-02	3	MF	HELB, BRIP1, DNA2
Hydrolase activity, acting on acid anhydrides, in phosphorus-containing anhydrides	8.80	4.27E-02	3	MF	RTEL1, BRIP1, BLM

Supplementary file 4 . KEGG enrichment analysis for upregulated DEGs found in the liver of broilers at D7 compared to D0				
Term	Genera	PValue	Count	Input
Metabolic pathways	0.078	3.24E-11	101	PTGS2|IDI1|GCLC|CDO1|DHCR7|ASS1|SQLE|CERS4|GPAM|HKDC1|NDST4|INPP4B|NADK2|ETNK2|FDPS|ALOX5|HMGCR|AGXT2|NME4|DBH|ADCY7|INPP5D|HYAL3|GUCY1B4|BST1|ACLY|MVK|NSDHL|MGST3|ACSBG2|PANK3|CYP11A1|SCD|XDH|PNPLA3|PLCH1|LSS|FDFT1|HNMT|B4GALT6|ITPK1|NUDT9|PLCB2|PTGES|CSGALNACT1|PLA2G10|ELOVL6|MAOB|MAOA|ELOVL5|ELOVL2|MSMO1|PIK3CD|AMPD1|SC5D|RBKS|ME1|CHIA|AFMID|ACAT2|FASN|IL4I1|CYP7A1|ATP6V0C|PDHA1|ACACA|MVD|ALDH9A1|ACADSB|FADS1|SDSL|FADS2|CYP1A2|GSTZ1|KL|PDE4C|AACS|ALDH1A1|DGKZ|ACER1|GCNT1|CMPK2|GPX3|DGKK|SARDH|GBE1|PGP|NT5C2|HSD17B7|PIGC|CA12|ACMSD|ST6GALNAC1|DHCR24|PMVK|CA6|AD
Steroid biosynthesis	0.588	1.96E-08	10	MSMO1|LIPA|NSDHL|DHCR7|SC5D|LSS|HSD17B7|FDFT1|SQLE|DHCR24
Fatty acid metabolism	0.208	1.87E-05	11	ACACA|ACADSB|SCD|ELOVL6|ACAT2|ELOVL5|ELOVL2|ACSBG2|FADS1|FASN|FAD
Intestinal immune network for IgA production	0.25	3.06E-05	9	BLB1|ICOS|CD28|CD40LG|IL15|PIGR|CCR9|ICOSLG|TNFSF13B
Terpenoid backbone	0.368	3.07E-05	7	FDPS|IDI1|PMVK|ACAT2|MVD|HMGCR|MVK
Cell adhesion molecules (CAMs)	0.131	4.68E-05	16	PECAM1|ICOS|ITGB2|CD40LG|NTNG1|BF2|NRCAM|ALCAM|NRXN3|BLB1|YF5|CD28|PTPRC|CD2|PDCD1LG2|ICOSLG
Tryptophan metabolism	0.205	0.000276	8	CYP1A2|AFMID|ACAT2|MAOB|MAOA|ALDH9A1|IL4I1|ACMSD
Cytokine-cytokine receptor interaction	0.094	0.000525	19	IL22|IL2RA|LIF|CCL1|CD40LG|TNFSF15|FAS|CSF1R|NGF|IL16|IL15|INHBB|XCL1|TNFRSF21|TNFRSF11A|CCR7|CCR9|TNFRSF18|TNFSF13B
Calcium signaling pathway	0.094	0.000969	17	AVPR1B|PLCB2|ADCY7|CCKAR|ATP2A3|PDE1A|MYLK|CACNA1G|HTR7|TACR2|TNNC1|ITPR3|SLC8A3|NTSR1|MCU|GRM1|P2R
PPAR signaling pathway	0.141	0.001347	9	FADS2|SCD|PPARG|ANGPTL4|ME1|ACSBG2|SLC27A6|CYP7A1|PLIN2
Biosynthesis of unsaturated fatty acids	0.2	0.001797	6	SCD|ELOVL6|ELOVL5|ELOVL2|FADS1|FADS2
Tyrosine	0.176	0.003129	6	DBH|GSTZ1|MAOB|MAOA|ADH1C|IL4I1
Apelin signaling pathway	0.101	0.003168	12	PLCB2|ADCY7|GNG10|MYL4|PRKCE|MYLK|MEF2C|PIK3R5|SPP1|MYL3|ITPR3|SLC8A
Neuroactive ligand-receptor interaction	0.072	0.003304	24	AVPR1B|NTSR1|GABBR2|P2RX7|RXFP3|BRS3|GRM1|TSPO2|GRID1|GRP|CALCRL|HTR7|NPY2R|OPRK1|P2RY6|TACR2|GZMA|C3AR1|NPY1R|CCKAR|GALR1L|HTR1D|F2RL1|HCRTR2

Herpes simplex virus 1 infection	0.087	0.007072	13	CD74|BLB1|TAP2|TRAF5|BF2|FAS|PIK3CD|BAK1|YF5|B2M|PIK3R1|TLR3|TLR2A
Vascular smooth muscle contraction	0.094	0.007422	11	KCNMB1|AVPR1B|PLCB2|ADCY7|PLA2G10|CALCRL|PRKCE|MYLK|KCNMA1|GUCY1B4|ITPR3
Valine, leucine and isoleucine degradation	0.136	0.009522	6	AGXT2|ACAT2|ALDH9A1|ACADSB|AACS|IL4I1
Pyruvate metabolism	0.156	0.010711	5	ACACA|ACAT2|PDHA1|ME1|ALDH9A1
Phosphatidylinositol signaling system	0.099	0.011074	9	DGKZ|ITPK1|PLCB2|INPP5D|PIK3CD|INPP4B|DGKK|PIK3R1|ITPR3
Fatty acid degradation	0.152	0.011971	5	ACAT2|ACADSB|ADH1C|ALDH9A1|ACSBG2
Histidine	0.19	0.012205	4	MAOB|MAOA|ALDH9A1|HNMT
Lysosome	0.091	0.012766	10	LIPA|ATP6V0C|GM2A|LAPTM4B|LITAF|AP3M2|LAPTM5|GGA3|CTSS|M6PR
Phagosome	0.083	0.01618	11	BLB1|TAP2|BF2|ITGB2|YF5|MARCO|TLR2A|CTSS|TUBB2A|ATP6V0C|M6PR
Glycine, serine and threonine metabolism	0.135	0.018011	5	MAOB|MAOA|SARDH|SDSL|AGXT2
ECM-receptor interaction	0.096	0.018365	8	LAMA1|COL9A3|AGRN|FREM2|COL4A4|SPP1|COL4A3|SV2C
Gap junction	0.095	0.019492	8	GJA1|PLCB2|ADCY7|GUCY1B4|MAP2K5|ITPR3|TUBB2A|GRM1
Phenylalanine metabolism	0.214	0.022633	3	MAOB|IL4I1|MAOA
Wnt signaling pathway	0.078	0.024246	11	WNT3A|PLCB2|RAC2|FZD5|AXIN1|TCF7L2|DKK1|WIF1|WNT11B|MMP7|WNT4
Cellular senescence	0.077	0.026377	11	CDKN2B|BF2|PIK3CD|GADD45B|CCNE1|RASSF5|YF5|PIK3R1|TRPV4|ITPR3|MCU
Purine metabolism	0.08	0.026769	10	NME4|NUDT9|ADCY7|AK3|AMPD1|XDH|GUCY1B4|NT5C2|PDE4C|PDE1A
MAPK signaling pathway	0.065	0.027679	17	NGF|NTF3|RAC2|FAS|GADD45B|CSF1R|EFNA5|CACNA1G|MEF2C|IGF1|EREG|MAP2K5|KITLG|PTPN7|FGF23|PTPN5|FLT1
Melanogenesis	0.088	0.028788	8	WNT3A|PLCB2|ADCY7|FZD5|TCF7L2|WNT4|WNT11B|KITLG
Cysteine and methionine metabolism	0.116	0.030356	5	IL4I1|AGXT2|SDSL|CDO1|GCLC
Fatty acid biosynthesis	0.176	0.034999	3	FASN|ACACA|ACSBG2
Arachidonic acid metabolism	0.102	0.047003	5	PTGES|PTGS2|ALOX5|PLA2G10|GPX3
p53 signaling pathway	0.091	0.048034	6	IGF1|SESN3|FAS|GADD45B|CCNE1|CCNG2
Drug metabolism - cytochrome P450	0.118	0.049166	4	MGST3|MAOA|ADH1C|MAOB
Ferroptosis	0.118	0.049166	4	GCLC|CP|SLC7A11|ATG7

Supplementary file 5. KEGG enrichment analysis for downregulated DEGs found in the liver of broilers at D7 compared to D0				
Term	Generatio	Coun	PValue	Input
Metabolic pathways	0.051938	67	4.47E-05	ASL1|AOC3|PCK1|PI4KB|B4GALNT3|PLCZ1|HYKK|AK6|MAN1B1|RRM2|RRM1|PDE3A|SRRL|URAH|ST3GAL3|DCK|TYMS|DUT|NAT8L|TALDO1|CBR1|FAHD1|NDUFS1|NDUFA10|GAD2|DCTD|MECR|ACOT12|HSD17B3|NDUFA11|DNMT3B|B3GALT5|LIPC|AMY2A|ENTPD1|ACACB|GUSB|CA13|CA9|SRM|IMPA2|PIGS|UAP1L1|PIGU|TK1|UPP1|BLVRA|ALG14|PDE10A|UPP2|LCT|PCYT1B|BPNT1|ADCY1|ARSB|PIP5K1B|AK1|ADCY8|PFKFB4|CA4|PDE1C|THL|NDUFS4|FOLH1|GPX7|CHAC1|ARG2
Cell cycle	0.192982	22	1.19E-10	PLK1|ATR|CDC7|CCNA2|TTK|WEE1|CUL1|PCNA|RBL1|ORC6|ORC4|ORC5|ORC1|MCM6|MCM5|MCM3|MCM2|MYC|CCNB2|CDC20|CDC45|CREBBP
Calcium signaling pathw	0.083333	15	8.41E-04	ADORA2B|ATP2B2|ADRA1D|PDE1C|PLCZ1|RYR3|OXTR|EGFR|CAMK2A|SLC25A4|ADCY8|CHRNA7|ASPH|DRD1|ADCY1
Fanconi anemia pathway	0.26	13	3.78E-08	BRCA1|FANCG|BRCA2|EME1|RPA3|BLM|ATR|TOP3A|RAD51|BRIP1|POLN|FAAP24|FANCC
DNA replication	0.387097	12	3.18E-09	PCNA|RPA3|DNA2|RFC4|MCM5|RFC3|RFC2|MCM6|POLE|MCM3|MCM2|FEN1
Purine metabolism	0.096	12	8.86E-04	ADCY1|ENTPD1|PDE1C|PDE3A|AK1|ADCY8|AK6|RRM2|RRM1|PDE10A|URAH|DCK
Wnt signaling pathway	0.085106	12	2.29E-03	RSPO3|FZD2|LGR6|NOTUM|CAMK2A|ROR2|SERPINF1|TCF7|VANGL1|MYC|CUL1|CREBBP
Homologous recombinat	0.289474	11	1.70E-07	BRCA1|BRCA2|RAD54B|BARD1|TOP3A|RPA3|BLM|RAD52|RAD51|BRIP1|EME1
Adrenergic signaling in c	0.085938	11	3.19E-03	ADCY1|ADRA1D|PPP2R2D|PPP2R3A|ADCY8|CAMK2A|PPP2R2C|TPM2|MYL2|ATP2B2|MYH1
Pyrimidine metabolism	0.169492	10	3.82E-05	UPP2|UPP1|DCK|ENTPD1|DCTD|TYMS|RRM2|RRM1|TK1|DUT
Tight junction	0.064103	10	2.85E-02	DLG1|PCNA|DLG2|TUBA3E|PPP2R2D|PPP2R2C|EZR|MYH1D|MYH1E|MYL2
Oocyte meiosis	0.090909	9	5.24E-03	CCNB2|ADCY1|PLK1|PLCZ1|ADCY8|CAMK2A|CDC20|AURKA|CUL1
Ribosome biogenesis in e	0.119403	8	1.82E-03	RPP38|WDR3|AK6|GTPBP4|WDR36|TBL3|POP5|NAT10
Apelin signaling pathway	0.067227	8	3.78E-02	ADCY1|RYR3|ADCY8|PPARGC1A|GNG13|GNG4|MYL2|CTGF
Mismatch repair	0.35	7	1.12E-05	PCNA|RFC4|EXO1|MLH3|RFC3|RFC2|RPA3
Drug metabolism - other	0.122807	7	3.00E-03	UPP2|UPP1|RRM2|RRM1|TK1|DUT|GUSB
Adipocytokine signaling	0.109375	7	5.36E-03	CPT1A|PCK1|ACACB|IRS4|NFKBIA|PPARGC1A|POMC
Progesterone-mediated o	0.089744	7	1.39E-02	CCNB2|CCNA2|ADCY1|PLK1|ADCY8|HSP90AB1|AURKA
ECM-receptor interactio	0.084337	7	1.86E-02	HMMR|LAMA2|THBS4|COL4A5|LAMB1|TNN|COL2A1
TGF-beta signaling pathw	0.077778	7	2.68E-02	RBL1|ZFYVE9|FST|FMOD|MYC|CUL1|CREBB
Phosphatidylinositol sign	0.076923	7	2.81E-02	PI4KB|PLCZ1|PPIP5K2|IMPA2|PPIP5K1|PIP5K1B|IP6K2

Melanogenesis	0.076923	7	2.81E-02	TCF7|ADCY1|ADCY8|FZD2|CAMK2A|POMC|CREBBP
Nucleotide excision repa	0.157895	6	1.91E-03	PCNA|RFC4|RPA3|RFC2|POLE|RFC3
PPAR signaling pathway	0.09375	6	1.85E-02	CPT1A|PCK1|LPL|FABP2|FABP5|APOA5
p53 signaling pathway	0.090909	6	2.10E-02	CCNB2|EI24|CCNG1|ATR|RRM2|AIFM2
Base excision repair	0.172414	5	3.24E-03	PCNA|POLE|FEN1|NEIL3|UNG
ABC transporters	0.125	5	1.09E-02	ABCG8|ABCB10|ABCB1LA|ABCA8|ABCG5
Glutathione metabolism	0.106383	5	1.95E-02	CHAC1|RRM1|RRM2|GPX7|SRM
Alanine, aspartate and gl	0.117647	4	2.65E-02	ASL1|NAT8L|GAD2|FOLH1
Hedgehog signaling path	0.097561	4	4.54E-02	KIF7|CDON|CUL1|HHIP
Nitrogen metabolism	0.176471	3	0.020961	CA9|CA13|CA4
